# Adapting the Planetary Health Diet Index for children and adolescents

**DOI:** 10.1186/s12966-023-01516-z

**Published:** 2023-12-14

**Authors:** Carolina Venegas Hargous, Liliana Orellana, Claudia Strugnell, Camila Corvalan, Steven Allender, Colin Bell

**Affiliations:** 1 Deakin University, Global Centre for Preventive Health and Nutrition (GLOBE), Institute for Health Transformation, Geelong, Australia; 2 Deakin University, School of Medicine, Faculty of Health, Geelong, Australia; 3 Deakin University, Biostatistics Unit, Faculty of Health, Geelong, Australia; 4 Deakin University, Institute for Physical Activity and Nutrition (IPAN), Geelong, Australia; 5 University of Chile, Institute of Nutrition and Food Technology (INTA), Santiago, Chile

**Keywords:** EAT-Lancet diet, Sustainable diet, Nutrition, Environmental sustainability, Dietary index, Children, Adolescents

## Abstract

**Background:**

Tools for measuring adherence to sustainable healthy diets among children and adolescents are lacking.

**Objective:**

To advance methods for measuring adherence to sustainable healthy diets among children and adolescents by adapting an existing index, compare scores obtained using the original and adapted versions of the index in a sample of Chilean children, and describe the adapted index association with diet characteristics.

**Methods:**

The Planetary Health Diet Index (PHDI) was adapted to better reflect children's and adolescents’ nutritional requirements. The adapted index (PHDI-C) comprises 16 components with a maximum score of 150 points. PHDI-C was piloted among a sample of 958 Chilean children (3–6 years) using dietary data collected in 2016 through single 24-h recalls. A decision tree and food disaggregation methodology were developed to guide the calculation of scores. Scores obtained using the original and adapted versions of the index were compared. Linear regression models adjusted by child’s gender and age were fitted to explore associations between total PHDI-C score, dietary recall characteristics and nutritional composition of children’s diets.

**Results:**

PHDI accounted for 75.7% of children’s total caloric intake, whereas PHDI-C accounted for 99.6%. PHDI & PHCI-C scores were low among this sample of children; however, mean total score was lower when using PHDI compared to PHDI-C [40.7(12.1) vs 50.1(14.6)]. Children’s scores were very low for *nuts & peanuts, legumes*, *dark green vegetables*, *whole cereals*, *tubers & potatoes*, and *added sugars* components across both indices, but were higher for *dairy products and eggs & white meats* components when using the PHDI-C due to adjustments made to ensure nutritional adequacy. Mean total PHDI-C score was significantly lower on weekends and special occasions, and significantly higher when children reported having a special diet (e.g., vegetarian). Total PHDI-C score was negatively associated with total sugars, saturated fats, trans fats, and animal-based protein intake, and positively associated with total protein, plant-based protein, total carbohydrates, and total fibre intake.

**Conclusions:**

This study provides a replicable method for measuring adherence to sustainable healthy diets among children and adolescents that can be used to monitor trends and measure the effectiveness of actions targeting improving children’s diets.

**Supplementary Information:**

The online version contains supplementary material available at 10.1186/s12966-023-01516-z.

## Introduction

Malnutrition in all its forms, including undernutrition, overnutrition, and diet-related non-communicable diseases [1], is considered the leading risk factor for morbidity and mortality globally [2]. In turn, the negative consequences of climate change are projected to decrease the global food availability by 3.2% per person and fruit and vegetable consumption by 4.0% per person by 2050, leading to 529,000 climate change-related deaths worldwide [3]. These problems are largely driven by the unhealthy and unsustainable ways in which current food systems operate [4]; hence, a significant food system transformation will be required to ensure people’s right to adequate food and the health and sustainability of our planet [5].

This food system transformation should enable consumption of diets that support all aspects of human health, are nutritionally adequate, have a low environmental impact, are affordable, safe, equitable, and culturally acceptable for all [6]. Ideally, such healthy and environmentally sustainable diets (hereafter sustainable healthy diets) should be adopted early in life when long-lasting eating habits are developed [7] and should include safe and clean drinking water, a wide variety of minimally processed foods such as fruit, vegetables, whole grains, nuts, and legumes, and limited ultra-processed products [6]. Occasionally, these diets can include moderate amounts of eggs, dairy, poultry and fish, and small amounts of red meats [6]. To facilitate such food system transformation, the EAT-Lancet Commission proposed a sustainable healthy diet for individuals aged two years and older in 2019 [5]. The diet includes a variety of food groups and a range of caloric intakes to meet children and adults’ nutritional requirements while ensuring consumption patterns stay within planetary boundaries [5]. Countries are encouraged to adapt the EAT-Lancet diet to their specific context and cultural needs and use it for developing dietary recommendations, as well as policies and programs to increase the availability, accessibility, and affordability of healthy and environmentally sustainable foods [5]. The EAT-Lancet diet can also serve as a tool for assessing the nutritional quality and environmental sustainability of populations’ diets, enabling countries to monitor trends and measure the effectiveness of triple-duty actions aimed at addressing obesity, undernutrition, and climate change [5].

Researchers have developed several indices to measure populations’ adherence to the EAT-Lancet diet [8–19]. They include components to account for food groups that should be encouraged (adequacy components) and food groups that should be limited (moderation components). Most have absolute cut-off values (g/day) [8–17] instead of energy-adjusted cut-off values (% of total calories) [18, 19] and use binary [8–11, 18], ordinal [12, 13], or continuous scoring scales [14–17, 19]. The combination of energy-adjusted cut-off values and continuous scoring scales is ideal because energy-adjusted cut-off values allow the index to account for group-specific nutritional requirements compared to absolute cut-off values, and the use of continuous scoring scales increases indices’ ability to discriminate between different levels of diet quality [20]. The only index featuring these two characteristics is the Planetary Health Diet Index (PHDI) developed and validated by Cacau et al. among a representative sample of Brazilian adults [19]. A higher total PHDI score reflects a better adherence to the EAT-Lancet diet and has been significantly associated with higher diet quality [19], lower body mass index and waist circumference [21], lower levels blood pressure, total cholesterol, LDL cholesterol, and non-HDL cholesterol [22], and lower greenhouse gas emissions [19] among a representative sample of Brazilian adults.

Most indices have been used among the adult population, and only a few have been used with children and adolescents [10, 23]. Montejano et al. [10] assessed adherence to the EAT-Lancet diet using a dietary index score among participants from the DONALD (Dortmund Nutritional and Anthropometric Longitudinal Designed) study (≥ 15 years of age), while Marchioni et al. [23] used the Planetary Health Diet Index (PHDI) among a representative sample of the Brazilian population aged 10 years and older. However, none of the indices have been developed to consider the specific nutritional requirements of growing children. This is problematic given the recently estimated micronutrient inadequacies of the EAT-Lancet diet [24]. Beal et al. assessed the micronutrient adequacy of the EAT-Lancet diet for adults (≥ 25 years) and women of reproductive age (15–49 years) and estimated that the EAT-Lancet diet was deficient in vitamin B12, calcium, iron, and zinc, implying that higher intakes of animal-based products are required to achieve micronutrient adequacy among these population groups [24]. Additionally, a study by Lassen et al. [25] using Danish food composition data showed that the EAT-Lancet diet would be deficient in Vitamin A, Vitamin D, Calcium, Iodine, and Selenium for the population aged 6–65 years. Hence, it is likely that higher intakes of animal-based foods such as dairy, eggs, and white meats are also required to achieve micronutrient adequacy for children and adolescents.

To address the need for a tool to measure adherence to sustainable healthy diets among children and adolescents that accounts for the specific nutritional needs of these age groups, this study aimed to 1) advance methods for measuring adherence to sustainable healthy diets among children and adolescents by adapting the PHDI; 2) compare scores obtained using the original (PHDI) and adapted (PHDI-C) versions of the index in a sample of Chilean pre-schoolers; and 3) describe associations between total PHDI-C score, dietary recall characteristics and nutritional composition of children’s diets.

## Methods

### Study design

This study uses cross-sectional pre-existing data collected in 2016 from the Food Environment Chilean Cohort (FECHiC) to demonstrate the applicability of the PHDI-C. The original study was established in 2016 by researchers at the Institute of Nutrition and Food Technology (INTA) to assess the impact of Chile’s Food Labelling and Advertising Law [26].

### Participants

A convenience sample of 961 children aged 3–6 years were recruited from public kindergartens located in low-medium income neighbourhoods of south-eastern Santiago, Chile. The recruitment process and inclusion and exclusion criteria have been described in detail elsewhere [27].

### Collection of dietary data

Between April and August 2016, trained dietitians collected FECHiC participants’ dietary intake data from a nominated primary caretaker (usually mothers) through a single 24-h recall. Dietitians followed the United Stated Department of Agriculture (USDA) automated multiple pass method [28] and utilized a software specifically developed for the collection of dietary data (SER 24). This method allowed the collection of specific information on food items including portion size, cooking method, meal occasion (e.g. breakfast, lunch, dinner), mealtime, brand, flavour, and other details, and prompted participants to remember usually forgotten items reducing the risk of recall bias [29]. A photographic atlas of typical Chilean foods and culinary preparations [30] was used to obtain accurate estimation of portion size and enhance food recall. Dietary recall characteristics were also collected and included: day of the dietary recall (weekday vs weekend/holiday), type of eating pattern (typical vs atypical (because of celebration, or sickness, or vacation)), type of diet (normal (i.e., omnivorous diet with no dietary restriction of any kind) vs special (i.e., lactose free, gluten free, vegetarian, or vegan diets)), and reliability of the recall (reliable (i.e., recalls with no missing information) vs unreliable (i.e., recalls with missing information on the amount consumed of some food items)). Participants with unavailable dietary data were excluded from this analysis (*n* = 3). The analytical sample was 958 participants (Supplemental Fig. 1).

### Classification of dietary data

Trained dietitians (*n* = 3) from INTA categorized all reported foods and beverages according to their description following a food classification system developed by INTA researchers [31]. Standardized recipes were used to disaggregate culinary preparations into ingredients that were classified accordingly (e.g., for spaghetti and Bolognese sauce, pasta was grouped with cereals, ground beef with meats, tomato sauce with industrialized sauces and dressings, onions and carrots with vegetables, and vegetable oil with oils).

### Linkage of dietary data with nutrient composition data and ingredient list information

Food items reported in children's dietary recalls were linked to a bespoke food composition database developed for Chile by the University of North Carolina and INTA [32]. This food composition database incorporated data from the USDA National Nutrient Database [33] and from the food labels of packaged products available in Chile during 2016 [32].

Minimally processed foods were linked with nutritional information obtained from the USDA National Nutrient Database [33] allowing a maximum 20% variation from the information declared in the Chilean food composition table [34], while packaged products were linked with nutrition information panels and ingredients lists obtained from packaged foods and beverages available in Chile before the implementation of the Food Labelling and Advertising Law (i.e., before June 26, 2016) [26]. This information was gathered as part of the INFORMAS Chile project [35] during the first quarter of 2015 and 2016 [36].

Once reported food items were linked to corresponding nutritional information, we determined the amount of calories, proteins, fats, carbohydrates, sugars, and fibre consumed by each child.

### Development of the Planetary Health Diet Index for children and adolescents (PHDI-C)

With the aim of addressing the need for a tool to measure adherence to sustainable healthy diets among children and adolescents that takes into account their specific nutritional needs, we created a new dietary index based on the PHDI developed and validated by Cacau et al. [19]. The original PHDI has five adequacy components to account for foods that should be encouraged (i.e., *nuts & peanuts*, *legumes*, *whole cereals*, *fruits*, and *vegetables*), two ratio components to promote vegetable variety (i.e., *dark green vegetables ratio* and *red and orange vegetables ratio*), five optimum components to account for foods that should be consumed within an specific range to ensure both diet quality and environmental sustainability (i.e., *eggs, fish & seafood*, *tubers & potatoes, dairy products,* and *vegetable oils*), and four moderation components to account for foods that should be limited (i.e., *red meats, chicken & substitutes*, *animal fats,* and *added sugars*) [19]. Each PHDI component is associated with specific energy-adjusted cut-off values and a continuous scoring scale resulting in a total score ranging from 0 to 150 points [19]. The original PHDI excludes refined cereals, cocoa powder, baking powder, baking soda, yeast, salt, herbs and spices, artificially sweetened beverages, tea, coffee, water, and alcoholic beverages from its components.

Given the concerns regarding the micronutrient adequacy of the EAT-Lancet diet [24, 25] on which the PHDI is based [19], we developed six sample diets that meet the varying energy and nutrient requirements of boys and girls aged 2 to 18 years. Table 1 illustrates a sustainable healthy diet that meets the average caloric requirements for a six-year-old child; Supplemental Tables 1 and 2 illustrate diets that meet the lowest and the highest caloric requirements for children aged 2–12 years, respectively; Supplemental Tables 3 and 4 illustrate diets that meet the lowest and the highest caloric requirements for adolescents aged 13–18 years, respectively; and Supplemental Table 5 illustrates a diet that meets the highest caloric and nutrient requirements for adolescent girls in reproductive age. Energy requirements were calculated based on the FAO 2004 report on Human Energy Requirements [37]. Macronutrients requirements were calculated as 15% of energy from proteins, 35% from fats, and 55% from carbohydrates as per recommended by the Institute of Medicine [38]. This ensured a safe level of protein intake across the range of ages [39], as well as adequate intakes of fibre and essential fatty acids [38]. Lastly, micronutrients requirements were defined based on Dietary Reference Intakes (DRIs) designed to meet the recommendations for 97.5% of healthy children and adolescents [38, 40] (see Table 1 and Supplemental Tables 1–5).

**Table 1:**
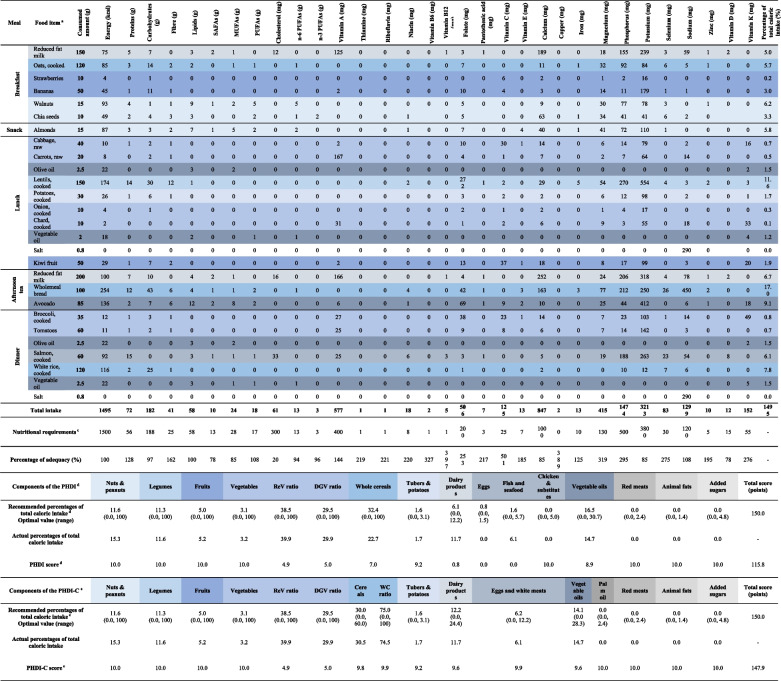
Example of a sustainable healthy diet for a six-year-old child with an average caloric requirement of 1,500 kcal/day

USDA United States Department of Agriculture, SAFA Saturated fatty acids, MUFAs Monounsaturated fatty acids, PUFAs Polyunsaturated fatty acids, PHDI Planetary Health Diet Index, PHDI-C, Planetary Health Diet Index for children and adolescents, ReV ratio, Red and orange vegetables ratio, DGV ratio Dark green vegetables ratio, WC ratio Whole cereals ratio

^a^ Each food item is color-coded with its corresponding index component

^b^ Food items’ nutritional composition was obtained from the USDA National Nutrient Database [33]

^c^ The caloric requirement of 1,500 kcal/day corresponds to the average caloric requirement of a 21.7 kg boy aged 6 years and a 20.6 kg girl aged 6 years whose level of physical activity is moderate to high [37]. Macronutrient requirements were calculated based on acceptable macronutrient distribution ranges [38]. Micronutrient requirements were defined based on Recommended Dietary Allowances or Average Intakes for children aged 4–8-year-old [38, 40]

^d^ Components and scores correspond to the PHDI developed and validated by Cacau et al. [19]. Each component is associated to a recommended range of total caloric intake expressed as percentage of total calories, except for the ratio components which are expressed as percentage of total calories from vegetables. All components can score between 0 to 10 points, except for the ratio components which can score between 0 to 5 points, resulting in a total score of 150 points [19]

^e^ Components and scores correspond to the PHDI-C proposed in this study. Each component is associated to a recommended range of total caloric intake expressed as percentage of total calories, except for the DGV ratio and ReV ratio components which are expressed as percentage of total calories from vegetables, and the WC ratio which is expressed as percentage of total calories from cereals. All components can score between 0 to 10 points, except for the DGV and ReV ratio components which can score between 0 to 5 points, resulting in a total score of 150 points. The formula to calculate the score for each component is provided in Table 2

**Table 2:** Components of the Planetary Health Diet Index for children and adolescents (PHDI-C), recommended percentages of total caloric intake for children and adolescents, and formulae to calculate each component score

PHDI-C Components	Recommended % of total caloric intake for children ^a,b^	Recommended % of total caloric intake for adolescents ^a,b^	Maximum possible score	Formulae to calculate each component score
	**Optimal value (range)**	**Optimal value (range)**		
**Adequacy components**				
Nuts and peanuts	≥ 11.6 (0, 100)	≥ 11.6 (0, 100)	10	$$score\left(x\right)=\left\{\begin{array}{ll}\frac{10\times x}{A}&if\, |x\le A\\ 10&if\, |x>A\end{array}\right.$$ For a given adequacy component, *x* is the percentage of calories consumed and A is the optimal recommended value
Legumes	≥ 11.3 (0, 100)	≥ 11.3 (0, 100)	10	
Fruits	≥ 5.0 (0, 100)	≥ 5.0 (0, 100)	10	
Vegetables	≥ 3.1 (0, 100)	≥ 3.1 (0, 100)	10	
**Ratio components**				
Dark green vegetables ratio	29.5 (0.0, 100)	29.5 (0.0, 100)	5	$$score\left(x\right)=\left\{\begin{array}{rl}\frac{C\times x}{A}& if \ x\le A\\ \left(\frac{C\times 100}{(100-A)}\right)-\left(\frac{C\times x}{(100-A)}\right)& if\ x> A\end{array}\right.$$ For a given ratio component, *x* is the percentage of calories consumed, A is the optimal recommended value, and C is the maximum possible score
Red and orange vegetables ratio	38.5 (0.0, 100)	38.5 (0.0, 100)	5	
Whole cereals ratio	75.0 (0.0, 100)	75.0 (0.0, 100)	10	
**Optimum components**				
Cereals	30.0 (0.0, 60.0)	30.0 (0.0, 60.0)	10	$$score\left(x\right)=\left\{\begin{array}{rlr}\frac{10\times x}{A}&if\ x\le A\\ \left(\frac{10\times B}{B-A}\right)-\left(\frac{10\times x}{(B-A)}\right)&if\ A < x < B\\ 0&if\ x\ge B\end{array}\right.$$ For a given optimum component, *x* is the percentage of calories consumed, A is the optimal recommended value, and B is the upper limit of the recommended range
Tubers and potatoes	1.6 (0.0, 3.1)	1.6 (0.0, 3.1)	10	
Dairy products	12.2 (0.0, 24.4)	6.1 (0, 12.2)	10	
Eggs and white meats	6.2 (0.0, 12.2)	6.2 (0.0, 12.2)	10	
Vegetable oils	14.1 (0.0, 28.3)	14.1 (0.0, 28.3)	10	
**Moderation components**				
Palm oil	0.0 (0.0, 2.4)	0.0 (0.0, 2.4)	10	$$score\left(x\right)=\left\{\begin{array}{rlr}10&if\,|x=0\\ 10- \left(\frac{10}{B\times x}\right)&if\,|0<x<B\\ 0&if\,|x\ge B\end{array}\right.$$ For a given moderation component, *x* is the percentage of calories consumed, and B is the upper limit of the recommended range
Red meats	0.0 (0.0, 2.4)	0.0 (0.0, 2.4)	10	
Animal fats	0.0 (0.0, 1.4)	0.0 (0.0, 1.4)	10	
Added sugars	0.0 (0.0, 4.8)	0.0 (0.0, 4.8)	10	
**Total score**			**150**	

^a^All values were calculated based on the EAT-Lancet diet [5] and modifications were made to meet the nutritional requirements of children and adolescents [38, 40]

^b^Most values are expressed as percentages of total caloric intake, except for the dark green vegetables and the red and orange vegetables ratio components which are expressed as percentage of total calories from vegetables, and the whole cereals ratio which is expressed as percentage of total calories from cereals

We calculated the PHDI scores for each sample diet following the methods described by Cacau et al. [19] and noted that scores were particularly low for animal-based components (see Table 1 and Supplemental Tables 1–5). Hence, the following adaptations were made to the original PHDI: Firstly, to accommodate children’s iron and vitamin D requirements, we created a single index component for animal-based protein sources that the original index had as separate components: *eggs*, *chicken & other poultry*, and *fish & seafood* [5]. The resulting component (*Eggs & white meats*) allowed a higher percentage of total caloric intake from *eggs & white meats* within a range of 0–12.2% of total calories, with an optimal value of 6.2%, which is equal to the sum of the mid caloric intakes proposed by the EAT-Lancet Commission for *eggs* (0.8%), *chicken & other poultry* (2.5%), and *fish & seafood* (2.9%) [5]. The maximum cut-off value was defined using the same logic. Secondly, to accommodate children’s calcium and vitamin D requirements, we doubled the optimal recommended value for *dairy products* from 6.1% [19] to 12.2% of total calories, and increased the upper limit from 12.2% [19] to 24.4%. We did not do this for adolescents as they were able to meet their calcium requirements by consuming around 6% of total calories from *dairy products* (see Supplemental Tables 3–5). Thirdly, to ensure the index optimised bioavailability of micronutrients, particularly calcium and iron, we replaced the *whole cereals* adequacy component with an optimum component that accounts for all *cereals* (refined and whole), and a *whole cereals ratio* component to moderate phytate consumption [24]. The optimum component allows a percentage of total caloric intake from *cereals* within a range of 0–60% of total calories, with an optimal value of 30%. The *whole cereals ratio* component emphasizes the consumption of whole to refined cereals in a 3:1 ratio, as recommended by Beal et al. after analysing the micronutrient adequacy of the EAT-Lancet diet [24]. Finally, to differentiate *palm oil* from other *vegetable oils*, as originally proposed by the EAT-Lancet diet [5], we reduced the maximum percentage of total caloric intake from *vegetable oils* from 30.7% to 28.3% and established a maximum percentage of total caloric intake from *palm oil* of 2.4%. With these modifications, the adapted PHDI-C includes four adequacy components (*nuts & peanuts, legumes, fruits,* and *vegetables*), three ratio components (*dark green vegetables ratio, red and orange vegetables ratio,* and *whole cereals ratio*), five optimum components (*cereals, tubers & potatoes, dairy products, eggs & white meats,* and *vegetable oils*), and four moderation components (*palm oil, red meats, animal fats,* and *added sugars*) (Table 2). Examples of food items included in each component are described in Supplemental Table 6. We used Cacau et al.’s scoring system, where all index components can score between 0 to 10 points, except for the *dark green vegetables* and *red and orange vegetables ratio* components, which can score between 0 to 5 points (to avoid an overrating of the *vegetables* component from which these two ratio components derive), adding up to a maximum of 10 points [19]. Following this same rationale, the newly added *whole cereals* ratio component can score between 0 to 10 points. The total PHDI-C score can range from 0 to 150 points. The PHDI-C components, the recommended percentages of total caloric intake for children and adolescents, and the formulae to calculate each component score are provided in Table 2.

When comparing the scores obtained using the original and adapted versions of the index across the six sample diets, total PHDI-C scores were notably higher than total PHDI scores (see Table 1 and Supplemental Tables 1–5).

### Calculation of PHDI-C & PHDI scores

We developed a decision tree (Supplemental Fig. 2) and food disaggregation methodology (Supplemental Table 7) to guide the calculation of PHDI-C scores from dietary data. The decision tree (Supplemental Fig. 2) was used to distinguish between: a) food items where calories could be allocated into a single index component without needing disaggregation (e.g., minimally processed foods, culinary ingredients, and processed foods based on a single ingredient plus food additives such as candies, processed meats, and soft drinks); b) composite foods where calories could be allocated into multiple index components and, therefore, needed to be disaggregated into ingredients (e.g., breakfast cereals, cookies, baked products, flavoured milks and yoghurts); and c) food items where calories could not be allocated into an index component (i.e., tea, coffee, cocoa powder, baking powder, baking soda, yeast, salt, herbs and spices). Calories from non-composite foods were allocated directly into each index component as per described in Supplemental Table 6. Then, the food disaggregation methodology (Supplemental Table 7) was used to guide the allocation of calories from composite foods’ main energy sources into corresponding index components. This process was informed by the ingredient list and nutrition information panels of food items reported in children’s dietary recalls. When the ingredient list was not available, we used information from similar products or created approximate recipes based on standard household recipes provided by INTA (available from authors on request). For example, flavoured milks were decomposed into two main calorie sources: milk and sugar. To estimate the caloric contribution of added sugars, we followed the Pan American Health Organization (PAHO) method for estimating free sugars which assumes that 50% of total sugars are intrinsic to milk (i.e., lactose) and 50% are added (i.e., “monosaccharides and disaccharides added to foods and beverages by the manufacturer, cook, and/or consumer plus sugars that are naturally present in honey, syrups and juices”) [41]. Therefore, of the total amount of sugars declared in flavoured milks, we allocated 50% into the *added sugars* component. All remaining calories, after discounting calories from added sugars, were allocated into the *dairy products* component. The food disaggregation process was conducted by a trained dietitian (CVH), who recorded all decisions and assumptions made. These decisions were then discussed with a panel of expert dietitians from INTA (*n* = 4) until agreement on assumptions/decisions was reached. The food disaggregation methodology, rationale and assumptions are described in detail in Supplemental Table 7.

After allocating calories from reported food items into the corresponding index components, we calculated the percentage of calories consumed from each index component relative to the total non-alcoholic caloric intake reported by each child. These percentages were then assessed against the recommended percentages of total caloric intake established for each PHDI-C component and scores were calculated using the formulae described in Table 2.

A similar process was followed to calculate each PHDI component score as per described by Cacau et al. [19]. Individual component scores were then added to obtain PHDI and PHDI-C total scores.

### Statistical analysis

We used descriptive statistics to report the PHDI & PHDI-C scores obtained by FECHiC participants. Linear regression models adjusted by child’s gender and age were fitted to explore whether the mean total PHDI-C score changed in expected directions according to dietary recall characteristics (e.g., higher scores when children reported having a special diet compared to a normal diet) and the nutritional composition of children’s diet (e.g., lower scores associated with higher consumption of added sugars or animal-based proteins). We reported adjusted estimates alongside 95% confidence intervals (CI). All statistical analyses were conducted in Stata v17.

## Results

### Sample description, dietary recall characteristics, and nutritional composition of children's diets

Fifty-two percent (*n* = 496) of FECHiC participants were female and more than 70% (*n* = 695) were 3–4 years of age (Table 3). Most dietary recalls were collected on a weekday (*n* = 821, 86%) and were reported by primary caretakers as children’s typical eating pattern (*n* = 801, 84%). Ninety five percent (*n* = 905) of children reported having a normal diet on the day of the dietary recall. Six percent (*n* = 54) of recalls were deemed to be unreliable by INTA’s dietetics team because of missing information on the amount of food consumed on certain meal occasions*.* On average, FECHiC participants consumed 1,181 kcal/day with 57% of their calories coming from carbohydrates, 29% from fats, and 14% from proteins. Total sugars contributed to 29% of total calories and consumption of fibre was low (7.2 g/1000 kcal).

**Table 3:** FECHiC participants’ characteristics, dietary recall characteristics, and nutritional composition of children’s diets (*n* = 958)

**Child characteristics**	**n**	**(%)**
**Gender**		
Male	462	(48.2)
Female	496	(51.8)
**Age**		
3–4 years	695	(72.6)
5–6 years	263	(27.4)
**Dietary recall characteristics**	**n**	**(%)**
**Day of the dietary recall**		
Weekday	821	(85.7)
Weekend day/holiday	137	(14.3)
**Type of eating pattern on the day of the dietary recall **^**a**^		
Typical	801	(83.6)
Atypical	157	(16.4)
**Type of diet on the day of the dietary recall **^**b**^		
Normal	905	(94.5)
Special	53	(5.5)
**Reliability of the dietary recall **^**c**^		
Reliable	904	(94.4)
Unreliable	54	(5.6)
**Diet nutritional composition**	**Mean**	**(SD)**
**Energy**		
Total energy intake, kcal/day	1181.4	(376.8)
**Macronutrients**		
Total protein intake, % total energy	14.1	(3.7)
Animal-based protein intake, % total energy	9.8	(3.8)
Plant-based proteins intake, % total energy	4.3	(2.6)
Total fats intake, % total energy	28.7	(6.6)
Saturated fats intake, % total energy	10.5	(3.4)
Trans fats intake, % total energy	0.5	(0.3)
Total carbohydrates intake, % total energy	57.7	(7.6)
Total sugars intake, % total energy	29.1	(8.7)
Total fibre intake, g/1000 kcal	7.2	(4.7)

*Abbreviations: FECHiC* Food Environment chilean cohort

^a^Typical eating pattern refers to a recall from a regular day; typical eating pattern refers to a recall from a special occasion such celebration, vacation, or sickness

^b^Normal diet refers to an omnivorous diet with no dietary restriction of any kind; special diet refers to lactose free, gluten free, vegetarian, or vegan diets

^c^Unreliable recalls refer to recalls where there was missing information on the amount consumed of some food items

### Allocation of calories into corresponding index components and calculation of scores

**Fig. 1:**
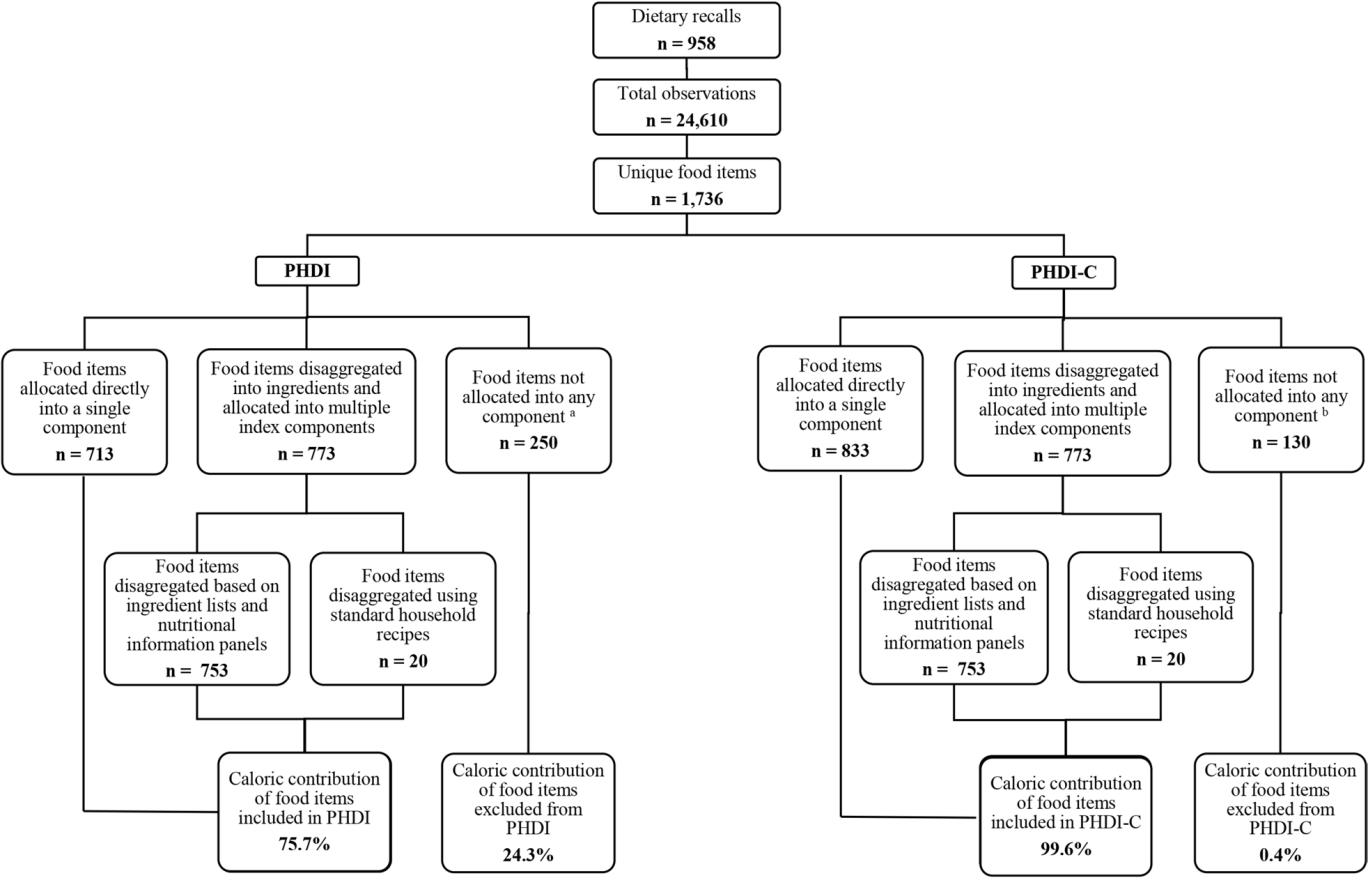
Application of the decision tree and food disaggregation methodology to allocate calories from reported food items into corresponding index components. Abbreviations: PHDI, Planetary Health Diet Index; PHDI-C, Planetary Health Diet Index for children and adolescents. **a** Includes refined cereals, cocoa powder, baking powder, baking soda, yeast, salt, herbs and spices, artificially sweetened beverages, tea, coffee, water, and alcoholic beverages (i.e., wine used in culinary preparations). **b** Includes cocoa powder, baking powder, baking soda, yeast, salt, herbs and spices, artificially sweetened beverages, tea, coffee, water, and alcoholic beverages (i.e., wine used in culinary preparations)

From 958 dietary recalls and 24,610 observations reported by the sample of Chilean pre-schoolers, we identified 1,736 unique food items (Fig. 1). Calories from 713 (41.0%) and 833 (48.0%) unique food items were allocated into single components of the PHDI and the PHDI-C, respectively. Seven hundred seventy-three unique food items (44.5%) were disaggregated into ingredients and allocated into multiple index components. Of these, 97.4% (*n* = 753) were disaggregated based on the ingredient list and nutritional information panels declared by manufacturers, and the rest (*n* = 20, 2.6%) were disaggregated using standard household recipes. Food items included in the PHDI accounted for 75.7% of total calories, whereas food items included in the PHDI-C accounted for 99.6%. This difference was due to the inclusion of refined cereals within the PHDI-C components, which led to a smaller number of excluded food items (*n* = 130 vs *n* = 250) and a lower percentage of calories excluded (0.4% vs 24.3%).

### Comparison of PHDI & PHDI-C scores

FECHiC participants’ total PHDI and PHCI-C scores were low, indicating low adherence to sustainable healthy diets. Mean total PHDI score was 40.7 (12.1) out of 150 points, with a minimum and maximum score of 3.1 and 80.0 points, respectively (Table 4). In contrast, mean total PHDI-C score was 50.1 (14.6) out of 150 points, with a minimum and maximum score of 9.6 and 97.6 points, respectively.

**Table 4:** Comparison of PHDI & PHDI-C scores among FECHiC participants (*n* = 958)

PHDI Components ^a^	Possible scores	Participants’ PHDI scores ^b^				PHDI-C Components ^c^	Possible scores	Participants’ PHDI-C scores ^d^			
		**Mean (SD)**	**Median (IQR)**	**Min**	**Max**			**Mean (SD)**	**Median (IQR)**	**Min**	**Max**
**Adequacy components**						**Adequacy components**					
Nuts & peanuts	0—10	0.2 (1.1)	0.0 (0.0 – 0.0)	0.0	10.0	Nuts & peanuts	0—10	0.2 (1.1)	0.0 (0.0 – 0.0)	0.0	10.0
Legumes	0 – 10	1.6 (3.2)	0.0 (0.0 – 0.6)	0.0	10.0	Legumes	0 – 10	1.6 (3.2)	0.0 (0.0 – 0.6)	0.0	10.0
Whole cereals	0 – 10	0.3 (0.9)	0.0 (0.0 – 0.1)	0.0	7.9						
Fruits	0 – 10	6.0 (4.2)	7.2 (0.8 – 10.0)	0.0	10.0	Fruits	0 – 10	6.0 (4.2)	7.2 (0.8 – 10.0)	0.0	10.0
Vegetables	0 – 10	4.1 (3.2)	3.6 (1.6 – 6.4)	0.0	10.0	Vegetables	0 – 10	4.1 (3.2)	3.6 (1.6 – 6.4)	0.0	10.0
**Ratio components**						**Ratio components**					
DGV ratio	0—5	0.4 (1.0)	0.0 (0.0 – 0.0)	0.0	5.0	DGV ratio	0—5	0.4 (1.0)	0.0 (0.0 – 0.0)	0.0	5.0
ReV ratio	0—5	2.4 (1.8)	2.7 (0.0 – 4.0)	0.0	5.0	ReV ratio	0—5	2.4 (1.8)	2.7 (0.0 – 4.0)	0.0	5.0
						Whole cereals ratio	0—10	0.6 (1.5)	0.0 (0.0 – 0.1)	0.0	9.9
**Optimum components**						**Optimum components**					
						Cereals	0—10	7.0 (2.2)	7.5 (5.7 – 8.8)	0.0	10.0
Tubers & potatoes	0 – 10	0.5 (1.7)	0.0 (0.0 – 0.0)	0.0	9.9	Tubers & potatoes	0 – 10	0.5 (1.7)	0.0 (0.0 – 0.0)	0.0	9.9
Dairy products	0 – 10	1.1 (2.5)	0.0 (0.0 – 0.0)	0.0	9.9	Dairy products	0 – 10	3.8 (3.4)	3.6 (0.0 – 7.0)	0.0	10.0
Eggs	0 – 10	0.5 (1.7)	0.0 (0.0 – 0.0)	0.0	10.0	Eggs & white meats	0 – 10	2.8 (3.4)	0.7 (0.0 – 5.8)	0.0	10.0
Fish & seafood	0 – 10	0.2 (1.0)	0.0 (0.0 – 0.0)	0.0	10.0						
Vegetable oils	0 – 10	6.2 (2.3)	6.6 (4.4 – 8.5)	0.0	10.0	Vegetable oils	0 – 10	5.6 (2.8)	5.8 (3.5 – 7.9)	0.0	10.0
**Moderation components**						**Moderation components**					
Chicken & other poultry	0 – 10	6.8 (4.3)	10.0 (1.3 – 10.0)	0.0	10.0						
						Palm oil	0 – 10	4.7 (4.6)	4.4 (0.0 – 10.0)	0.0	10.0
Red meats	0 – 10	4.9 (4.9)	3.2 (0.0 – 10.0)	0.0	10.0	Red meats	0 – 10	4.9 (4.9)	3.2 (0.0 – 10.0)	0.0	10.0
Animal fats	0 – 10	5.4 (4.8)	10.0 (0.0 – 10.0)	0.0	10.0	Animal fats	0 – 10	5.4 (4.8)	10.0 (0.0 – 10.0)	0.0	10.0
Added sugars	0 – 10	0.2 (1.0)	0.0 (0.0 – 0.0)	0.0	10.0	Added sugars	0 – 10	0.2 (1.0)	0.0 (0.0 – 0.0)	0.0	10.0
**TOTAL**	**0—150**	**40.7 (12.1)**	**40.5 (32.4 – 49.0)**	**3.1**	**80.0**	**TOTAL**	**0—150**	**50.1 (14.6)**	**50.0 (39.5 – 59.8)**	**9.6**	**97.6**

*Abbreviations: PHDI* Planetary health diet index, *PHDI-C* Planetary health diet index for children and adolescents, *FECHiC* Food environment chilean cohort, *DGV* Dark green vegetables, *ReV* Red and orange vegetables, *SD* Standard deviation, *IQR* Interquartile range, *Min* Minimum, *Max* Maximum

^a^Examples of food items included in each component are described in Cacau et al.’s supplemental Table 1 [19]

^b^PHDI scores were calculated as described by Cacau et al. [19]

^c^Examples of food items included in each component are described in Supplemental Table 6

^d^PHDI-C scores were calculated as described in Table 2

Individual component scores were very low for *nuts & peanuts*, *legumes*, *dark green vegetables*, *whole cereals*, *tubers & potatoes*, and *added sugars* across both indices, with at least three quarters of the sample scoring less than 1 point (Table 4). Furthermore, when diets were scored using the original PHDI, at least three-quarters of the sample scored 0 points for the *dairy products, eggs,* and *fish & seafood* components, whereas when diets were scored using the PHDI-C, the median scores for the *dairy products* and *eggs & white meats* components were 3.6 (IQR 0.0 – 7.0) and 0.7 (IQR 0.0 – 5.8) points, respectively. Regardless of the index applied, the only component where at least half of the sample obtained 10 points was *animal fats*.

### Associations between total PHDI-C score, dietary recall characteristics and nutritional composition of children’s diets

We observed that the mean total PHDI-C score was significantly lower on weekends compared to weekdays (47.52 [95%CI 45.08, 49.95] vs 50.49 [95%CI 49.50, 51.49]) and when the type of eating pattern on the day of the dietary recall was reported as atypical (because of celebration, sickness, or vacation) compared to typical (47.14 [95%CI 44.86, 49.41] vs 50.64 (95%CI 49.63, 51.65]) (Table 5). Mean total PHDI-C score was significantly higher when children reported having a special diet (e.g., vegetarian diet) compared to a normal diet (i.e., omnivorous) (53.96 [95%CI 50.04, 57.88] vs 49.84 [95%CI 48.89, 50.79]). Moreover, total PHDI-C score was positively associated with total protein, plant-based protein, total carbohydrates, and total fibre intake, and negatively associated with animal-based protein, total fat, saturated fats, trans fats, and total sugars intake. These associations remained statistically significant after adjusting for child’s gender and age.

**Table 5:** Associations between total PHDI-C score, dietary recall characteristics, and nutritional composition of children’s diets^a^

	**TOTAL PHDI-C SCORE **^**b**^					
	**Unadjusted estimates **^**c**^			**Adjusted estimates **^**d**^		
	**Mean (95% CI)**	**Diff (95% CI)**	*P*-value	**Mean (95% CI)**	**Diff (95% CI)**	***P*** **-value**
**Dietary recall characteristics**						
**Day of the dietary recall**						
Weekday	50.49 (49.50, 51.49)			50.51 (49.51, 51.50)		
Weekend/holiday	47.52 (45.08, 49.95)	-2.97 (-5.61, -0.34)	0.027*	47.42 (44.97, 49.87)	-3.09 (-5.73, -0.44)	0.022*
**Type of eating pattern **^**e**^						
Typical	50.64 (49.63, 51.65)			50.65 (49.65, 51.66)		
Atypical	47.14 (44.86, 49.41)	-3.50 (-5.99, -1.02)	0.006*	47.08 (44.80, 49.36)	-3.57 (-6.06, -1.08)	0.005*
**Type of diet **^**f**^						
Normal	49.84 (48.89, 50.79)			49.84 (48.89, 50.79)		
Special	53.96 (50.04, 57.88)	4.12 (0.09, 8.15)	0.045*	53.98 (50.06, 57.90)	4.14 (0.11, 8.18)	0.044*
**Reliability of the dietary recall **^**g**^						
Reliable	50.26 (49.31, 51.21)			50.27 (49.31, 51.22)		
Unreliable	46.82 (42.94, 50.71)	-3.44 (-7.44, 0.56)	0.092	46.72 (42.83, 50.60)	-3.55 (-7.55, 0.45)	0.082
**Diet nutritional composition**						
**Energy**						
Total energy intake, kcal/day		-0.00 (-0.00, 0.00)	0.835		-0.00 (-0.0, 0.0)	0.733
**Macronutrients**						
Total protein intake, % total energy		0.44 (0.19, 0.69)	0.001*		0.44 (0.19, 0.69)	0.001*
Animal protein intake, % total energy		-0.46 (-0.69, -0.22)	< 0.001*		-0.45 (-0.69, -0.22)	< 0.001*
Plant protein intake, % total energy		1.94 (1.59, 2.28)	< 0.001*		1.94 (1.60, 2.28)	< 0.001*
Total fat intake, % total energy		-0.41 (-0.54, -0.27)	< 0.001*		-0.41 (-0.55, -0.27)	< 0.001*
Saturated fats intake, % total energy		-2.00 (-2.24, -1.76)	< 0.001*		-2.01 (-2.25, -1.76)	< 0.001*
Trans fats intake, % total energy		-11.03 (-14.12, -7.93)	< 0.001*		-11.12 (-14.23, -8.01)	< 0.001*
Carbohydrates intake, % total energy		0.29 (0.17, 0.41)	< 0.001*		0.29 (0.17, 0.41)	< 0.001*
Total sugars intake, % total energy		-0.14 (-0.24, -0.03)	0.012*		-0.14 (-0.24, -0.03)	0.011*
Total fibre intake, g/1000 kcal		1.49 (1.32, 1.66)	< 0.001*		1.49 (1.32, 1.66)	< 0.001*

*Abbreviations: PHDI-C* Planetary Health Diet Index for children and adolescents, *CI* Confidence interval, *diff* difference

^a^958 participants (4–6 years) from the Food Environment Chilean Cohort (FECHiC) were included in the analysis

^b^Total PHDI-C score range: 0–150 points

^c^Estimates and *p*-values from linear regressions models including one characteristic at a time

^d^Estimates and *p*-values from linear regression models including one characteristic at a time, adjusted for child’s gender (female vs male) and age (i.e., 3–4 years vs 5–6 years)

^e^Typical eating pattern refers to a recall from a regular day; atypical eating pattern refers to a recall from a special occasion such celebration, vacation, or sickness

^f^Normal diet refers to an omnivorous diet with no dietary restriction of any kind; special diet refers to lactose free, gluten free, vegetarian, or vegan diets

^g^Unreliable recalls refer to recalls where there is missing information on the amount consumed of some food items

^*^P-value < 0.05

## Discussion

We adapted the PHDI developed and validated by Cacau et al. [19] to better reflect children and adolescents’ micronutrient requirements. We piloted the adapted index among a sample of Chilean pre-schoolers and compared the scores obtained using the original and adapted versions of the index. Our results showed that the original PHDI accounted for 75% of children’s total caloric intake, whereas the adapted PHDI-C accounted for almost 100%. This was due to the inclusion of refined cereals within the PHDI-C components. Total PHDI & PHDI-C scores were low among this sample of Chilean pre-schoolers; however, the mean total score was lower when diets were scored using the PHDI compared to PHDI-C. Individual component scores were very low for *nuts & peanuts*, *legumes, dark green vegetables*, *whole cereals*, *tubers & potatoes,* and *added sugars* across both indices, and were particularly lower for the *dairy products*, *eggs*, and *fish & seafood* components when using the PHDI compared to the PHDI-C. Regardless of the index applied, *animal fats* was the only component where at least half of the sample obtained 10 points.

Differences observed in individual component scores between the indexes (e.g., higher scores for the *dairy products* and *eggs & white meats* components when the PHDI-C was used) are in line with the modifications we made to adapt the PHDI to better reflect children’s and adolescents’ nutritional requirements, including allowing a higher percentage of calories from the *dairy products* component and merging *eggs, fish & seafood,* and *chicken & other poultry* into a single index component (i.e., *eggs & white meats)*.

Our results also showed that the mean total PHDI-C score shifted in the expected direction with dietary recall characteristics (e.g., decreasing on weekends and special occasions, and increasing when children reported having a special diet such as a vegetarian diet), and with the nutritional composition of children’s diet (e.g., increasing with total protein, plant-based protein, total carbohydrates, and total fibre intake, and decreasing with total sugars, saturated fats, trans fats, and animal-based protein intake). However, a validation of the PHDI-C against gold standard measures of diet quality and diet-related environmental impact indicators is still required.

Our findings are consistent with previous studies conducted in children and adolescents [10, 23, 42]. Montejano et al. [10] assessed adherence to the EAT-Lancet diet among participants from the DONALD study (≥ 15 years of age) using a dietary index specifically developed for that purpose. They found that adherence to the EAT-Lancet diet was moderate, with the majority of participants obtaining more than 50% of the maximum score. The dietary index score was positively associated with plant-based protein and fibre intake, and negatively associated with added sugars, total and animal-based protein, and cholesterol intake. Marchioni et al. [23] assessed adherence to the PHDI among a representative sample of the Brazilian population (≥ 10 years of age) and found that adherence was low (45.9 [95%CI 45.6, 46.1] out of 150 points), with lowest scores observed for the *nuts & peanuts*, *whole cereals*, *dark green vegetables ratio*, *eggs*, *fish & seafood*, and *tubers & potatoes* components. Lastly, Bäck et al. [42] described how far Finnish pre-schoolers (3–6 years) were from meeting the targets set by the EAT-Lancet Commission using dietary targets calculated based on children’s mean dietary intake. They found that children’s consumption of *nuts*, *legumes*, *whole cereals*, *vegetables*, and *unsaturated oils* was lower than recommended, and that consumption of *added sugars*, *red meats*, *dairy products*, and *tubers & potatoes* were above the EAT-Lancet recommendation. Similar to our findings regarding *animal fats*, Bäck et al. reported that there was a high proportion of Finnish pre-schoolers who met the target for saturated fats [42]. This collective evidence highlights the large dietary gap between current diets and sustainable healthy diets and calls for triple-duty actions aimed at improving children's adherence to sustainable healthy diets for better human and planet health.

### Strengths and limitations

PHDI-C provides researchers with a nutritionally adequate tool for use in children and adolescents that allows comparison of current diets with sustainable healthy diets and provides a score that can be used to examine associations with a wide range of outcomes. The use of energy-adjusted cut-off values gives the PHDI-C the ability to account for age-specific energy and nutrient requirements, and the use of continuous scoring scales over dichotomous scales increases the index's discriminatory power [20].

An important contribution of this study is the development of a decision tree and food disaggregation methodology to guide the allocation of calories from reported food items into index components. Similar processes have been described in previous studies [8, 10, 12, 15, 19], but not in sufficient detail to replicate the methods, particularly when it comes to allocating calories from composite foods into multiple index components. Some studies allocated composite foods into a single index component, for example by allocating deep-fried potatoes into the ‘tubers or starchy vegetables’ component [8, 12], whereas others disaggregated composite foods into ingredients [10, 15, 19], for example by decomposing deep-fried potatoes into potatoes and vegetable oils, allocating each ingredient in the corresponding index component [19].

Among the three studies that disaggregated composite foods into ingredients [10, 15, 19], two provided a brief explanation on how to conduct this procedure on processed and ultra-processed products [10, 19]. Cacau et al. [19] created recipes based on Brazilian household standard recipes, whereas Montejano et al. [10, 43] created recipes based on the ingredients list and nutritional information panels of packaged products. Given the richness of our data, we were able to use the ingredients list and nutritional information panels to disaggregate 97.4% of composite products reported in our database, limiting the creation of recipes based on household standard recipes to less than 3% of products.

To counteract the lack of a replicable methodology explaining how to disaggregate processed and ultra-processed products into ingredients and allocate their calories into multiple index components [10, 15, 19], our study provides a detailed description of the assumptions and associated rationale guiding every decision. Assumptions were based on widely used methodologies, where possible, such as the PAHO method for estimating free sugars [41], and were supported by a group of expert dietitians. Because it does not rely on creating approximate recipes based on household standard recipes, it may be more useful for disaggregating ultra-processed products which contain ingredients created for industrial rather than household use [44]. Finally, along with the work of the EAT-Lancet Commission and others, this paper advances research on healthy diet metrics by including an explicit planetary component.

We have shown that the application of the PHDI-C is feasible among a sample of Chilean pre-schoolers; however, there are some limitations. Firstly, the availability of dietary data from a single 24-h recall limited our ability to provide a representative measure of children’s usual intake [45]. A better estimate of children’s usual intake could have been obtained if we had had access to a minimum of three 24-h multiple pass recalls conducted over weekdays and weekend days [45]; however, this information was not available. We also acknowledge that this type of dietary assessment method is susceptible to recall bias [46]; nonetheless, the involvement of trained dietitians in conducting the dietary recalls following the USDA multiple-pass method and using primary caretakers as proxy reporters likely contributed to minimizing the risk of recall bias [29, 45]. Secondly, the use of pre-existing data from a convenience sample of Chilean children aged 3–6 years means that the results reported in this study cannot be generalizable to the entire paediatric Chilean population. Furthermore, it restricted our ability to pilot the index applicability to preschool children only. While having data on school children and adolescents would have strengthened our analysis, we did not have access to this information. Future studies should be conducted among these age groups. Thirdly, the index application requires detailed dietary data, nutrient composition information and, ideally, the ingredients list for all reported packaged food items. Where sufficiently detailed information is not available, the methodology will lose precision and should be used with caution. Participating in the INFORMAS project might help countries obtain relevant information [35]. Fourthly, the use Chilean dietary data in this study means the decision-making process used to disaggregate composite foods and allocate calories into multiple index components may only be applicable to countries with a similar food supply to Chile. Nevertheless, the list of food items disaggregated was vast (more than 1,000 unique products), and countries could use the methodology to create approximate recipes after looking at ingredients lists for products manufactured locally. Fifthly, even though the PHDI-C is based on the PHDI, which has been previously validated against measures of diet quality and environmental sustainability among Brazilian adults, the adapted PHDI-C has yet to be validated for children and adolescents.

Our findings showed that the mean total PHDI-C score was lower on weekends and special occasions (e.g., holidays or celebrations), and was higher when mothers reported children had a special diet (e.g., vegetarian diet). Furthermore, the total PHDI-C score was positively associated with total protein, plant-based protein, total carbohydrates, and total fibre intake, and negatively associated with total sugars, saturated fats, trans fats, and animal-based protein intake. These results suggest that the adapted PHDI-C might be positively associated with diet quality, but an index validation is still required. Also, the lack of environmental impact data for Chile prevented us from exploring the association between the adapted PHDI-C and diet-related environmental impact indicators. However, given that the modifications introduced to create the PHDI-C did not surpass the maximum levels of energy intake recommended by the EAT-Lancet commission [5], it is likely that these associations remain for the PHDI-C. Although, a common limitation of indices developed based on the EAT-Lancet diet is that the EAT-Lancet Commission centred its recommendations on environmental impact indicators from life-cycle analyses of agricultural commodities [5]. Consequently, the PHDI-C may underestimate the overall diet-related environmental impact because it does not account for the environmental impact associated with processing, packaging, distributing, storing and preparing processed and ultra-processed foods [47]. Finally, the absence of micronutrient composition information on FECHiC participants’ dietary recalls prevented us from exploring associations between total PHDI-C score and micronutrient adequacy of children’s diets. This should be explored in future studies.

## Conclusions

The PHDI-C provides a replicable method for measuring adherence to sustainable healthy diets among children and adolescents that takes into account their specific nutritional needs. The use of this tool will enable researchers and decision-makers with access to children's dietary data to monitor in-country trends and cross-country differences in adherence to healthy, nutritious, and environmentally sustainable diets that can help guide the food system transformation required to improve child and planetary health [5, 48]. Furthermore, it can serve as a dietary metric to measure the effectiveness of triple-duty actions aimed at improving children's diets towards addressing the global syndemic of obesity, undernutrition, and climate change.

Future studies should evaluate the validity of the PHDI-C for measuring the quality, nutritional adequacy, and environmental sustainability of diets among children and adolescents of different cultures and age groups.

## Supplementary Information

**Additional file 1:Supplemental Figure 1.** Participant flow chart. **Supplemental Table 1.** Example of a sustainable healthy diet for a two-year-old girl with a caloric requirement of 1,047 kcal/day. **Supplemental Table 2.** Example of a sustainable healthy diet for a twelve-year-old boy with a caloric requirement of 2,548 kcal/day. **Supplemental Table 3.** Example of a sustainable healthy diet for a thirteen-year-old girl with a caloric requirement of 2,379 kcal/day. **Supplemental Table 4.** Example of a sustainable healthy diet for an eighteen-year-old boy with a caloric requirement of 3,410 kcal/day. **Supplemental Table 5.** Example of a sustainable healthy diet for an eighteen-year-old girl of reproductive age with a caloric requirement of 2,503 kcal/day. **Supplemental Figure 2.** Decision tree to guide the allocation of calories from reported food items into PHDI-C components. **Supplemental Table 6.** Food items included in each component of the Planetary Health Diet Index for children and adolescents (PHDI-C). **Supplemental Table 7.** Food disaggregation methodology for allocating calories from composite foods into multiple components of the Planetary Health Diet Index for children and adolescents (PHDI-C).

## Data Availability

The de-identified data described in the manuscript, code book, and analytic code are not publicly available but are available from the corresponding author on reasonable request. Proposals should be directed to the corresponding author, who will then pass the proposal on to members of the Center for Research in Food Environments and Prevention of Nutrition-related Chronic Diseases (CIAPEC)—INTA for deliberation and approval. To gain access, data requestors will need to sign a data access and collaboration agreement.

## Abbreviations

DRI: Dietary Reference Intakes
FAO: Food and Agriculture Organization
INTA: Institute of Nutrition and Food Technology
IOM: Institute of Medicine
PAHO: Pan American Health Organization
PHDI: Planetary Health Diet Index
PHDI-C: Planetary Health Diet Index for children and adolescents
USDA: United States Department of Agriculture
WHO: World Health Organization
CI: Confidence interval

## References

[1] World Health Organization. Malnutrition fact sheet: WHO; 2020 [updated 01/04/2020. Available from: https://www.who.int/news-room/fact-sheets/detail/malnutrition.

[2] Murray CJL, Aravkin AY, Zheng P, Abbafati C, Abbas KM, Abbasi-Kangevari M, et al. Global burden of 87 risk factors in 204 countries and territories, 1990–2019: a systematic analysis for the Global Burden of Disease Study 2019. The Lancet. 2020;396(10258):1223–49. 10.1016/S0140-6736(20)30752-2 PMC7566194 33069327

[3] Springmann M, Mason-D’Croz D, Robinson S, Garnett T, Godfray HC, Gollin D, et al. Global and regional health effects of future food production under climate change: a modelling study. Lancet (London, England). 2016;387(10031):1937–46. 26947322 10.1016/S0140-6736(15)01156-3

[4] Swinburn BA, Kraak VI, Allender S, Atkins VJ, Baker PI, Bogard JR, et al. The global syndemic of obesity, undernutrition, and climate change: the lancet commission report. The Lancet. 2019;393(10173):791–846. 10.1016/S0140-6736(18)32822-8 30700377

[5] Willett W, Rockström J, Loken B, Springmann M, Lang T, Vermeulen S, et al. Food in the Anthropocene: the EAT–Lancet commission on healthy diets from sustainable food systems. The Lancet. 2019;393(10170):447–92. 10.1016/S0140-6736(18)31788-4 30660336

[6] Food and Agriculture Organisation WHO. Sustainable healthy diets – Guiding principles. Rome: FAO and WHO; 2019.

[7] Forestell CA. Flavor Perception and Preference Development in Human Infants. Ann Nutr Metab. 2017;70(Suppl 3):17–25. 28903110 10.1159/000478759

[8] Knuppel A, Papier K, Key TJ, Travis RC. EAT-Lancet score and major health outcomes: the EPIC-Oxford study. Lancet. 2019;394(10194):213–4. 10.1016/S0140-6736(19)31236-X. 10.1016/S0140-6736(19)31236-X 31235280

[9] Bozeman JF, Springfield S, Theis TL. Meeting EAT-Lancet food consumption, nutritional, and environmental health standards: a U.S. case study across racial and ethnic subgroups. Environ Justice. 2020;13(5):160–72. 10.1089/env.2020.0018. 10.1089/env.2020.0018 PMC7580058 33101580

[10] Montejano Vallejo R, Schulz CA, van de Locht K, Oluwagbemigun K, Alexy U, Nöthlings U. Associations of adherence to a dietary index based on the EAT-Lancet reference diet with nutritional, anthropometric, and ecological sustainability parameters: results from the German DONALD cohort study. J Nutr. 2022;152(7):1763–72. 10.1093/jn/nxac094. 10.1093/jn/nxac094 PMC9258554 35554563

[11] López G, Batis C, González C, Chávez M, Cortés-Valencia A, López-Ridaura R, et al. EAT-Lancet Healthy Reference Diet score and diabetes incidence in a cohort of Mexican women. Eur J Clin Nutr. 2023;77(3):348–55. 36471166 10.1038/s41430-022-01246-8

[12] Stubbendorff A, Sonestedt E, Ramne S, Drake I, Hallström E, Ericson U. Development of an EAT-Lancet index and its relation to mortality in a Swedish population. Am J Clin Nutr. 2022;115(3):705–16. 10.1093/ajcn/nqab369. 10.1093/ajcn/nqab369 PMC8895215 34791011

[13] Ali Z, Scheelbeek PFD, Felix J, Jallow B, Palazzo A, Segnon AC, et al. Adherence to EAT-Lancet dietary recommendations for health and sustainability in the Gambia. Environ Res Lett. 2022;17(10):104043. 10.1088/1748-9326/ac9326. 10.1088/1748-9326/ac9326 PMC9536464 36238079

[14] Kesse-Guyot E, Rebouillat P, Brunin J, Langevin B, Allès B, Touvier M, et al. Environmental and nutritional analysis of the EAT-Lancet diet at the individual level: insights from the NutriNet-Santé study. J Clean Prod. 2021;296:126555.

[15] Trijsburg L, Talsma EF, Crispim SP, Garrett J, Kennedy G, de Vries JHM, et al. Method for the development of WISH, a globally applicable index for healthy diets from sustainable food systems. Nutrients. 2020;13(1):93. 10.3390/nu13010093. 10.3390/nu13010093 PMC7824146 33396659

[16] Colizzi C, Harbers MC, Vellinga RE, Verschuren WMM, Boer JMA, Biesbroek S, et al. Adherence to the EAT-Lancet healthy reference diet in relation to risk of cardiovascular events and environmental impact: results from the EPIC-NL cohort. J Am Heart Assoc. 2023;12(8):e026318. 10.1161/JAHA.122.026318. 10.1161/JAHA.122.026318 PMC10227249 37066787

[17] Campirano F, López-Olmedo N, Ramírez-Palacios P, Salmerón J. Sustainable dietary score: methodology for its assessment in Mexico based on EAT-Lancet recommendations. Nutrients. 2023;15(4):1017. 10.3390/nu15041017. 10.3390/nu15041017 PMC9967068 36839374

[18] Shamah-Levy T, Gaona-Pineda E, Mundo-Rosas V, Méndez Gómez-Humarán I, Rodríguez-Ramírez S. Association of a healthy and sustainable dietary index and overweight and obesity in Mexican adults. Salud Publica Mex. 2020;62(6):745–53. 33620971 10.21149/11829

[19] Cacau LT, De Carli E, de Carvalho AM, Lotufo PA, Moreno LA, Bensenor IM, et al. Development and validation of an index based on EAT-Lancet recommendations: the planetary health diet index. Nutrients. 2021;13(5):1698. 10.3390/nu13051698. 10.3390/nu13051698 PMC8156093 34067774

[20] Burggraf C, Teuber R, Brosig S, Meier T. Review of a priori dietary quality indices in relation to their construction criteria. Nutr Rev. 2018;76(10):747–64. 10.1093/nutrit/nuy027. 10.1093/nutrit/nuy027 PMC6130981 30053192

[21] Cacau LT, Bensenor IM, Goulart AC, Cardoso LO, Lotufo PA, Moreno LA, et al. Adherence to the planetary health diet index and obesity indicators in the Brazilian longitudinal study of adult health (ELSA-Brasil). Nutrients. 2021;13(11):3691. 10.3390/nu13113691. 10.3390/nu13113691 PMC8625681 34835947

[22] Cacau LT, Benseñor IM, Goulart AC, Cardoso LO, Santos IS, Lotufo PA, et al. Adherence to the EAT-Lancet sustainable reference diet and cardiometabolic risk profile: cross-sectional results from the ELSA-Brasil cohort study. Eur J Nutr. 2023;62(2):807–17. 10.1007/s00394-022-03032-5. 10.1007/s00394-022-03032-5 36266476

[23] Marchioni DM, Cacau LT, De Carli E, Carvalho AM, Rulli MC. Low adherence to the EAT-Lancet sustainable reference diet in the Brazilian population: findings from the national dietary survey 2017–2018. Nutrients. 2022;14(6):1187. 10.3390/nu14061187. 10.3390/nu14061187 PMC8956109 35334839

[24] Beal T, Ortenzi F, Fanzo J. Estimated micronutrient shortfalls of the EAT–Lancet planetary health diet. Lancet Planetary Health. 2023;7(3):E233–7. 36889864 10.1016/S2542-5196(23)00006-2

[25] Lassen AD, Christensen LM, Trolle E. Development of a Danish adapted healthy plant-based diet based on the EAT-Lancet reference diet. Nutrients. 2020;12(3):738. 10.3390/nu12030738. 10.3390/nu12030738 PMC7146415 32168838

[26] Corvalan C, Reyes M, Garmendia ML, Uauy R. Structural responses to the obesity and non‐communicable diseases epidemic: the Chilean law of food labeling and advertising. Obes Rev. 2013;14(Suppl 2):79–87. 10.1111/obr.12099 24102671

[27] Venegas Hargous C, Reyes M, Smith Taillie L, Gonzalez CG, Corvalan C. Consumption of non-nutritive sweeteners by pre-schoolers of the food and environment Chilean cohort (FECHIC) before the implementation of the Chilean food labelling and advertising law. Nutr J. 2020;19(1):69. 32650775 10.1186/s12937-020-00583-3 PMC7353755

[28] Raper N, Perloff B, Ingwersen L, Steinfeldt L, Anand J. An overview of USDA’s Dietary Intake Data System. J Food Compos Anal. 2004;17(3–4):545–55.

[29] Moshfegh AJ, Rhodes DG, Baer DJ, Murayi T, Clemens JC, Rumpler WV, et al. The US Department of Agriculture Automated Multiple-Pass Method reduces bias in the collection of energy intakes. Am J Clin Nutr. 2008;88(2):324–32. 18689367 10.1093/ajcn/88.2.324

[30] Universidad de Chile, Ministerio de Salud. Atlas fotográfico de alimentos y preparaciones típicas chilenas: encuesta nacional de consumo alimentario. Santiago. Chile: Ministerio de Salud; 2010.

[31] Quintiliano D, Jara M. Protocolo Sistema de Clasificación de los Alimentos – CEPOC. Santiago, Chile: Universidad de Chile; 2016.

[32] Rebolledo N, Reyes M, Popkin B, Adair L, Avery C, Corvalán C, et al. Changes in nonnutritive sweetener intake in a cohort of preschoolers after the implementation of Chile’s Law of Food Labelling and Advertising. Pediatr Obes. 2022;17(7):e12895. 35088571 10.1111/ijpo.12895

[33] United States Department of Agriculture (USDA), Agricultural Research Service. FoodData Central 2019 [Available from: https://ndb.nal.usda.gov/ndb/.

[34] Zacarías I, Barrios L, González C, Loeff T, Vera G. Tabla de Composición de Alimentos. Santiago, Chile: Instituto de Nutrición y Tecnología de los Alimentos; 2018.

[35] INFORMAS. Chile | INFORMAS 2020 Available from: https://www.informas.org/chile/.

[36] Kanter R, Reyes M, Corvalán C. Photographic Methods for Measuring Packaged Food and Beverage Products in Supermarkets. Current Developments in Nutrition. 2017;1(10):e001016-e. 29955678 10.3945/cdn.117.001016 PMC5998779

[37] FAO, WHO, UNU. Human energy requirements: Report of a Joint FAO/WHO/UNU Expert Consultation. Rome, 17–24 October 2001. Rome: FAO; 2004.

[38] Institute of Medicine. Dietary Reference Intakes: The Essential Guide to Nutrient Requirements. Washington, DC: IOM; 2006.

[39] Joint WHO/FAO/UNU Expert Consultation. Protein and Amino Acid Requirements in Human Nutrition. World Health Organ Tech Rep Ser. 2007;(935):1–265, back cover. PMID: 18330140. 18330140

[40] Ross A, Manson J, Abrams S, Aloia J, Brannon P, Clinton S, et al. The 2011 report on dietary reference intakes for calcium and vitamin D from the Institute of Medicine: what clinicians need to know. J Clin Endocrinol Metab. 2011;96(1):53–8. 21118827 10.1210/jc.2010-2704 PMC3046611

[41] Pan American Health Organization Nutrient Profile Model. Washington, DC: PAHO; 2016.

[42] Bäck S, Skaffari E, Vepsäläinen H, Lehto R, Lehto E, Nissinen K, et al. Sustainability analysis of Finnish pre-schoolers' diet based on targets of the EAT-Lancet reference diet. Eur J Nutr. 2022;61(2):717–28. 10.1007/s00394-021-02672-3. 10.1007/s00394-021-02672-3 PMC8854143 34524506

[43] Kroke A, Manz F, Kersting M, Remer T, Sichert-Hellert W, Alexy U, et al. The DONALD study History, current status and future perspectives. European J Nutrition. 2004;43(1):45–54. 14991269 10.1007/s00394-004-0445-7

[44] Monteiro CA, Cannon G, Moubarac JC, Levy RB, Louzada MLC, Jaime PC. The UN Decade of Nutrition, the NOVA food classification and the trouble with ultra-processing. Public Health Nutr. 2018;21(1):5–17. 28322183 10.1017/S1368980017000234 PMC10261019

[45] Burrows TL, Martin RJ, Collins CE. A systematic review of the validity of dietary assessment methods in children when compared with the method of doubly labeled water. J Am Diet Assoc. 2010;110(10):1501–10. 20869489 10.1016/j.jada.2010.07.008

[46] Naska A, Lagiou A, Lagiou P. Dietary assessment methods in epidemiological research: current state of the art and future prospects. F1000Research. 2017;6:926. 10.12688/f1000research.10703.1. 10.12688/f1000research.10703.1 PMC5482335 28690835

[47] Seferidi P, Scrinis G, Huybrechts I, Woods J, Vineis P, Millett C. The neglected environmental impacts of ultra-processed foods. Lancet Planetary Health. 2020;4(10):e437–8. 33038314 10.1016/S2542-5196(20)30177-7

[48] Fanzo J, Haddad L, Schneider KR, Béné C, Covic NM, Guarin A, et al. Viewpoint: Rigorous monitoring is necessary to guide food system transformation in the countdown to the 2030 global goals. Food Policy. 2021;104:102163.

[49] World Medical Association. World Medical Association Declaration of Helsinki: ethical principles for medical research involving human subjects. JAMA. 2013;310(20):2191–4. 10.1001/jama.2013.281053. 10.1001/jama.2013.281053 24141714

