# Correction: Adapting the planetary health diet index for children and adolescents

**DOI:** 10.1186/s12966-024-01690-8

**Published:** 2024-12-30

**Authors:** Carolina Venegas Hargous, Liliana Orellana, Claudia Strugnell, Camila Corvalan, Steven Allender, Colin Bell

**Affiliations:** 1 Global Centre for Preventive Health and Nutrition (GLOBE), Institute for Health Transformation, Deakin University, Geelong, Australia; 2 School of Medicine, Faculty of Health, Deakin University, Geelong, Australia; 3 Biostatistics Unit, Faculty of Health, Deakin University, Geelong, Australia; 4 Institute for Physical Activity and Nutrition (IPAN), Deakin University, Geelong, Australia; 5 Institute of Nutrition and Food Technology (INTA), University of Chile, Santiago, Chile

**Correction to: Venegas Hargous et al. Int J Behav Nutr Phys Act (2023) 20:146**

10.1186/s12966-023-01516-z.

Following the publication of the original article, a reader identified a typographical error in Table 2. The authors acknowledged the error and affirmed that the errors did not impact the calculations or the interpretation of the article’s results.

**Incorrect**:

Formulae to calculate Ratio components:

$$\:score\left(x\right)=\left\{\begin{array}{c}\frac{C\times\:x}{A}\:\:\:if\:x\le\:A\\\:\left(\frac{C\times\:100}{(100-A)}\right)-\left(\frac{C}{(100-A)\times\:x}\right)\:if\:x>A\end{array}\right.$$

For a given ratio component, *x* is the percentage of calories consumed, A is the optimal recommended value, and C is the maximum possible score.

Formulae to calculate Optimum components:

$$\:score\left(x\right)=\left\{\begin{array}{c}\frac{10\times\:x}{A}\:\:if\:\:x\le\:A\\\:\left(\frac{10\times\:B}{B-A}\right)-\left(\frac{10}{(B-A)\times\:x}\right)\:if\:\:A<x<B\\\:0\:\:if\:\:x\ge\:B\end{array}\right.$$

For a given optimum component, *x* is the percentage of calories consumed, A is the optimal recommended value, and B is the upper limit of the recommended range.

**Correct**:

Formulae to calculate Ratio components:

$$\:score\left(x\right)=\left\{\begin{array}{c}\frac{C\times\:x}{A}\:\:\:if\:x\le\:A\\\:\left(\frac{C\times\:100}{(100-A)}\right)-\left(\frac{C\times\:x}{(100-A)}\right)\:if\:x>A\end{array}\right.$$

For a given ratio component, *x* is the percentage of calories consumed, A is the optimal recommended value, and C is the maximum possible score.

Formulae to calculate Optimum components:

$$\:score\left(x\right)=\left\{\begin{array}{c}\frac{10\times\:x}{A}\:\:if\:\:x\le\:A\\\:\left(\frac{10\times\:B}{B-A}\right)-\left(\frac{10\:\times\:x}{(B-A)}\right)\:if\:\:A<x<B\\\:\:\:0\:\:if\:\:x\ge\:B\end{array}\right.$$

For a given optimum component, *x* is the percentage of calories consumed, A is the optimal recommended value, and B is the upper limit of the recommended range.

The Original Article has been corrected.

