# Supplementary material for: Adapting the Planetary Health Diet Index for children and adolescents
Source: Int J Behav Nutr Phys Act. 2023 Dec 14;20:146. doi: 10.1186/s12966-023-01516-z (PMC10722829; doi:10.1186/s12966-023-01516-z)
Supplement: Supplementary file 1 — Additional file 1:Supplemental Figure 1. Participant flow chart. Supplemental Table 1. Example of a sustainable healthy diet for a two-year-old girl with a caloric requirement of 1,047 kcal/day. Supplemental Table 2. Example of a sustainable healthy diet for a twelve-year-old boy with a caloric requirement of 2,548 kcal/day. Supplemental Table 3. Example of a sustainable healthy diet for a thirteen-year-old girl with a caloric requirement of 2,379 kcal/day. Supplemental Table 4. Example of a sustainable healthy diet for an eighteen-year-old boy with a caloric requirement of 3,410 kcal/day. Supplemental Table 5. Example of a sustainable healthy diet for an eighteen-year-old girl of reproductive age with a caloric requirement of 2,503 kcal/day. Supplemental Figure 2. Decision tree to guide the allocation of calories from reported food items into PHDI-C components. Supplemental Table 6. Food items included in each component of the Planetary Health Diet Index for children and adolescents (PHDI-C). Supplemental Table 7. Food disaggregation methodology for allocating calories from composite foods into multiple components of the Planetary Health Diet Index for children and adolescents (PHDI-C). [file 12966_2023_1516_MOESM1_ESM.docx]

**Electronic Supplementary Material**

**Table of contents**

[Supplemental figure 1. Participant flow chart. 2](#_Toc143787200)

[Supplemental table 1. Example of a sustainable healthy diet for a two-year-old girl with a caloric requirement of 1,047 kcal/day. 3](#_Toc143787201)

[Supplemental table 2. Example of a sustainable healthy diet for a twelve-year-old boy with a caloric requirement of 2,548 kcal/day. 5](#_Toc143787202)

[Supplemental table 3. Example of a sustainable healthy diet for a thirteen-year-old girl with a caloric requirement of 2,379 kcal/day. 7](#_Toc143787203)

[Supplemental table 4. Example of a sustainable healthy diet for an eighteen-year-old boy with a caloric requirement of 3,410 kcal/day. 9](#_Toc143787204)

[Supplemental table 5. Example of a sustainable healthy diet for an eighteen-year-old girl of reproductive age with a caloric requirement of 2,503 kcal/day. 11](#_Toc143787205)

[Supplemental figure 2. Decision tree to guide the allocation of calories from reported food items into PHDI-C components. 13](#_Toc143787206)

[Supplemental table 6. Food items included in each component of the Planetary Health Diet Index for children and adolescents (PHDI-C). 14](#_Toc143787207)

[Supplemental table 7. Food disaggregation methodology for allocating calories from composite foods into multiple components of the Planetary Health Diet Index for children and adolescents (PHDI-C). 15](#_Toc143787208)

[References 23](#_Toc143787209)

# Supplemental figure 1. Participant flow chart.

Participants recruited at baseline

(n = 961)

Analytic sample

(n = 958)

Excluded (n = 3)

Participants with dietary data unavailable (n = 3)

# Supplemental table 1. Example of a sustainable healthy diet for a two-year-old girl with a caloric requirement of 1,047 kcal/day.

| **Meal** | **Food item ^a^** | **USDA code ^b^** | **Consumed amount (g)** | **Energy (kcal)** | **Proteins (g)** | **Carbohydrates (g)** | **Fibre (g)** | **Lipids (g)** | **SAFAs (g)** | **MUFAs (g)** | **PUFAs (g)** | **Cholesterol (mg)** | **n-6 PUFAs (g)** | **n-3 PUFAs (g)** | **Vitamin A (mg)** | **Thiamine (mg)** | **Riboflavin (mg)** | **Niacin (mg)** | **Vitamin B6 (mg)** | **Vitamin B12 (mg)** | **Folate (mg)** | **Pantothenic acid (mg)** | **Vitamin C (mg)** | **Vitamin E (mg)** | **Calcium (mg)** | **Copper (mg)** | **Iron (mg)** | **Magnesium (mg)** | **Phosphorus (mg)** | **Potassium (mg)** | **Selenium (mg)** | **Sodium (mg)** | **Zinc (mg)** | **Vitamin D (mg)** | **Vitamin K (mg)** | **Percentage of total caloric intake (%)** |
| --- | --- | --- | --- | --- | --- | --- | --- | --- | --- | --- | --- | --- | --- | --- | --- | --- | --- | --- | --- | --- | --- | --- | --- | --- | --- | --- | --- | --- | --- | --- | --- | --- | --- | --- | --- | --- |
| **Breakfast** | Reduced fat milk | 1079 | **125** | 63 | 4 | 6 | 0 | 2 | 1 | 1 | 0 | 10 | 0 | 0 | 104 | 0 | 0 | 0 | 0 | 1 | 3 | 0 | 0 | 0 | 158 | 0 | 0 | 15 | 129 | 199 | 2 | 49 | 1 | 1 | 0 | 5.8 |
|  | Oats, cooked | 8121 | **60** | 43 | 2 | 7 | 1 | 1 | 0 | 0 | 0 | 0 | 0 | 0 | 0 | 0 | 0 | 0 | 0 | 0 | 4 | 0 | 0 | 0 | 5 | 0 | 1 | 16 | 46 | 42 | 3 | 2 | 1 | 0 | 0 | 4.0 |
|  | Strawberries | 9316 | **10** | 4 | 0 | 1 | 0 | 0 | 0 | 0 | 0 | 0 | 0 | 0 | 0 | 0 | 0 | 0 | 0 | 0 | 0 | 0 | 6 | 0 | 2 | 0 | 0 | 1 | 2 | 16 | 0 | 0 | 0 | 0 | 0 | 0.3 |
|  | Bananas | 9040 | **25** | 22 | 0 | 6 | 1 | 0 | 0 | 0 | 0 | 0 | 0 | 0 | 1 | 0 | 0 | 0 | 0 | 0 | 5 | 0 | 2 | 0 | 1 | 0 | 0 | 7 | 6 | 90 | 0 | 0 | 0 | 0 | 0 | 2.1 |
|  | Almond butter | 12195 | **16** | 103 | 3 | 3 | 2 | 8 | 1 | 6 | 2 | 0 | 2 | 0 | 0 | 0 | 0 | 1 | 0 | 0 | 7 | 0 | 0 | 3 | 42 | 0 | 1 | 43 | 81 | 119 | 0 | 0 | 1 | 0 | 0 | 9.6 |
|  | Chia seeds | 12006 | **10** | 49 | 2 | 4 | 3 | 3 | 0 | 0 | 2 | 0 | 1 | 2 | 0 | 0 | 0 | 1 |  | 0 | 5 |  | 0 | 0 | 63 | 0 | 1 | 34 | 41 | 41 | 6 | 2 | 0 |  |  | 4.5 |
| **Lunch** | Cabbage, raw | 11109 | **30** | 8 | 0 | 2 | 1 | 0 | 0 | 0 | 0 | 0 | 0 | 0 | 1 | 0 | 0 | 0 | 0 | 0 | 8 | 0 | 22 | 0 | 11 | 0 | 0 | 5 | 10 | 59 | 0 | 2 | 0 | 0 | 12 | 0.7 |
|  | Carrots, raw | 11124 | **20** | 8 | 0 | 2 | 1 | 0 | 0 | 0 | 0 | 0 | 0 | 0 | 167 | 0 | 0 | 0 | 0 | 0 | 4 | 0 | 1 | 0 | 7 | 0 | 0 | 2 | 7 | 64 | 0 | 14 | 0 | 0 | 0 | 0.8 |
|  | Olive oil | 4053 | **2** | 18 | 0 | 0 | 0 | 2 | 0 | 1 | 0 | 0 | 0 | 0 | 0 | 0 | 0 | 0 | 0 | 0 | 0 | 0 | 0 | 0 | 0 | 0 | 0 | 0 | 0 | 0 | 0 | 0 | 0 | 0 | 1 | 1.6 |
|  | Lentils, cooked | 16070 | **110** | 128 | 10 | 22 | 9 | 0 | 0 | 0 | 0 | 0 | 0 | 0 | 0 | 0 | 0 | 1 | 0 | 0 | 199 | 1 | 2 | 0 | 21 | 0 | 4 | 40 | 198 | 406 | 3 | 2 | 1 | 0 | 2 | 11.9 |
|  | Potatoes, cooked | 11367 | **20** | 17 | 0 | 4 | 0 | 0 | 0 | 0 | 0 | 0 | 0 | 0 | 0 | 0 | 0 | 0 | 0 | 0 | 2 | 0 | 1 | 0 | 2 | 0 | 0 | 4 | 8 | 66 | 0 | 1 | 0 | 0 | 0 | 1.6 |
|  | Onion, cooked | 11283 | **7.5** | 3 | 0 | 1 | 0 | 0 | 0 | 0 | 0 | 0 | 0 | 0 | 0 | 0 | 0 | 0 | 0 | 0 | 1 | 0 | 0 | 0 | 2 | 0 | 0 | 1 | 3 | 12 | 0 | 0 | 0 | 0 | 0 | 0.3 |
|  | Chard, cooked | 11148 | **10** | 2 | 0 | 0 | 0 | 0 | 0 | 0 | 0 | 0 | 0 | 0 | 31 | 0 | 0 | 0 | 0 | 0 | 1 | 0 | 2 | 0 | 6 | 0 | 0 | 9 | 3 | 55 | 0 | 18 | 0 | 0 | 33 | 0.2 |
|  | Vegetable oil | 4669 | **2** | 18 | 0 | 0 | 0 | 2 | 0 | 0 | 1 | 0 | 1 | 0 | 0 | 0 | 0 | 0 | 0 | 0 | 0 | 0 | 0 | 0 | 0 | 0 | 0 | 0 | 0 | 0 | 0 | 0 | 0 | 0 | 4 | 1.6 |
|  | Salt | 2047 | **0.75** | 0 | 0 | 0 | 0 | 0 | 0 | 0 | 0 | 0 | 0 | 0 | 0 | 0 | 0 | 0 | 0 | 0 | 0 | 0 | 0 | 0 | 0 | 0 | 0 | 0 | 0 | 0 | 0 | 290 | 0 |  | 0 | 0.0 |
|  | Kiwi fruit | 63126500 | **50** | 29 | 1 | 7 | 2 | 0 | 0 | 0 | 0 | 0 | 0 | 0 | 2 | 0 | 0 | 0 | 0 | 0 | 13 | 0 | 37 | 1 | 18 | 0 | 0 | 8 | 17 | 99 | 0 | 3 | 0 | 0 | 20 | 2.7 |
| **Afternoon tea** | Reduced fat milk | 1079 | **150** | 75 | 5 | 7 | 0 | 3 | 2 | 1 | 0 | 12 | 0 | 0 | 125 | 0 | 0 | 0 | 0 | 1 | 3 | 1 | 0 | 0 | 189 | 0 | 0 | 18 | 155 | 239 | 3 | 59 | 1 | 2 | 0 | 7.0 |
|  | Wholemeal bread | 18075 | **80** | 203 | 10 | 34 | 5 | 3 | 1 | 0 | 1 | 0 | 1 | 0 | 0 | 0 | 0 | 4 | 0 | 0 | 34 | 1 | 0 | 2 | 130 | 0 | 2 | 61 | 170 | 200 | 21 | 360 | 1 | 0 | 0 | 18.9 |
|  | Avocado | 9037 | **50** | 80 | 1 | 4 | 3 | 7 | 1 | 5 | 1 | 0 | 0 | 0 | 4 | 0 | 0 | 1 | 0 | 0 | 41 | 1 | 5 | 1 | 6 | 0 | 0 | 15 | 26 | 243 | 0 | 4 | 0 | 0 | 11 | 7.4 |
| **Dinner** | Broccoli, cooked | 11091 | **25** | 9 | 1 | 2 | 1 | 0 | 0 | 0 | 0 | 0 | 0 | 0 | 19 | 0 | 0 | 0 | 0 | 0 | 27 | 0 | 16 | 0 | 10 | 0 | 0 | 5 | 17 | 73 | 0 | 10 | 0 | 0 | 35 | 0.8 |
|  | Tomatoes | 11529 | **35** | 6 | 0 | 1 | 0 | 0 | 0 | 0 | 0 | 0 | 0 | 0 | 15 | 0 | 0 | 0 | 0 | 0 | 5 | 0 | 5 | 0 | 4 | 0 | 0 | 4 | 8 | 83 | 0 | 2 | 0 | 0 | 0 | 0.6 |
|  | Olive oil | 4053 | **2** | 18 | 0 | 0 | 0 | 2 | 0 | 1 | 0 | 0 | 0 | 0 | 0 | 0 | 0 | 0 | 0 | 0 | 0 | 0 | 0 | 0 | 0 | 0 | 0 | 0 | 0 | 0 | 0 | 0 | 0 | 0 | 1 | 1.6 |
|  | Salmon, cooked | 15212 | **50** | 77 | 12 | 0 | 0 | 3 | 0 | 1 | 0 | 28 | 0 | 0 | 21 | 0 | 0 | 5 | 0 | 2 | 3 | 1 | 0 | 0 | 4 | 0 | 0 | 16 | 157 | 220 | 19 | 45 | 0 | 7 | 0 | 7.1 |
|  | White rice, cooked | 169711 | **80** | 78 | 2 | 17 | 1 | 0 | 0 | 0 | 0 | 0 | 0 | 0 | 0 | 0 | 0 | 0 | 0 | 0 | 1 | 0 | 0 | 0 | 2 | 0 | 0 | 0 | 6 | 8 | 4 | 4 | 0 | 0 | 0 | 7.2 |
|  | Vegetable oil | 4669 | **2** | 18 | 0 | 0 | 0 | 2 | 0 | 0 | 1 | 0 | 1 | 0 | 0 | 0 | 0 | 0 | 0 | 0 | 0 | 0 | 0 | 0 | 0 | 0 | 0 | 0 | 0 | 0 | 0 | 0 | 0 | 0 | 4 | 1.6 |
|  | Salt | 2047 | **0.3** | 0 | 0 | 0 | 0 | 0 | 0 | 0 | 0 | 0 | 0 | 0 | 0 | 0 | 0 | 0 | 0 | 0 | 0 | 0 | 0 | 0 | 0 | 0 | 0 | 0 | 0 | 0 | 0 | 116 | 0 |  | 0 | 0.0 |
| **Total intake** | | | | **1076** | **53** | **131** | **29** | **40** | **8** | **17** | **11** | **50** | **7** | **3** | **488** | **1** | **1** | **14** | **1** | **4** | **363** | **4** | **101** | **9** | **681** | **1** | **9** | **303** | **1089** | **2332** | **62** | **982** | **7** | **10** | **123** | **100** |
| **Nutritional requirements ^c^** | | | | 1047 | 39 | 131 | 25 | 41 | 9 | 20 | 12 | 300 | 9 | 2 | 300 | 1 | 1 | 6 | 1 | 1 | 200 | 2 | 15 | 6 | 700 | 0 | 7 | 80 | 460 | 3000 | 20 | 1000 | 3 | 15 | 55 | - |
| **Percentage of adequacy (%)** | | | | 103 | 136 | 100 | 117 | 98 | 83 | 88 | 93 | 17 | 72 | 112 | 163 | 195 | 171 | 231 | 274 | 323 | 181 | 221 | 671 | 156 | 97 | 339 | 132 | 379 | 237 | 78 | 310 | 98 | 231 | 64 | 224 | - |
|  | | | | | | | | | | | | | | | | | | | | | | | | | | | | | | | | | | | | |
| **Components of the PHDI ^d^** | | | | **Nuts & peanuts** | | **Legumes** | | **Fruits** | | **Vegetables** | | **ReV ratio** | | **DGV ratio** | | **Whole cereals** | | **Tubers & potatoes** | | **Dairy products** | | **Eggs** | **Fish and seafood** | | **Chicken & substitutes** | | **Vegetable oils** | | **Red meats** | | **Animal fats** | | **Added sugars** | | **Total score**  **(points)** | |
| **Recommended percentages of total caloric intake ^d^**  **Optimal value (range)** | | | | 11.6  (0.0, 100) | | 11.3  (0.0, 100) | | 5.0  (0.0, 100) | | 3.1  (0.0, 100) | | 38.5  (0.0, 100) | | 29.5  (0.0, 100) | | 32.4  (0.0, 100) | | 1.6  (0.0, 3.1) | | 6.1  (0.0, 12.2) | | 0.8  (0.0, 1.5) | 1.6  (0.0, 5.7) | | 0.0  (0.0, 5.0) | | 16.5  (0.0, 30.7) | | 0.0  (0.0, 2.4) | | 0.0  (0.0, 1.4) | | 0.0  (0.0, 4.8) | | 150.0 | |
| **Actual percentages of total caloric intake** | | | | 14.1 | | 11.9 | | 5.1 | | 3.4 | | 40.2 | | 29.8 | | 22.9 | | 1.6 | | 12.8 | | 0.0 | 7.1 | | 0.0 | | 14.0 | | 0.0 | | 0.0 | | 0.0 | | - | |
| **PHDI score ^d^** | | | | 10.0 | | 10.0 | | 10.0 | | 10.0 | | 4.9 | | 5.0 | | 7.1 | | 10.0 | | 0.0 | | 0.0 | 0.0 | | 10.0 | | 8.5 | | 10.0 | | 10.0 | | 10.0 | | 115.4 | |
| **Components of the PHDI-C ^e^** | | | | **Nuts & peanuts** | | **Legumes** | | **Fruits** | | **Vegetables** | | **ReV ratio** | | **DGV ratio** | | **Cereals** | **WC ratio** | **Tubers & potatoes** | | **Dairy products** | | **Eggs and white meats** | | | | | **Vegetable oils** | **Palm oil** | **Red meats** | | **Animal fats** | | **Added sugars** | | **Total score**  **(points)** | |
| **Recommended percentages of total caloric intake ^e^**  **Optimal value (range)** | | | | 11.6  (0.0, 100) | | 11.3  (0.0, 100) | | 5.0  (0.0, 100) | | 3.1  (0.0, 100) | | 38.5  (0.0, 100) | | 29.5  (0.0, 100) | | 30.0 (0.0, 60.0) | 75.0 (0.0, 100) | 1.6  (0.0, 3.1) | | 12.2  (0.0, 24.4) | | 6.2  (0.0, 12.2) | | | | | 14.1  (0.0 28.3) | 0.0  (0.0, 2.4) | 0.0  (0.0, 2.4) | | 0.0  (0.0, 1.4) | | 0.0  (0.0, 4.8) | | 150.0 | |
| **Actual percentages of total caloric intake** | | | | 14.1 | | 11.9 | | 5.1 | | 3.4 | | 40.2 | | 29.8 | | 30.1 | 76.0 | 1.6 | | 12.8 | | 7.1 | | | | | 14.0 | 0.0 | 0.0 | | 0.0 | | 0.0 | | - | |
| **PHDI-C score ^e^** | | | | 10.0 | | 10.0 | | 10.0 | | 10.0 | | 4.9 | | 5.0 | | 10.0 | 9.6 | 10.0 | | 9.5 | | 8.5 | | | | | 9.9 | 10.0 | 10.0 | | 10.0 | | 10.0 | | 147.4 | |
| Abbreviations: USDA, United States Department of Agriculture; SAFA, saturated fatty acids; MUFAs, Monounsaturated fatty acids; PUFAs, Polyunsaturated fatty acids; PHDI, Planetary Health Diet Index; PHDI-C, Planetary Health Diet Index for children and adolescents; ReV ratio, red and orange vegetables ratio; DGV ratio, dark green vegetables ratio; WC ratio; whole cereals ratio.  ^a^ Each food item is color-coded with its corresponding index component.  ^b^ Food items' nutritional composition was obtained from the USDA National Nutrient Database (1).  ^c^ The caloric requirement of 1,047 kcal/day corresponds to the caloric requirement of a 13.0 kg girl aged 2 years whose level of physical activity is moderate to high (2). Macronutrient requirements were calculated based on acceptable macronutrient distribution ranges (3). Micronutrient requirements were defined based on Recommended Dietary Allowances or Average Intakes for children aged 1-3-year-old (3, 4).  ^d^ Components and scores correspond to the PHDI developed and validated by Cacau et al (5). Each component is associated to a recommended range of total caloric intake expressed as percentage of total calories, except for the ratio components which are expressed as percentage of total calories from vegetables. All components can score between 0 to 10 points, except for the ratio components which can score between 0 to 5 points, resulting in a total score of 150 points (5).  ^e^ Components and scores correspond to the PHDI-C proposed in this study. Each component is associated to a recommended range of total caloric intake expressed as percentage of total calories, except for the DGV ratio and ReV ratio components which are expressed as percentage of total calories from vegetables, and the WC ratio which is expressed as percentage of total calories from cereals. All components can score between 0 to 10 points, except for the DGV and ReV ratio components which can score between 0 to 5 points, resulting in a total score of 150 points. The formula to calculate the score for each component is provided in Table 2. | | | | | | | | | | | | | | | | | | | | | | | | | | | | | | | | | | | | |

# Supplemental table 2. Example of a sustainable healthy diet for a twelve-year-old boy with a caloric requirement of 2,548 kcal/day.

| **Meal** | **Food item ^a^** | **USDA code ^b^** | **Consumed amount (g)** | **Energy (kcal)** | **Proteins (g)** | **Carbohydrates (g)** | **Fibre (g)** | **Lipids (g)** | **SAFAs (g)** | **MUFAs (g)** | **PUFAs (g)** | **Cholesterol (mg)** | **n-6 PUFAs (g)** | **n-3 PUFAs (g)** | **Vitamin A (mg)** | **Thiamine (mg)** | **Riboflavin (mg)** | **Niacin (mg)** | **Vitamin B6 (mg)** | **Vitamin B12 (mg)** | **Folate (mg)** | **Pantothenic acid (mg)** | **Vitamin C (mg)** | **Vitamin E (mg)** | **Calcium (mg)** | **Copper (mg)** | **Iron (mg)** | **Magnesium (mg)** | **Phosphorus (mg)** | **Potassium (mg)** | **Selenium (mg)** | **Sodium (mg)** | **Zinc (mg)** | **Vitamin D (mg)** | **Vitamin K (mg)** | **Percentage of total caloric intake (%)** |
| --- | --- | --- | --- | --- | --- | --- | --- | --- | --- | --- | --- | --- | --- | --- | --- | --- | --- | --- | --- | --- | --- | --- | --- | --- | --- | --- | --- | --- | --- | --- | --- | --- | --- | --- | --- | --- |
| **Breakfast** | Reduced fat milk | 1079 | **300** | 150 | 10 | 15 | 0 | 6 | 3 | 1 | 0 | 24 | 0 | 0 | 249 | 0 | 0 | 0 | 0 | 2 | 6 | 1 | 0 | 0 | 378 | 0 | 0 | 36 | 309 | 477 | 5 | 117 | 1 | 3 | 0 | 5.9 |
|  | Oats, cooked | 8121 | **150** | 107 | 4 | 18 | 3 | 2 | 0 | 1 | 1 | 0 | 1 | 0 | 0 | 0 | 0 | 0 | 0 | 0 | 9 | 0 | 0 | 0 | 14 | 0 | 1 | 41 | 116 | 105 | 8 | 6 | 2 | 0 | 0 | 4.2 |
|  | Strawberries | 9316 | **100** | 36 | 1 | 8 | 3 | 0 | 0 | 0 | 0 | 0 | 0 | 0 | 0 | 0 | 0 | 0 | 0 | 0 | 0 | 0 | 60 | 0 | 17 | 0 | 0 | 13 | 23 | 161 | 0 | 1 | 0 | 0 | 0 | 1.4 |
|  | Bananas | 9040 | **60** | 53 | 1 | 14 | 2 | 0 | 0 | 0 | 0 | 0 | 0 | 0 | 2 | 0 | 0 | 0 | 0 | 0 | 12 | 0 | 5 | 0 | 3 | 0 | 0 | 16 | 13 | 215 | 1 | 1 | 0 | 0 | 0 | 2.1 |
|  | Walnuts | 12154 | **20** | 124 | 5 | 2 | 1 | 12 | 1 | 3 | 7 | 0 | 7 | 1 | 0 | 0 | 0 | 0 | 0 | 0 | 6 | 0 | 0 | 0 | 12 | 0 | 1 | 40 | 103 | 105 | 3 | 0 | 1 | 0 | 1 | 4.8 |
|  | Chia seeds | 12006 | **20** | 97 | 3 | 8 | 7 | 6 | 1 | 0 | 5 | 0 | 1 | 4 | 0 | 0 | 0 | 2 |  | 0 | 10 |  | 0 | 0 | 126 | 0 | 2 | 67 | 81 | 81 | 11 | 3 | 1 |  |  | 3.8 |
| **Snack** | Almonds | 12061 | **25** | 145 | 5 | 5 | 3 | 12 | 1 | 8 | 3 | 0 | 3 | 0 | 0 | 0 | 0 | 1 | 0 | 0 | 11 | 0 | 0 | 6 | 67 | 0 | 1 | 68 | 120 | 183 | 1 | 0 | 1 | 0 | 0 | 5.7 |
| **Lunch** | Cabbage, raw | 11109 | **80** | 20 | 1 | 5 | 2 | 0 | 0 | 0 | 0 | 0 | 0 | 0 | 3 | 0 | 0 | 0 | 0 | 0 | 21 | 0 | 60 | 1 | 28 | 0 | 0 | 13 | 27 | 158 | 0 | 4 | 0 | 0 | 32 | 0.8 |
|  | Carrots, raw | 11124 | **30** | 12 | 0 | 3 | 1 | 0 | 0 | 0 | 0 | 0 | 0 | 0 | 251 | 0 | 0 | 0 | 0 | 0 | 6 | 0 | 2 | 0 | 10 | 0 | 0 | 4 | 11 | 96 | 0 | 21 | 0 | 0 | 0 | 0.5 |
|  | Olive oil | 4053 | **5** | 44 | 0 | 0 | 0 | 5 | 1 | 4 | 1 | 0 | 0 | 0 | 0 | 0 | 0 | 0 | 0 | 0 | 0 | 0 | 0 | 1 | 0 | 0 | 0 | 0 | 0 | 0 | 0 | 0 | 0 | 0 | 3 | 1.7 |
|  | Lentils, cooked | 16070 | **250** | 290 | 23 | 50 | 20 | 1 | 0 | 0 | 0 | 0 | 0 | 0 | 0 | 0 | 0 | 3 | 0 | 0 | 453 | 2 | 4 | 0 | 48 | 1 | 8 | 90 | 450 | 923 | 7 | 5 | 3 | 0 | 4 | 11.4 |
|  | Potatoes, cooked | 11367 | **45** | 39 | 1 | 9 | 1 | 0 | 0 | 0 | 0 | 0 | 0 | 0 | 0 | 0 | 0 | 1 | 0 | 0 | 4 | 0 | 3 | 0 | 4 | 0 | 0 | 9 | 18 | 148 | 0 | 2 | 0 | 0 | 1 | 1.5 |
|  | Onion, cooked | 11283 | **15** | 7 | 0 | 2 | 0 | 0 | 0 | 0 | 0 | 0 | 0 | 0 | 0 | 0 | 0 | 0 | 0 | 0 | 2 | 0 | 1 | 0 | 3 | 0 | 0 | 2 | 5 | 25 | 0 | 0 | 0 | 0 | 0 | 0.3 |
|  | Chard, cooked | 11148 | **15** | 3 | 0 | 1 | 0 | 0 | 0 | 0 | 0 | 0 | 0 | 0 | 46 | 0 | 0 | 0 | 0 | 0 | 1 | 0 | 3 | 0 | 9 | 0 | 0 | 13 | 5 | 82 | 0 | 27 | 0 | 0 | 49 | 0.1 |
|  | Vegetable oil | 4669 | **5** | 44 | 0 | 0 | 0 | 5 | 1 | 1 | 3 | 0 | 3 | 0 | 0 | 0 | 0 | 0 | 0 | 0 | 0 | 0 | 0 | 0 | 0 | 0 | 0 | 0 | 0 | 0 | 0 | 0 | 0 | 0 | 9 | 1.7 |
|  | Salt | 2047 | **0.25** | 0 | 0 | 0 | 0 | 0 | 0 | 0 | 0 | 0 | 0 | 0 | 0 | 0 | 0 | 0 | 0 | 0 | 0 | 0 | 0 | 0 | 0 | 0 | 0 | 0 | 0 | 0 | 0 | 97 | 0 |  | 0 | 0.0 |
|  | Kiwi fruit | 63126500 | **100** | 58 | 1 | 14 | 3 | 0 | 0 | 0 | 0 | 0 | 0 | 0 | 4 | 0 | 0 | 0 | 0 | 0 | 26 | 0 | 75 | 1 | 35 | 0 | 0 | 16 | 34 | 198 | 0 | 5 | 0 | 0 | 40 | 2.3 |
| **Afternoon tea** | Reduced fat milk | 1079 | **300** | 150 | 10 | 15 | 0 | 6 | 3 | 1 | 0 | 24 | 0 | 0 | 249 | 0 | 0 | 0 | 0 | 2 | 6 | 1 | 0 | 0 | 378 | 0 | 0 | 36 | 309 | 477 | 5 | 117 | 1 | 3 | 0 | 5.9 |
|  | Wholemeal bread | 18075 | **200** | 508 | 25 | 86 | 12 | 7 | 1 | 1 | 3 | 0 | 3 | 0 | 0 | 1 | 0 | 9 | 0 | 0 | 84 | 1 | 0 | 6 | 326 | 0 | 5 | 153 | 424 | 500 | 52 | 900 | 4 | 0 | 0 | 19.9 |
|  | Avocado | 9037 | **120** | 192 | 2 | 10 | 8 | 18 | 3 | 12 | 2 | 0 | 0 | 0 | 8 | 0 | 0 | 2 | 0 | 0 | 97 | 2 | 12 | 2 | 14 | 0 | 1 | 35 | 62 | 582 | 0 | 8 | 1 | 0 | 25 | 7.5 |
| **Dinner** | Broccoli, cooked | 11091 | **60** | 21 | 1 | 4 | 2 | 0 | 0 | 0 | 0 | 0 | 0 | 0 | 46 | 0 | 0 | 0 | 0 | 0 | 65 | 0 | 39 | 1 | 24 | 0 | 0 | 13 | 40 | 176 | 1 | 25 | 0 | 0 | 85 | 0.8 |
|  | Tomatoes | 11529 | **100** | 18 | 1 | 4 | 1 | 0 | 0 | 0 | 0 | 0 | 0 | 0 | 42 | 0 | 0 | 1 | 0 | 0 | 15 | 0 | 14 | 1 | 10 | 0 | 0 | 11 | 24 | 237 | 0 | 5 | 0 | 0 | 0 | 0.7 |
|  | Olive oil | 4053 | **5** | 44 | 0 | 0 | 0 | 5 | 1 | 4 | 1 | 0 | 0 | 0 | 0 | 0 | 0 | 0 | 0 | 0 | 0 | 0 | 0 | 1 | 0 | 0 | 0 | 0 | 0 | 0 | 0 | 0 | 0 | 0 | 3 | 1.7 |
|  | Salmon, cooked | 15212 | **100** | 153 | 25 | 0 | 0 | 5 | 1 | 2 | 1 | 55 | 0 | 1 | 42 | 0 | 0 | 10 | 1 | 5 | 5 | 1 | 0 | 0 | 8 | 0 | 0 | 32 | 313 | 439 | 38 | 90 | 0 | 13 | 1 | 6.0 |
|  | White rice, cooked | 169711 | **200** | 194 | 4 | 42 | 2 | 0 | 0 | 0 | 0 | 0 | 0 | 0 | 0 | 0 | 0 | 1 | 0 | 0 | 2 | 0 | 0 | 0 | 4 | 0 | 0 | 1 | 16 | 20 | 11 | 10 | 1 | 0 | 0 | 7.6 |
|  | Vegetable oil | 4669 | **5** | 44 | 0 | 0 | 0 | 5 | 1 | 1 | 3 | 0 | 3 | 0 | 0 | 0 | 0 | 0 | 0 | 0 | 0 | 0 | 0 | 0 | 0 | 0 | 0 | 0 | 0 | 0 | 0 | 0 | 0 | 0 | 9 | 1.7 |
|  | Salt | 2047 | **0.25** | 0 | 0 | 0 | 0 | 0 | 0 | 0 | 0 | 0 | 0 | 0 | 0 | 0 | 0 | 0 | 0 | 0 | 0 | 0 | 0 | 0 | 0 | 0 | 0 | 0 | 0 | 0 | 0 | 97 | 0 |  | 0 | 0.0 |
| **Total intake** | | | | **2553** | **123** | **315** | **70** | **97** | **18** | **39** | **31** | **103** | **21** | **6** | **943** | **2** | **2** | **30** | **3** | **8** | **841** | **11** | **277** | **23** | **1518** | **3** | **21** | **706** | **2503** | **5388** | **145** | **1541** | **16** | **20** | **263** | **100** |
| **Nutritional requirements ^c^** | | | | 2548 | 96 | 319 | 25 | 99 | 23 | 48 | 28 | 300 | 23 | 6 | 600 | 1 | 1 | 12 | 1 | 2 | 300 | 4 | 45 | 11 | 1300 | 1 | 8 | 240 | 1250 | 4500 | 40 | 1500 | 8 | 15 | 60 | - |
| **Percentage of adequacy (%)** | | | | 100 | 129 | 99 | 281 | 98 | 78 | 81 | 108 | 34 | 93 | 106 | 157 | 254 | 249 | 254 | 318 | 446 | 280 | 263 | 615 | 207 | 117 | 408 | 268 | 294 | 200 | 120 | 361 | 103 | 204 | 132 | 438 | - |
|  | | | | | | | | | | | | | | | | | | | | | | | | | | | | | | | | | | | | |
| **Components of the PHDI ^d^** | | | | **Nuts & peanuts** | | **Legumes** | | **Fruits** | | **Vegetables** | | **ReV ratio** | | **DGV ratio** | | **Whole cereals** | | **Tubers & potatoes** | | **Dairy products** | | **Eggs** | **Fish and seafood** | | **Chicken & substitutes** | | **Vegetable oils** | | **Red meats** | | **Animal fats** | | **Added sugars** | | **Total score**  **(points)** | |
| **Recommended percentages of total caloric intake ^d^**  **Optimal value (range)** | | | | 11.6  (0.0, 100) | | 11.3  (0.0, 100) | | 5.0  (0.0, 100) | | 3.1  (0.0, 100) | | 38.5  (0.0, 100) | | 29.5  (0.0, 100) | | 32.4  (0.0, 100) | | 1.6  (0.0, 3.1) | | 6.1  (0.0, 12.2) | | 0.8  (0.0, 1.5) | 1.6  (0.0, 5.7) | | 0.0  (0.0, 5.0) | | 16.5  (0.0, 30.7) | | 0.0  (0.0, 2.4) | | 0.0  (0.0, 1.4) | | 0.0  (0.0, 4.8) | | 150.0 | |
| **Actual percentages of total caloric intake** | | | | 14.3 | | 11.4 | | 5.8 | | 3.2 | | 37.5 | | 29.7 | | 24.1 | | 1.5 | | 11.8 | | 0.0 | 6.0 | | 0.0 | | 14.4 | | 0.0 | | 0.0 | | 0.0 | | - | |
| **PHDI score ^d^** | | | | 10.0 | | 10.0 | | 10.0 | | 10.0 | | 4.9 | | 5.0 | | 7.4 | | 9.5 | | 0.7 | | 0.0 | 0.0 | | 10.0 | | 8.8 | | 10.0 | | 10.0 | | 10.0 | | 116.3 | |
| **Components of the PHDI-C ^e^** | | | | **Nuts & peanuts** | | **Legumes** | | **Fruits** | | **Vegetables** | | **ReV ratio** | | **DGV ratio** | | **Cereals** | **WC ratio** | **Tubers & potatoes** | | **Dairy products** | | **Eggs and white meats** | | | | | **Vegetable oils** | **Palm oil** | **Red meats** | | **Animal fats** | | **Added sugars** | | **Total score**  **(points)** | |
| **Recommended percentages of total caloric intake ^e^**  **Optimal value (range)** | | | | 11.6  (0.0, 100) | | 11.3  (0.0, 100) | | 5.0  (0.0, 100) | | 3.1  (0.0, 100) | | 38.5  (0.0, 100) | | 29.5  (0.0, 100) | | 30.0 (0.0, 60.0) | 75.0 (0.0, 100) | 1.6  (0.0, 3.1) | | 12.2  (0.0, 24.4) | | 6.2  (0.0, 12.2) | | | | | 14.1  (0.0 28.3) | 0.0  (0.0, 2.4) | 0.0  (0.0, 2.4) | | 0.0  (0.0, 1.4) | | 0.0  (0.0, 4.8) | | 150.0 | |
| **Actual percentages of total caloric intake** | | | | 14.3 | | 11.4 | | 5.8 | | 3.2 | | 37.5 | | 29.7 | | 31.7 | 76.0 | 1.5 | | 11.8 | | 6.0 | | | | | 14.4 | 0.0 | 0.0 | | 0.0 | | 0.0 | | - | |
| **PHDI-C score ^e^** | | | | 10.0 | | 10.0 | | 10.0 | | 10.0 | | 4.9 | | 5.0 | | 9.4 | 9.6 | 9.5 | | 9.6 | | 9.7 | | | | | 9.9 | 10.0 | 10.0 | | 10.0 | | 10.0 | | 147.4 | |
| Abbreviations: USDA, United States Department of Agriculture; SAFA, saturated fatty acids; MUFAs, Monounsaturated fatty acids; PUFAs, Polyunsaturated fatty acids; PHDI, Planetary Health Diet Index; PHDI-C, Planetary Health Diet Index for children and adolescents; ReV ratio, red and orange vegetables ratio; DGV ratio, dark green vegetables ratio; WC ratio; whole cereals ratio.  ^a^ Each food item is color-coded with its corresponding index component.  ^b^ Food items' nutritional composition was obtained from the USDA National Nutrient Database (1).  ^c^ The caloric requirement of 2,548 kcal/day corresponds to the caloric requirement of a 42.3 kg boy aged 12 years whose level of physical activity is moderate to high (2). Macronutrient requirements were calculated based on acceptable macronutrient distribution ranges (3). Micronutrient requirements were defined based on Recommended Dietary Allowances or Average Intakes for children aged 9-13-year-old (3, 4).  ^d^ Components and scores correspond to the PHDI developed and validated by Cacau et al (5). Each component is associated to a recommended range of total caloric intake expressed as percentage of total calories, except for the ratio components which are expressed as percentage of total calories from vegetables. All components can score between 0 to 10 points, except for the ratio components which can score between 0 to 5 points, resulting in a total score of 150 points (5).  ^e^ Components and scores correspond to the PHDI-C proposed in this study. Each component is associated to a recommended range of total caloric intake expressed as percentage of total calories, except for the DGV ratio and ReV ratio components which are expressed as percentage of total calories from vegetables, and the WC ratio which is expressed as percentage of total calories from cereals. All components can score between 0 to 10 points, except for the DGV and ReV ratio components which can score between 0 to 5 points, resulting in a total score of 150 points. The formula to calculate the score for each component is provided in Table 2. | | | | | | | | | | | | | | | | | | | | | | | | | | | | | | | | | | | | |

# Supplemental table 3. Example of a sustainable healthy diet for a thirteen-year-old girl with a caloric requirement of 2,379 kcal/day.

| **Meal** | **Food item ^a^** | **USDA code ^b^** | **Consumed amount (g)** | **Energy (kcal)** | **Proteins (g)** | **Carbohydrates (g)** | **Fibre (g)** | **Lipids (g)** | **SAFAs (g)** | **MUFAs (g)** | **PUFAs (g)** | **Cholesterol (mg)** | **n-6 PUFAs (g)** | **n-3 PUFAs (g)** | **Vitamin A (mg)** | **Thiamine (mg)** | **Riboflavin (mg)** | **Niacin (mg)** | **Vitamin B6 (mg)** | **Vitamin B12 (mg)** | **Folate (mg)** | **Pantothenic acid (mg)** | **Vitamin C (mg)** | **Vitamin E (mg)** | **Calcium (mg)** | **Copper (mg)** | **Iron (mg)** | **Magnesium (mg)** | **Phosphorus (mg)** | **Potassium (mg)** | **Selenium (mg)** | **Sodium (mg)** | **Zinc (mg)** | **Vitamin D (mg)** | **Vitamin K (mg)** | **Percentage of total caloric intake (%)** |
| --- | --- | --- | --- | --- | --- | --- | --- | --- | --- | --- | --- | --- | --- | --- | --- | --- | --- | --- | --- | --- | --- | --- | --- | --- | --- | --- | --- | --- | --- | --- | --- | --- | --- | --- | --- | --- |
| **Breakfast** | Reduced fat milk | 1079 | **100** | 50 | 3 | 5 | 0 | 2 | 1 | 0 | 0 | 8 | 0 | 0 | 83 | 0 | 0 | 0 | 0 | 1 | 2 | 0 | 0 | 0 | 126 | 0 | 0 | 12 | 103 | 159 | 2 | 39 | 0 | 1 | 0 | 2.1 |
|  | Oats, cooked | 8121 | **150** | 107 | 4 | 18 | 3 | 2 | 0 | 1 | 1 | 0 | 1 | 0 | 0 | 0 | 0 | 0 | 0 | 0 | 9 | 0 | 0 | 0 | 14 | 0 | 1 | 41 | 116 | 105 | 8 | 6 | 2 | 0 | 0 | 4.5 |
|  | Strawberries | 9316 | **150** | 54 | 1 | 12 | 4 | 0 | 0 | 0 | 0 | 0 | 0 | 0 | 0 | 0 | 0 | 0 | 0 | 0 | 0 | 0 | 89 | 0 | 26 | 0 | 0 | 19 | 35 | 242 | 0 | 2 | 0 | 0 | 0 | 2.3 |
|  | Bananas | 9040 | **80** | 71 | 1 | 18 | 2 | 0 | 0 | 0 | 0 | 0 | 0 | 0 | 2 | 0 | 0 | 1 | 0 | 0 | 16 | 0 | 7 | 0 | 4 | 0 | 0 | 22 | 18 | 286 | 1 | 1 | 0 | 0 | 0 | 3.0 |
|  | Walnuts | 12154 | **30** | 186 | 7 | 3 | 2 | 18 | 1 | 5 | 11 | 0 | 10 | 1 | 1 | 0 | 0 | 0 | 0 | 0 | 9 | 0 | 1 | 1 | 18 | 0 | 1 | 60 | 154 | 157 | 5 | 1 | 1 | 0 | 1 | 7.8 |
|  | Chia seeds | 12006 | **20** | 97 | 3 | 8 | 7 | 6 | 1 | 0 | 5 | 0 | 1 | 4 | 0 | 0 | 0 | 2 |  | 0 | 10 |  | 0 | 0 | 126 | 0 | 2 | 67 | 81 | 81 | 11 | 3 | 1 |  |  | 4.1 |
| **Snack** | Almonds | 12061 | **20** | 116 | 4 | 4 | 3 | 10 | 1 | 6 | 2 | 0 | 2 | 0 | 0 | 0 | 0 | 1 | 0 | 0 | 9 | 0 | 0 | 5 | 54 | 0 | 1 | 54 | 96 | 147 | 1 | 0 | 1 | 0 | 0 | 4.9 |
| **Lunch** | Cabbage, raw | 11109 | **80** | 20 | 1 | 5 | 2 | 0 | 0 | 0 | 0 | 0 | 0 | 0 | 3 | 0 | 0 | 0 | 0 | 0 | 21 | 0 | 60 | 1 | 28 | 0 | 0 | 13 | 27 | 158 | 0 | 4 | 0 | 0 | 32 | 0.8 |
|  | Carrots, raw | 11124 | **35** | 14 | 0 | 3 | 1 | 0 | 0 | 0 | 0 | 0 | 0 | 0 | 292 | 0 | 0 | 0 | 0 | 0 | 7 | 0 | 2 | 0 | 12 | 0 | 0 | 4 | 12 | 112 | 0 | 24 | 0 | 0 | 0 | 0.6 |
|  | Olive oil | 4053 | **7.5** | 66 | 0 | 0 | 0 | 8 | 1 | 5 | 1 | 0 | 1 | 0 | 0 | 0 | 0 | 0 | 0 | 0 | 0 | 0 | 0 | 1 | 0 | 0 | 0 | 0 | 0 | 0 | 0 | 0 | 0 | 0 | 5 | 2.8 |
|  | Lentils, cooked | 16070 | **230** | 267 | 21 | 46 | 18 | 1 | 0 | 0 | 0 | 0 | 0 | 0 | 0 | 0 | 0 | 2 | 0 | 0 | 416 | 1 | 3 | 0 | 44 | 1 | 8 | 83 | 414 | 849 | 6 | 5 | 3 | 0 | 4 | 11.2 |
|  | Potatoes, cooked | 11367 | **45** | 39 | 1 | 9 | 1 | 0 | 0 | 0 | 0 | 0 | 0 | 0 | 0 | 0 | 0 | 1 | 0 | 0 | 4 | 0 | 3 | 0 | 4 | 0 | 0 | 9 | 18 | 148 | 0 | 2 | 0 | 0 | 1 | 1.6 |
|  | Onion, cooked | 11283 | **25** | 11 | 0 | 3 | 0 | 0 | 0 | 0 | 0 | 0 | 0 | 0 | 0 | 0 | 0 | 0 | 0 | 0 | 4 | 0 | 1 | 0 | 6 | 0 | 0 | 3 | 9 | 42 | 0 | 1 | 0 | 0 | 0 | 0.5 |
|  | Chard, cooked | 11148 | **35** | 7 | 1 | 1 | 1 | 0 | 0 | 0 | 0 | 0 | 0 | 0 | 107 | 0 | 0 | 0 | 0 | 0 | 3 | 0 | 6 | 1 | 20 | 0 | 1 | 30 | 12 | 192 | 0 | 63 | 0 | 0 | 114 | 0.3 |
|  | Vegetable oil | 4669 | **2.5** | 22 | 0 | 0 | 0 | 3 | 0 | 1 | 1 | 0 | 1 | 0 | 0 | 0 | 0 | 0 | 0 | 0 | 0 | 0 | 0 | 0 | 0 | 0 | 0 | 0 | 0 | 0 | 0 | 0 | 0 | 0 | 5 | 0.9 |
|  | Salt | 2047 | **0.5** | 0 | 0 | 0 | 0 | 0 | 0 | 0 | 0 | 0 | 0 | 0 | 0 | 0 | 0 | 0 | 0 | 0 | 0 | 0 | 0 | 0 | 0 | 0 | 0 | 0 | 0 | 0 | 0 | 194 | 0 |  | 0 | 0.0 |
|  | Kiwi fruit | 63126500 | **100** | 58 | 1 | 14 | 3 | 0 | 0 | 0 | 0 | 0 | 0 | 0 | 4 | 0 | 0 | 0 | 0 | 0 | 26 | 0 | 75 | 1 | 35 | 0 | 0 | 16 | 34 | 198 | 0 | 5 | 0 | 0 | 40 | 2.4 |
| **Afternoon tea** | Reduced fat milk | 1079 | **200** | 100 | 7 | 10 | 0 | 4 | 2 | 1 | 0 | 16 | 0 | 0 | 166 | 0 | 0 | 0 | 0 | 1 | 4 | 1 | 0 | 0 | 252 | 0 | 0 | 24 | 206 | 318 | 4 | 78 | 1 | 2 | 0 | 4.2 |
|  | Wholemeal bread | 18075 | **180** | 457 | 22 | 78 | 11 | 6 | 1 | 1 | 3 | 0 | 3 | 0 | 0 | 1 | 0 | 8 | 0 | 0 | 76 | 1 | 0 | 5 | 293 | 0 | 5 | 138 | 382 | 450 | 46 | 810 | 3 | 0 | 0 | 19.2 |
|  | Avocado | 9037 | **100** | 160 | 2 | 9 | 7 | 15 | 2 | 10 | 2 | 0 | 0 | 0 | 7 | 0 | 0 | 2 | 0 | 0 | 81 | 1 | 10 | 2 | 12 | 0 | 1 | 29 | 52 | 485 | 0 | 7 | 1 | 0 | 21 | 6.7 |
| **Dinner** | Broccoli, cooked | 11091 | **60** | 21 | 1 | 4 | 2 | 0 | 0 | 0 | 0 | 0 | 0 | 0 | 46 | 0 | 0 | 0 | 0 | 0 | 65 | 0 | 39 | 1 | 24 | 0 | 0 | 13 | 40 | 176 | 1 | 25 | 0 | 0 | 85 | 0.9 |
|  | Tomatoes | 11529 | **120** | 22 | 1 | 5 | 1 | 0 | 0 | 0 | 0 | 0 | 0 | 0 | 50 | 0 | 0 | 1 | 0 | 0 | 18 | 0 | 16 | 1 | 12 | 0 | 0 | 13 | 29 | 284 | 0 | 6 | 0 | 0 | 0 | 0.9 |
|  | Olive oil | 4053 | **7.5** | 66 | 0 | 0 | 0 | 8 | 1 | 5 | 1 | 0 | 1 | 0 | 0 | 0 | 0 | 0 | 0 | 0 | 0 | 0 | 0 | 1 | 0 | 0 | 0 | 0 | 0 | 0 | 0 | 0 | 0 | 0 | 5 | 2.8 |
|  | Salmon, cooked | 15212 | **100** | 153 | 25 | 0 | 0 | 5 | 1 | 2 | 1 | 55 | 0 | 1 | 42 | 0 | 0 | 10 | 1 | 5 | 5 | 1 | 0 | 0 | 8 | 0 | 0 | 32 | 313 | 439 | 38 | 90 | 0 | 13 | 1 | 6.4 |
|  | White rice, cooked | 169711 | **200** | 194 | 4 | 42 | 2 | 0 | 0 | 0 | 0 | 0 | 0 | 0 | 0 | 0 | 0 | 1 | 0 | 0 | 2 | 0 | 0 | 0 | 4 | 0 | 0 | 1 | 16 | 20 | 11 | 10 | 1 | 0 | 0 | 8.2 |
|  | Vegetable oil | 4669 | **2.5** | 22 | 0 | 0 | 0 | 3 | 0 | 1 | 1 | 0 | 1 | 0 | 0 | 0 | 0 | 0 | 0 | 0 | 0 | 0 | 0 | 0 | 0 | 0 | 0 | 0 | 0 | 0 | 0 | 0 | 0 | 0 | 5 | 0.9 |
|  | Salt | 2047 | **0.5** | 0 | 0 | 0 | 0 | 0 | 0 | 0 | 0 | 0 | 0 | 0 | 0 | 0 | 0 | 0 | 0 | 0 | 0 | 0 | 0 | 0 | 0 | 0 | 0 | 0 | 0 | 0 | 0 | 194 | 0 |  | 0 | 0.0 |
| **Total intake** | | | | **2380** | **111** | **297** | **69** | **91** | **14** | **38** | **30** | **79** | **21** | **6** | **804** | **2** | **2** | **29** | **3** | **6** | **786** | **9** | **313** | **21** | **1121** | **3** | **21** | **681** | **2165** | **5048** | **135** | **1568** | **15** | **16** | **318** | **100** |
| **Nutritional requirements ^c^** | | | | 2379 | 89 | 297 | 25 | 93 | 21 | 45 | 26 | 300 | 21 | 5 | 600 | 1 | 1 | 12 | 1 | 2 | 300 | 4 | 45 | 11 | 1300 | 1 | 8 | 240 | 1250 | 4500 | 40 | 1500 | 8 | 15 | 60 | - |
| **Percentage of adequacy (%)** | | | | 100 | 124 | 100 | 276 | 99 | 66 | 85 | 115 | 26 | 101 | 111 | 134 | 223 | 194 | 241 | 305 | 354 | 262 | 227 | 697 | 195 | 86 | 413 | 263 | 284 | 173 | 112 | 338 | 105 | 184 | 109 | 530 | - |
|  | | | | | | | | | | | | | | | | | | | | | | | | | | | | | | | | | | | | |
| **Components of the PHDI ^d^** | | | | **Nuts & peanuts** | | **Legumes** | | **Fruits** | | **Vegetables** | | **ReV ratio** | | **DGV ratio** | | **Whole cereals** | | **Tubers & potatoes** | | **Dairy products** | | **Eggs** | **Fish and seafood** | | **Chicken & substitutes** | | **Vegetable oils** | | **Red meats** | | **Animal fats** | | **Added sugars** | | **Total score**  **(points)** | |
| **Recommended percentages of total caloric intake ^d^**  **Optimal value (range)** | | | | 11.6  (0.0, 100) | | 11.3  (0.0, 100) | | 5.0  (0.0, 100) | | 3.1  (0.0, 100) | | 38.5  (0.0, 100) | | 29.5  (0.0, 100) | | 32.4  (0.0, 100) | | 1.6  (0.0, 3.1) | | 6.1  (0.0, 12.2) | | 0.8  (0.0, 1.5) | 1.6  (0.0, 5.7) | | 0.0  (0.0, 5.0) | | 16.5  (0.0, 30.7) | | 0.0  (0.0, 2.4) | | 0.0  (0.0, 1.4) | | 0.0  (0.0, 4.8) | | 150.0 | |
| **Actual percentages of total caloric intake** | | | | 16.8 | | 11.2 | | 7.7 | | 4.0 | | 37.9 | | 29.5 | | 23.7 | | 1.6 | | 6.3 | | 0.0 | 6.4 | | 0.0 | | 14.2 | | 0.0 | | 0.0 | | 0.0 | | - | |
| **PHDI score ^d^** | | | | 10.0 | | 9.9 | | 10.0 | | 10.0 | | 4.9 | | 5.0 | | 7.3 | | 10.0 | | 9.7 | | 0.0 | 0.0 | | 10.0 | | 8.6 | | 10.0 | | 10.0 | | 10.0 | | 125.4 | |
| **Components of the PHDI-C ^e^** | | | | **Nuts & peanuts** | | **Legumes** | | **Fruits** | | **Vegetables** | | **ReV ratio** | | **DGV ratio** | | **Cereals** | **WC ratio** | **Tubers & potatoes** | | **Dairy products** | | **Eggs and white meats** | | | | | **Vegetable oils** | **Palm oil** | **Red meats** | | **Animal fats** | | **Added sugars** | | **Total score**  **(points)** | |
| **Recommended percentages of total caloric intake ^e^**  **Optimal value (range)** | | | | 11.6  (0.0, 100) | | 11.3  (0.0, 100) | | 5.0  (0.0, 100) | | 3.1  (0.0, 100) | | 38.5  (0.0, 100) | | 29.5  (0.0, 100) | | 30.0 (0.0, 60.0) | 75.0 (0.0, 100) | 1.6  (0.0, 3.1) | | 6.1  (0.0, 12.2) | | 6.2  (0.0, 12.2) | | | | | 14.1  (0.0 28.3) | 0.0  (0.0, 2.4) | 0.0  (0.0, 2.4) | | 0.0  (0.0, 1.4) | | 0.0  (0.0, 4.8) | | 150.0 | |
| **Actual percentages of total caloric intake** | | | | 16.8 | | 11.2 | | 7.7 | | 4.0 | | 37.9 | | 29.5 | | 31.8 | 74.4 | 1.6 | | 6.3 | | 6.4 | | | | | 14.2 | 0.0 | 0.0 | | 0.0 | | 0.0 | | - | |
| **PHDI-C score ^e^** | | | | 10.0 | | 9.9 | | 10.0 | | 10.0 | | 4.9 | | 5.0 | | 9.4 | 9.9 | 10.0 | | 9.7 | | 9.6 | | | | | 10.0 | 10.0 | 10.0 | | 10.0 | | 10.0 | | 148.4 | |
| Abbreviations: USDA, United States Department of Agriculture; SAFA, saturated fatty acids; MUFAs, Monounsaturated fatty acids; PUFAs, Polyunsaturated fatty acids; PHDI, Planetary Health Diet Index; PHDI-C, Planetary Health Diet Index for children and adolescents; ReV ratio, red and orange vegetables ratio; DGV ratio, dark green vegetables ratio; WC ratio; whole cereals ratio.  ^a^ Each food item is color-coded with its corresponding index component.  ^b^ Food items' nutritional composition was obtained from the USDA National Nutrient Database (1).  ^c^ The caloric requirement of 2,379 kcal/day corresponds to the caloric requirement of a 48.3 kg girl aged 13 years whose level of physical activity is moderate to high (2). Macronutrient requirements were calculated based on acceptable macronutrient distribution ranges (3). Micronutrient requirements were defined based on Recommended Dietary Allowances or Average Intakes for children aged 9-13-year-old (3, 4).  ^d^ Components and scores correspond to the PHDI developed and validated by Cacau et al (5). Each component is associated to a recommended range of total caloric intake expressed as percentage of total calories, except for the ratio components which are expressed as percentage of total calories from vegetables. All components can score between 0 to 10 points, except for the ratio components which can score between 0 to 5 points, resulting in a total score of 150 points (5).  ^e^ Components and scores correspond to the PHDI-C proposed in this study. Each component is associated to a recommended range of total caloric intake expressed as percentage of total calories, except for the DGV ratio and ReV ratio components which are expressed as percentage of total calories from vegetables, and the WC ratio which is expressed as percentage of total calories from cereals. All components can score between 0 to 10 points, except for the DGV and ReV ratio components which can score between 0 to 5 points, resulting in a total score of 150 points. The formula to calculate the score for each component is provided in Table 2. | | | | | | | | | | | | | | | | | | | | | | | | | | | | | | | | | | | | |

# Supplemental table 4. Example of a sustainable healthy diet for an eighteen-year-old boy with a caloric requirement of 3,410 kcal/day.

| **Meal** | **Food item ^a^** | **USDA code ^b^** | **Consumed amount (g)** | **Energy (kcal)** | **Proteins (g)** | **Carbohydrates (g)** | **Fibre (g)** | **Lipids (g)** | **SAFAs (g)** | **MUFAs (g)** | **PUFAs (g)** | **Cholesterol (mg)** | **n-6 PUFAs (g)** | **n-3 PUFAs (g)** | **Vitamin A (mg)** | **Thiamine (mg)** | **Riboflavin (mg)** | **Niacin (mg)** | **Vitamin B6 (mg)** | **Vitamin B12 (mg)** | **Folate (mg)** | **Pantothenic acid (mg)** | **Vitamin C (mg)** | **Vitamin E (mg)** | **Calcium (mg)** | **Copper (mg)** | **Iron (mg)** | **Magnesium (mg)** | **Phosphorus (mg)** | **Potassium (mg)** | **Selenium (mg)** | **Sodium (mg)** | **Zinc (mg)** | **Vitamin D (mg)** | **Vitamin K (mg)** | **Percentage of total caloric intake (%)** |
| --- | --- | --- | --- | --- | --- | --- | --- | --- | --- | --- | --- | --- | --- | --- | --- | --- | --- | --- | --- | --- | --- | --- | --- | --- | --- | --- | --- | --- | --- | --- | --- | --- | --- | --- | --- | --- |
| **Breakfast** | Reduced fat milk | 1079 | **200** | 100 | 7 | 10 | 0 | 4 | 2 | 1 | 0 | 16 | 0 | 0 | 166 | 0 | 0 | 0 | 0 | 1 | 4 | 1 | 0 | 0 | 252 | 0 | 0 | 24 | 206 | 318 | 4 | 78 | 1 | 2 | 0 | 2.9 |
|  | Oats, cooked | 8121 | **250** | 178 | 6 | 30 | 4 | 4 | 1 | 1 | 1 | 0 | 1 | 0 | 0 | 0 | 0 | 1 | 0 | 0 | 15 | 1 | 0 | 0 | 23 | 0 | 2 | 68 | 193 | 175 | 14 | 10 | 3 | 0 | 1 | 5.2 |
|  | Strawberries | 9316 | **200** | 72 | 1 | 16 | 5 | 0 | 0 | 0 | 0 | 0 | 0 | 0 | 0 | 0 | 0 | 0 | 0 | 0 | 0 | 0 | 119 | 0 | 34 | 0 | 1 | 25 | 46 | 322 | 0 | 2 | 0 | 0 | 0 | 2.1 |
|  | Bananas | 9040 | **150** | 134 | 2 | 34 | 4 | 0 | 0 | 0 | 0 | 0 | 0 | 0 | 5 | 0 | 0 | 1 | 1 | 0 | 30 | 1 | 13 | 0 | 8 | 0 | 0 | 41 | 33 | 537 | 2 | 2 | 0 | 0 | 1 | 3.9 |
|  | Walnuts | 12154 | **50** | 310 | 12 | 5 | 3 | 30 | 2 | 8 | 18 | 0 | 17 | 1 | 1 | 0 | 0 | 0 | 0 | 0 | 16 | 1 | 1 | 1 | 31 | 1 | 2 | 101 | 257 | 262 | 9 | 1 | 2 | 0 | 1 | 9.1 |
|  | Chia seeds | 12006 | **30** | 146 | 5 | 13 | 10 | 9 | 1 | 1 | 7 | 0 | 2 | 5 | 0 | 0 | 0 | 3 |  | 0 | 15 |  | 0 | 0 | 189 | 0 | 2 | 101 | 122 | 122 | 17 | 5 | 1 |  |  | 4.3 |
| **Snack** | Almonds | 12061 | **30** | 174 | 6 | 6 | 4 | 15 | 1 | 9 | 4 | 0 | 4 | 0 | 0 | 0 | 0 | 1 | 0 | 0 | 13 | 0 | 0 | 8 | 81 | 0 | 1 | 81 | 144 | 220 | 1 | 0 | 1 | 0 | 0 | 5.1 |
| **Lunch** | Cabbage, raw | 11109 | **100** | 25 | 1 | 6 | 3 | 0 | 0 | 0 | 0 | 0 | 0 | 0 | 4 | 0 | 0 | 0 | 0 | 0 | 26 | 0 | 75 | 1 | 35 | 0 | 0 | 16 | 34 | 198 | 0 | 5 | 0 | 0 | 40 | 0.7 |
|  | Carrots, raw | 11124 | **50** | 21 | 0 | 5 | 1 | 0 | 0 | 0 | 0 | 0 | 0 | 0 | 418 | 0 | 0 | 0 | 0 | 0 | 10 | 0 | 3 | 0 | 17 | 0 | 0 | 6 | 18 | 160 | 0 | 35 | 0 | 0 | 0 | 0.6 |
|  | Olive oil | 4053 | **7.5** | 66 | 0 | 0 | 0 | 8 | 1 | 5 | 1 | 0 | 1 | 0 | 0 | 0 | 0 | 0 | 0 | 0 | 0 | 0 | 0 | 1 | 0 | 0 | 0 | 0 | 0 | 0 | 0 | 0 | 0 | 0 | 5 | 1.9 |
|  | Lentils, cooked | 16070 | **350** | 406 | 32 | 70 | 28 | 1 | 0 | 0 | 1 | 0 | 0 | 0 | 0 | 1 | 0 | 4 | 1 | 0 | 634 | 2 | 5 | 0 | 67 | 1 | 12 | 126 | 630 | 1292 | 10 | 7 | 4 | 0 | 6 | 11.9 |
|  | Potatoes, cooked | 11367 | **60** | 52 | 1 | 12 | 1 | 0 | 0 | 0 | 0 | 0 | 0 | 0 | 0 | 0 | 0 | 1 | 0 | 0 | 5 | 0 | 4 | 0 | 5 | 0 | 0 | 12 | 24 | 197 | 0 | 3 | 0 | 0 | 1 | 1.5 |
|  | Onion, cooked | 11283 | **30** | 13 | 0 | 3 | 0 | 0 | 0 | 0 | 0 | 0 | 0 | 0 | 0 | 0 | 0 | 0 | 0 | 0 | 5 | 0 | 2 | 0 | 7 | 0 | 0 | 3 | 11 | 50 | 0 | 1 | 0 | 0 | 0 | 0.4 |
|  | Chard, cooked | 11148 | **50** | 10 | 1 | 2 | 1 | 0 | 0 | 0 | 0 | 0 | 0 | 0 | 153 | 0 | 0 | 0 | 0 | 0 | 5 | 0 | 9 | 1 | 29 | 0 | 1 | 43 | 17 | 275 | 0 | 90 | 0 | 0 | 164 | 0.3 |
|  | Vegetable oil | 4669 | **5** | 44 | 0 | 0 | 0 | 5 | 1 | 1 | 3 | 0 | 3 | 0 | 0 | 0 | 0 | 0 | 0 | 0 | 0 | 0 | 0 | 0 | 0 | 0 | 0 | 0 | 0 | 0 | 0 | 0 | 0 | 0 | 9 | 1.3 |
|  | Salt | 2047 | **0** | 0 | 0 | 0 | 0 | 0 | 0 | 0 | 0 | 0 | 0 | 0 | 0 | 0 | 0 | 0 | 0 | 0 | 0 | 0 | 0 | 0 | 0 | 0 | 0 | 0 | 0 | 0 | 0 | 0 | 0 |  | 0 | 0.0 |
|  | Kiwi fruit | 63126500 | **120** | 70 | 1 | 17 | 4 | 1 | 0 | 0 | 0 | 0 | 0 | 0 | 5 | 0 | 0 | 0 | 0 | 0 | 31 | 0 | 90 | 2 | 42 | 0 | 0 | 19 | 41 | 238 | 0 | 6 | 0 | 0 | 48 | 2.0 |
| **Afternoon tea** | Reduced fat milk | 1079 | **200** | 100 | 7 | 10 | 0 | 4 | 2 | 1 | 0 | 16 | 0 | 0 | 166 | 0 | 0 | 0 | 0 | 1 | 4 | 1 | 0 | 0 | 252 | 0 | 0 | 24 | 206 | 318 | 4 | 78 | 1 | 2 | 0 | 2.9 |
|  | Wholemeal bread | 18075 | **240** | 610 | 30 | 103 | 14 | 9 | 2 | 1 | 4 | 0 | 4 | 0 | 0 | 1 | 0 | 11 | 1 | 0 | 101 | 2 | 0 | 7 | 391 | 1 | 6 | 184 | 509 | 600 | 62 | 1080 | 4 | 0 | 0 | 17.9 |
|  | Avocado | 9037 | **150** | 240 | 3 | 13 | 10 | 22 | 3 | 15 | 3 | 0 | 0 | 0 | 11 | 0 | 0 | 3 | 0 | 0 | 122 | 2 | 15 | 3 | 18 | 0 | 1 | 44 | 78 | 728 | 1 | 11 | 1 | 0 | 32 | 7.0 |
| **Dinner** | Broccoli, cooked | 11091 | **75** | 26 | 2 | 5 | 2 | 0 | 0 | 0 | 0 | 0 | 0 | 0 | 58 | 0 | 0 | 0 | 0 | 0 | 81 | 0 | 49 | 1 | 30 | 0 | 1 | 16 | 50 | 220 | 1 | 31 | 0 | 0 | 106 | 0.8 |
|  | Tomatoes | 11529 | **150** | 27 | 1 | 6 | 2 | 0 | 0 | 0 | 0 | 0 | 0 | 0 | 63 | 0 | 0 | 1 | 0 | 0 | 23 | 0 | 21 | 1 | 15 | 0 | 0 | 17 | 36 | 356 | 0 | 8 | 0 | 0 | 0 | 0.8 |
|  | Olive oil | 4053 | **7.5** | 66 | 0 | 0 | 0 | 8 | 1 | 5 | 1 | 0 | 1 | 0 | 0 | 0 | 0 | 0 | 0 | 0 | 0 | 0 | 0 | 1 | 0 | 0 | 0 | 0 | 0 | 0 | 0 | 0 | 0 | 0 | 5 | 1.9 |
|  | Salmon, cooked | 15212 | **140** | 214 | 34 | 0 | 0 | 7 | 1 | 2 | 1 | 77 | 0 | 1 | 59 | 0 | 0 | 13 | 1 | 7 | 7 | 2 | 0 | 1 | 11 | 0 | 1 | 45 | 438 | 615 | 53 | 126 | 1 | 18 | 1 | 6.3 |
|  | White rice, cooked | 169711 | **250** | 243 | 5 | 53 | 3 | 0 | 0 | 0 | 0 | 0 | 0 | 0 | 0 | 0 | 0 | 1 | 0 | 0 | 3 | 1 | 0 | 0 | 5 | 0 | 0 | 1 | 20 | 25 | 14 | 13 | 1 | 0 | 0 | 7.1 |
|  | Vegetable oil | 4669 | **8** | 71 | 0 | 0 | 0 | 8 | 1 | 2 | 5 | 0 | 4 | 1 | 0 | 0 | 0 | 0 | 0 | 0 | 0 | 0 | 0 | 1 | 0 | 0 | 0 | 0 | 0 | 0 | 0 | 0 | 0 | 0 | 15 | 2.1 |
|  | Salt | 2047 | **0** | 0 | 0 | 0 | 0 | 0 | 0 | 0 | 0 | 0 | 0 | 0 | 0 | 0 | 0 | 0 | 0 | 0 | 0 | 0 | 0 | 0 | 0 | 0 | 0 | 0 | 0 | 0 | 0 | 0 | 0 |  | 0 | 0.0 |
| **Total intake** | | | | **3415** | **158** | **419** | **100** | **135** | **20** | **54** | **49** | **109** | **35** | **9** | **1107** | **3** | **2** | **41** | **4** | **9** | **1146** | **13** | **405** | **30** | **1539** | **4** | **31** | **994** | **3111** | **7224** | **190** | **1589** | **21** | **23** | **433** | **100** |
| **Nutritional requirements ^c^** | | | | 3410 | 128 | 426 | 25 | 133 | 30 | 64 | 38 | 300 | 30 | 8 | 900 | 1 | 1 | 16 | 1 | 2 | 400 | 5 | 75 | 15 | 1300 | 1 | 11 | 410 | 1250 | 4700 | 55 | 1500 | 11 | 15 | 75 | - |
| **Percentage of adequacy (%)** | | | | 100 | 124 | 98 | 399 | 102 | 66 | 83 | 130 | 36 | 117 | 121 | 123 | 237 | 191 | 254 | 340 | 368 | 287 | 262 | 540 | 198 | 118 | 475 | 280 | 242 | 249 | 154 | 345 | 106 | 194 | 151 | 578 | - |
|  | | | | | | | | | | | | | | | | | | | | | | | | | | | | | | | | | | | | |
| **Components of the PHDI ^d^** | | | | **Nuts & peanuts** | | **Legumes** | | **Fruits** | | **Vegetables** | | **ReV ratio** | | **DGV ratio** | | **Whole cereals** | | **Tubers & potatoes** | | **Dairy products** | | **Eggs** | **Fish and seafood** | | **Chicken & substitutes** | | **Vegetable oils** | | **Red meats** | | **Animal fats** | | **Added sugars** | | **Total score**  **(points)** | |
| **Recommended percentages of total caloric intake ^d^**  **Optimal value (range)** | | | | 11.6  (0.0, 100) | | 11.3  (0.0, 100) | | 5.0  (0.0, 100) | | 3.1  (0.0, 100) | | 38.5  (0.0, 100) | | 29.5  (0.0, 100) | | 32.4  (0.0, 100) | | 1.6  (0.0, 3.1) | | 6.1  (0.0, 12.2) | | 0.8  (0.0, 1.5) | 1.6  (0.0, 5.7) | | 0.0  (0.0, 5.0) | | 16.5  (0.0, 30.7) | | 0.0  (0.0, 2.4) | | 0.0  (0.0, 1.4) | | 0.0  (0.0, 4.8) | | 150.0 | |
| **Actual percentages of total caloric intake** | | | | 18.4 | | 11.9 | | 8.1 | | 3.6 | | 39.0 | | 29.7 | | 23.0 | | 1.5 | | 5.9 | | 0.0 | 6.3 | | 0.0 | | 14.3 | | 0.0 | | 0.0 | | 0.0 | | - | |
| **PHDI score ^d^** | | | | 10.0 | | 10.0 | | 10.0 | | 10.0 | | 5.0 | | 5.0 | | 7.1 | | 9.4 | | 9.6 | | 0.0 | 0.0 | | 10.0 | | 8.7 | | 10.0 | | 10.0 | | 10.0 | | 124.8 | |
| **Components of the PHDI-C ^e^** | | | | **Nuts & peanuts** | | **Legumes** | | **Fruits** | | **Vegetables** | | **ReV ratio** | | **DGV ratio** | | **Cereals** | **WC ratio** | **Tubers & potatoes** | | **Dairy products** | | **Eggs and white meats** | | | | | **Vegetable oils** | **Palm oil** | **Red meats** | | **Animal fats** | | **Added sugars** | | **Total score**  **(points)** | |
| **Recommended percentages of total caloric intake ^e^**  **Optimal value (range)** | | | | 11.6  (0.0, 100) | | 11.3  (0.0, 100) | | 5.0  (0.0, 100) | | 3.1  (0.0, 100) | | 38.5  (0.0, 100) | | 29.5  (0.0, 100) | | 30.0 (0.0, 60.0) | 75.0 (0.0, 100) | 1.6  (0.0, 3.1) | | 6.1  (0.0, 12.2) | | 6.2  (0.0, 12.2) | | | | | 14.1  (0.0 28.3) | 0.0  (0.0, 2.4) | 0.0  (0.0, 2.4) | | 0.0  (0.0, 1.4) | | 0.0  (0.0, 4.8) | | 150.0 | |
| **Actual percentages of total caloric intake** | | | | 18.4 | | 11.9 | | 8.1 | | 3.6 | | 39.0 | | 29.7 | | 30.1 | 76.4 | 1.5 | | 5.9 | | 6.3 | | | | | 14.3 | 0.0 | 0.0 | | 0.0 | | 0.0 | | - | |
| **PHDI-C score ^e^** | | | | 10.0 | | 10.0 | | 10.0 | | 10.0 | | 5.0 | | 5.0 | | 10.0 | 9.4 | 9.4 | | 9.6 | | 9.9 | | | | | 9.9 | 10.0 | 10.0 | | 10.0 | | 10.0 | | 148.2 | |
| Abbreviations: USDA, United States Department of Agriculture; SAFA, saturated fatty acids; MUFAs, Monounsaturated fatty acids; PUFAs, Polyunsaturated fatty acids; PHDI, Planetary Health Diet Index; PHDI-C, Planetary Health Diet Index for children and adolescents; ReV ratio, red and orange vegetables ratio; DGV ratio, dark green vegetables ratio; WC ratio; whole cereals ratio.  ^a^ Each food item is color-coded with its corresponding index component.  ^b^ Food items' nutritional composition was obtained from the USDA National Nutrient Database (1).  ^c^ The caloric requirement of 3,410 kcal/day corresponds to the caloric requirement of a 67.8 kg boy aged 18 years whose level of physical activity is moderate to high (2). Macronutrient requirements were calculated based on acceptable macronutrient distribution ranges (3). Micronutrient requirements were defined based on Recommended Dietary Allowances or Average Intakes for children aged 14-18-year-old (3, 4).  ^d^ Components and scores correspond to the PHDI developed and validated by Cacau et al (5). Each component is associated to a recommended range of total caloric intake expressed as percentage of total calories, except for the ratio components which are expressed as percentage of total calories from vegetables. All components can score between 0 to 10 points, except for the ratio components which can score between 0 to 5 points, resulting in a total score of 150 points (5).  ^e^ Components and scores correspond to the PHDI-C proposed in this study. Each component is associated to a recommended range of total caloric intake expressed as percentage of total calories, except for the DGV ratio and ReV ratio components which are expressed as percentage of total calories from vegetables, and the WC ratio which is expressed as percentage of total calories from cereals. All components can score between 0 to 10 points, except for the DGV and ReV ratio components which can score between 0 to 5 points, resulting in a total score of 150 points. The formula to calculate the score for each component is provided in Table 2. | | | | | | | | | | | | | | | | | | | | | | | | | | | | | | | | | | | | |

# Supplemental table 5. Example of a sustainable healthy diet for an eighteen-year-old girl of reproductive age with a caloric requirement of 2,503 kcal/day.

| **Meal** | **Food item ^a^** | **USDA code ^b^** | **Consumed amount (g)** | **Energy (kcal)** | **Proteins (g)** | **Carbohydrates (g)** | **Fibre (g)** | **Lipids (g)** | **SAFAs (g)** | **MUFAs (g)** | **PUFAs (g)** | **Cholesterol (mg)** | **n-6 PUFAs (g)** | **n-3 PUFAs (g)** | **Vitamin A (mg)** | **Thiamine (mg)** | **Riboflavin (mg)** | **Niacin (mg)** | **Vitamin B6 (mg)** | **Vitamin B12 (mg)** | **Folate (mg)** | **Pantothenic acid (mg)** | **Vitamin C (mg)** | **Vitamin E (mg)** | **Calcium (mg)** | **Copper (mg)** | **Iron (mg)** | **Magnesium (mg)** | **Phosphorus (mg)** | **Potassium (mg)** | **Selenium (mg)** | **Sodium (mg)** | **Zinc (mg)** | **Vitamin D (mg)** | **Vitamin K (mg)** | **Percentage of total caloric intake (%)** |
| --- | --- | --- | --- | --- | --- | --- | --- | --- | --- | --- | --- | --- | --- | --- | --- | --- | --- | --- | --- | --- | --- | --- | --- | --- | --- | --- | --- | --- | --- | --- | --- | --- | --- | --- | --- | --- |
| **Breakfast** | Reduced fat milk | 1079 | **200** | 100 | 7 | 10 | 0 | 4 | 2 | 1 | 0 | 16 | 0 | 0 | 166 | 0 | 0 | 0 | 0 | 1 | 4 | 1 | 0 | 0 | 252 | 0 | 0 | 24 | 206 | 318 | 4 | 78 | 1 | 2 | 0 | 4.0 |
|  | Oats, cooked | 8121 | **150** | 107 | 4 | 18 | 3 | 2 | 0 | 1 | 1 | 0 | 1 | 0 | 0 | 0 | 0 | 0 | 0 | 0 | 9 | 0 | 0 | 0 | 14 | 0 | 1 | 41 | 116 | 105 | 8 | 6 | 2 | 0 | 0 | 4.3 |
|  | Strawberries | 9316 | **120** | 43 | 1 | 10 | 3 | 0 | 0 | 0 | 0 | 0 | 0 | 0 | 0 | 0 | 0 | 0 | 0 | 0 | 0 | 0 | 72 | 0 | 20 | 0 | 0 | 15 | 28 | 193 | 0 | 1 | 0 | 0 | 0 | 1.7 |
|  | Bananas | 9040 | **80** | 71 | 1 | 18 | 2 | 0 | 0 | 0 | 0 | 0 | 0 | 0 | 2 | 0 | 0 | 1 | 0 | 0 | 16 | 0 | 7 | 0 | 4 | 0 | 0 | 22 | 18 | 286 | 1 | 1 | 0 | 0 | 0 | 2.9 |
|  | Walnuts | 12154 | **30** | 186 | 7 | 3 | 2 | 18 | 1 | 5 | 11 | 0 | 10 | 1 | 1 | 0 | 0 | 0 | 0 | 0 | 9 | 0 | 1 | 1 | 18 | 0 | 1 | 60 | 154 | 157 | 5 | 1 | 1 | 0 | 1 | 7.4 |
|  | Chia seeds | 12006 | **20** | 97 | 3 | 8 | 7 | 6 | 1 | 0 | 5 | 0 | 1 | 4 | 0 | 0 | 0 | 2 |  | 0 | 10 |  | 0 | 0 | 126 | 0 | 2 | 67 | 81 | 81 | 11 | 3 | 1 |  |  | 3.9 |
| **Snack** | Almonds | 12061 | **30** | 174 | 6 | 6 | 4 | 15 | 1 | 9 | 4 | 0 | 4 | 0 | 0 | 0 | 0 | 1 | 0 | 0 | 13 | 0 | 0 | 8 | 81 | 0 | 1 | 81 | 144 | 220 | 1 | 0 | 1 | 0 | 0 | 7.0 |
| **Lunch** | Cabbage, raw | 11109 | **80** | 20 | 1 | 5 | 2 | 0 | 0 | 0 | 0 | 0 | 0 | 0 | 3 | 0 | 0 | 0 | 0 | 0 | 21 | 0 | 60 | 1 | 28 | 0 | 0 | 13 | 27 | 158 | 0 | 4 | 0 | 0 | 32 | 0.8 |
|  | Carrots, raw | 11124 | **30** | 12 | 0 | 3 | 1 | 0 | 0 | 0 | 0 | 0 | 0 | 0 | 251 | 0 | 0 | 0 | 0 | 0 | 6 | 0 | 2 | 0 | 10 | 0 | 0 | 4 | 11 | 96 | 0 | 21 | 0 | 0 | 0 | 0.5 |
|  | Olive oil | 4053 | **5** | 44 | 0 | 0 | 0 | 5 | 1 | 4 | 1 | 0 | 0 | 0 | 0 | 0 | 0 | 0 | 0 | 0 | 0 | 0 | 0 | 1 | 0 | 0 | 0 | 0 | 0 | 0 | 0 | 0 | 0 | 0 | 3 | 1.8 |
|  | Lentils, cooked | 16070 | **250** | 290 | 23 | 50 | 20 | 1 | 0 | 0 | 0 | 0 | 0 | 0 | 0 | 0 | 0 | 3 | 0 | 0 | 453 | 2 | 4 | 0 | 48 | 1 | 8 | 90 | 450 | 923 | 7 | 5 | 3 | 0 | 4 | 11.6 |
|  | Potatoes, cooked | 11367 | **45** | 39 | 1 | 9 | 1 | 0 | 0 | 0 | 0 | 0 | 0 | 0 | 0 | 0 | 0 | 1 | 0 | 0 | 4 | 0 | 3 | 0 | 4 | 0 | 0 | 9 | 18 | 148 | 0 | 2 | 0 | 0 | 1 | 1.5 |
|  | Onion, cooked | 11283 | **15** | 7 | 0 | 2 | 0 | 0 | 0 | 0 | 0 | 0 | 0 | 0 | 0 | 0 | 0 | 0 | 0 | 0 | 2 | 0 | 1 | 0 | 3 | 0 | 0 | 2 | 5 | 25 | 0 | 0 | 0 | 0 | 0 | 0.3 |
|  | Chard, cooked | 11148 | **15** | 3 | 0 | 1 | 0 | 0 | 0 | 0 | 0 | 0 | 0 | 0 | 46 | 0 | 0 | 0 | 0 | 0 | 1 | 0 | 3 | 0 | 9 | 0 | 0 | 13 | 5 | 82 | 0 | 27 | 0 | 0 | 49 | 0.1 |
|  | Vegetable oil | 4669 | **5** | 44 | 0 | 0 | 0 | 5 | 1 | 1 | 3 | 0 | 3 | 0 | 0 | 0 | 0 | 0 | 0 | 0 | 0 | 0 | 0 | 0 | 0 | 0 | 0 | 0 | 0 | 0 | 0 | 0 | 0 | 0 | 9 | 1.8 |
|  | Salt | 2047 | **0.25** | 0 | 0 | 0 | 0 | 0 | 0 | 0 | 0 | 0 | 0 | 0 | 0 | 0 | 0 | 0 | 0 | 0 | 0 | 0 | 0 | 0 | 0 | 0 | 0 | 0 | 0 | 0 | 0 | 97 | 0 |  | 0 | 0.0 |
|  | Kiwi fruit | 63126500 | **100** | 58 | 1 | 14 | 3 | 0 | 0 | 0 | 0 | 0 | 0 | 0 | 4 | 0 | 0 | 0 | 0 | 0 | 26 | 0 | 75 | 1 | 35 | 0 | 0 | 16 | 34 | 198 | 0 | 5 | 0 | 0 | 40 | 2.3 |
| **Afternoon tea** | Reduced fat milk | 1079 | **100** | 50 | 3 | 5 | 0 | 2 | 1 | 0 | 0 | 8 | 0 | 0 | 83 | 0 | 0 | 0 | 0 | 1 | 2 | 0 | 0 | 0 | 126 | 0 | 0 | 12 | 103 | 159 | 2 | 39 | 0 | 1 | 0 | 2.0 |
|  | Wholemeal bread | 18075 | **200** | 508 | 25 | 86 | 12 | 7 | 1 | 1 | 3 | 0 | 3 | 0 | 0 | 1 | 0 | 9 | 0 | 0 | 84 | 1 | 0 | 6 | 326 | 0 | 5 | 153 | 424 | 500 | 52 | 900 | 4 | 0 | 0 | 20.3 |
|  | Avocado | 9037 | **120** | 192 | 2 | 10 | 8 | 18 | 3 | 12 | 2 | 0 | 0 | 0 | 8 | 0 | 0 | 2 | 0 | 0 | 97 | 2 | 12 | 2 | 14 | 0 | 1 | 35 | 62 | 582 | 0 | 8 | 1 | 0 | 25 | 7.7 |
| **Dinner** | Broccoli, cooked | 11091 | **60** | 21 | 1 | 4 | 2 | 0 | 0 | 0 | 0 | 0 | 0 | 0 | 46 | 0 | 0 | 0 | 0 | 0 | 65 | 0 | 39 | 1 | 24 | 0 | 0 | 13 | 40 | 176 | 1 | 25 | 0 | 0 | 85 | 0.8 |
|  | Tomatoes | 11529 | **100** | 18 | 1 | 4 | 1 | 0 | 0 | 0 | 0 | 0 | 0 | 0 | 42 | 0 | 0 | 1 | 0 | 0 | 15 | 0 | 14 | 1 | 10 | 0 | 0 | 11 | 24 | 237 | 0 | 5 | 0 | 0 | 0 | 0.7 |
|  | Olive oil | 4053 | **5** | 44 | 0 | 0 | 0 | 5 | 1 | 4 | 1 | 0 | 0 | 0 | 0 | 0 | 0 | 0 | 0 | 0 | 0 | 0 | 0 | 1 | 0 | 0 | 0 | 0 | 0 | 0 | 0 | 0 | 0 | 0 | 3 | 1.8 |
|  | Salmon, cooked | 15212 | **100** | 153 | 25 | 0 | 0 | 5 | 1 | 2 | 1 | 55 | 0 | 1 | 42 | 0 | 0 | 10 | 1 | 5 | 5 | 1 | 0 | 0 | 8 | 0 | 0 | 32 | 313 | 439 | 38 | 90 | 0 | 13 | 1 | 6.1 |
|  | White rice, cooked | 169711 | **200** | 194 | 4 | 42 | 2 | 0 | 0 | 0 | 0 | 0 | 0 | 0 | 0 | 0 | 0 | 1 | 0 | 0 | 2 | 0 | 0 | 0 | 4 | 0 | 0 | 1 | 16 | 20 | 11 | 10 | 1 | 0 | 0 | 7.8 |
|  | Vegetable oil | 4669 | **2.5** | 22 | 0 | 0 | 0 | 3 | 0 | 1 | 1 | 0 | 1 | 0 | 0 | 0 | 0 | 0 | 0 | 0 | 0 | 0 | 0 | 0 | 0 | 0 | 0 | 0 | 0 | 0 | 0 | 0 | 0 | 0 | 5 | 0.9 |
|  | Salt | 2047 | **0.25** | 0 | 0 | 0 | 0 | 0 | 0 | 0 | 0 | 0 | 0 | 0 | 0 | 0 | 0 | 0 | 0 | 0 | 0 | 0 | 0 | 0 | 0 | 0 | 0 | 0 | 0 | 0 | 0 | 97 | 0 |  | 0 | 0.0 |
| **Total intake** | | | | **2497** | **117** | **308** | **73** | **97** | **15** | **40** | **33** | **79** | **24** | **6** | **694** | **2** | **2** | **31** | **3** | **6** | **844** | **10** | **291** | **24** | **1164** | **3** | **22** | **711** | **2279** | **5103** | **141** | **1425** | **16** | **16** | **259** | **100** |
| **Nutritional requirements ^c^** | | | | 2503 | 94 | 313 | 25 | 97 | 22 | 47 | 28 | 300 | 22 | 6 | 700 | 1 | 1 | 14 | 1 | 2 | 300 | 5 | 65 | 15 | 1300 | 1 | 15 | 360 | 1250 | 4700 | 55 | 1500 | 9 | 15 | 75 | - |
| **Percentage of adequacy (%)** | | | | 100 | 124 | 98 | 290 | 100 | 66 | 86 | 120 | 26 | 106 | 109 | 99 | 213 | 191 | 218 | 261 | 266 | 281 | 192 | 447 | 160 | 90 | 344 | 147 | 198 | 182 | 109 | 257 | 95 | 173 | 109 | 345 | - |
|  | | | | | | | | | | | | | | | | | | | | | | | | | | | | | | | | | | | | |
| **Components of the PHDI ^d^** | | | | **Nuts & peanuts** | | **Legumes** | | **Fruits** | | **Vegetables** | | **ReV ratio** | | **DGV ratio** | | **Whole cereals** | | **Tubers & potatoes** | | **Dairy products** | | **Eggs** | **Fish and seafood** | | **Chicken & substitutes** | | **Vegetable oils** | | **Red meats** | | **Animal fats** | | **Added sugars** | | **Total score**  **(points)** | |
| **Recommended percentages of total caloric intake ^d^**  **Optimal value (range)** | | | | 11.6  (0.0, 100) | | 11.3  (0.0, 100) | | 5.0  (0.0, 100) | | 3.1  (0.0, 100) | | 38.5  (0.0, 100) | | 29.5  (0.0, 100) | | 32.4  (0.0, 100) | | 1.6  (0.0, 3.1) | | 6.1  (0.0, 12.2) | | 0.8  (0.0, 1.5) | 1.6  (0.0, 5.7) | | 0.0  (0.0, 5.0) | | 16.5  (0.0, 30.7) | | 0.0  (0.0, 2.4) | | 0.0  (0.0, 1.4) | | 0.0  (0.0, 4.8) | | 150.0 | |
| **Actual percentages of total caloric intake** | | | | 18.3 | | 11.6 | | 6.9 | | 3.2 | | 37.5 | | 29.7 | | 24.6 | | 1.5 | | 6.0 | | 0.0 | 6.1 | | 0.0 | | 13.9 | | 0.0 | | 0.0 | | 0.0 | | - | |
| **PHDI score ^d^** | | | | 10.0 | | 10.0 | | 10.0 | | 10.0 | | 4.9 | | 5.0 | | 7.6 | | 9.7 | | 9.8 | | 0.0 | 0.0 | | 10.0 | | 8.4 | | 10.0 | | 10.0 | | 10.0 | | 125.4 | |
| **Components of the PHDI-C ^e^** | | | | **Nuts & peanuts** | | **Legumes** | | **Fruits** | | **Vegetables** | | **ReV ratio** | | **DGV ratio** | | **Cereals** | **WC ratio** | **Tubers & potatoes** | | **Dairy products** | | **Eggs and white meats** | | | | | **Vegetable oils** | **Palm oil** | **Red meats** | | **Animal fats** | | **Added sugars** | | **Total score**  **(points)** | |
| **Recommended percentages of total caloric intake ^e^**  **Optimal value (range)** | | | | 11.6  (0.0, 100) | | 11.3  (0.0, 100) | | 5.0  (0.0, 100) | | 3.1  (0.0, 100) | | 38.5  (0.0, 100) | | 29.5  (0.0, 100) | | 30.0 (0.0, 60.0) | 75.0 (0.0, 100) | 1.6  (0.0, 3.1) | | 6.1  (0.0, 12.2) | | 6.2  (0.0, 12.2) | | | | | 14.1  (0.0 28.3) | 0.0  (0.0, 2.4) | 0.0  (0.0, 2.4) | | 0.0  (0.0, 1.4) | | 0.0  (0.0, 4.8) | | 150 | |
| **Actual percentages of total caloric intake** | | | | 18.3 | | 11.6 | | 6.9 | | 3.2 | | 37.5 | | 29.7 | | 32.4 | 76.0 | 1.5 | | 6.0 | | 6.1 | | | | | 13.9 | 0.0 | 0.0 | | 0.0 | | 0.0 | | - | |
| **PHDI-C score ^e^** | | | | 10.0 | | 10.0 | | 10.0 | | 10.0 | | 4.9 | | 5.0 | | 9.2 | 9.6 | 9.7 | | 9.8 | | 9.9 | | | | | 9.8 | 10.0 | 10.0 | | 10.0 | | 10.0 | | 147.9 | |
| Abbreviations: USDA, United States Department of Agriculture; SAFA, saturated fatty acids; MUFAs, Monounsaturated fatty acids; PUFAs, Polyunsaturated fatty acids; PHDI, Planetary Health Diet Index; PHDI-C, Planetary Health Diet Index for children and adolescents; ReV ratio, red and orange vegetables ratio; DGV ratio, dark green vegetables ratio; WC ratio; whole cereals ratio.  ^a^ Each food item is color-coded with its corresponding index component.  ^b^ Food items' nutritional composition was obtained from the USDA National Nutrient Database (1).  ^c^ The caloric requirement of 2,503 kcal/day corresponds to the caloric requirement of a 56.7 kg girl aged 18 years whose level of physical activity is moderate to high (2). Macronutrient requirements were calculated based on acceptable macronutrient distribution ranges (3). Micronutrient requirements were defined based on Recommended Dietary Allowances or Average Intakes for children aged 14-18-year-old (3, 4).  ^d^ Components and scores correspond to the PHDI developed and validated by Cacau et al (5). Each component is associated to a recommended range of total caloric intake expressed as percentage of total calories, except for the ratio components which are expressed as percentage of total calories from vegetables. All components can score between 0 to 10 points, except for the ratio components which can score between 0 to 5 points, resulting in a total score of 150 points (5).  ^e^ Components and scores correspond to the PHDI-C proposed in this study. Each component is associated to a recommended range of total caloric intake expressed as percentage of total calories, except for the DGV ratio and ReV ratio components which are expressed as percentage of total calories from vegetables, and the WC ratio which is expressed as percentage of total calories from cereals. All components can score between 0 to 10 points, except for the DGV and ReV ratio components which can score between 0 to 5 points, resulting in a total score of 150 points. The formula to calculate the score for each component is provided in Table 2. | | | | | | | | | | | | | | | | | | | | | | | | | | | | | | | | | | | | |

# Supplemental figure 2. Decision tree to guide the allocation of calories from reported food items into PHDI-C components.

| Does the food item contribute calories to a single component of the PHDI-C^b^?  Is the food item made from a single ingredient or a single ingredient plus one or more food additives^a^?  Yes  No  Allocate the food item's calories directly into its corresponding index component (see Supplemental table 6)  No  Do not allocate the food item's calories into any index component  No  Is the food item made from a combination of ingredients that do not contribute calories to any component of the PHDI-C^b^?  Does the food item contain ingredients that contribute calories to a single component of the PHDI-C^b^ plus other ingredients that are not relevant sources of calories such as water, tea, coffee, baking powder, baking soda, yeast, salt, herbs, spices, or other food additives^a^?  Disaggregate food item into ingredients and allocate the calories from each caloric source into its corresponding index component (see Supplemental table 7)  Is the food item made from two or more ingredients that contribute calories to multiple components of the PHDI-C^b^?  Yes  Yes  No  Yes  Yes  Does the food item contain ingredients that contribute calories to a single component of the PHDI-C^b^ plus other relevant sources of calories such as cocoa powder?  No  Yes |
| --- |
|  |

Abbreviations: PHDI-C, Planetary Health Diet Index for children and adolescents.

^a^ Food additives are substances added to foods to maintain or improve its safety, freshness, taste, texture, or appearance. Examples include non-caloric sweeteners, salt, herbs, spices, vinegar, vitamins, minerals, stabilisers, antioxidants, antimicrobials, or other additives used to preserve the properties of the original food, carbonating, firming, bulking and anti-bulking, de-foaming, anti-caking and glazing agents, emulsifiers, sequestrants and humectants or other processing aids, colour additives, flavours, flavour enhancers or other cosmetic or sensory intensifying additives.

^b^ Components of the PHDI-C include: nuts and peanuts (including seeds); legumes (including soy); fruits; vegetables; cereals (whole and refined); eggs and white meats (i.e., chicken and other poultry, fish and seafood, and white processed meats); tubers and potatoes; dairy products; vegetable oils; palm oil; red meats (i.e., beef, lamb, pork and processed red meat); animal fats (i.e., lard, butter, cream); added sugars. Examples of food items included in each component are described in Supplemental table 6.

# Supplemental table 6. Food items included in each component of the Planetary Health Diet Index for children and adolescents (PHDI-C).

| **Component** | **Included food items** |
| --- | --- |
| Nuts and peanuts | Nuts, almonds, pistachios, macadamia, peanuts, and seeds. Includes foods that contain nuts (e.g., peanut butter, Nutella, and chocolate bars). |
| Legumes | Beans, pulses, lentils, chickpeas, peas, and soybeans. Includes foods that contain soy (e.g., soy milks, tofu, textured soy protein). |
| Fruits | All fruits, including fruits used in juices, nectars, compotes, and jams. |
| Vegetables | All vegetables, except herbs, tubers, and corn. Includes canned, pickled and fermented vegetables (e.g., palm hearts, pickles, sauerkraut), as well as food that contain vegetables (e.g., baby food). |
| Dark green vegetables ratio | Broccoli, arugula, cabbage, brussels sprouts, chard, chicory, artichoke, watercress, spinach. Includes food that contain vegetables (e.g., baby food). |
| Red and orange vegetables ratio | Tomatoes, beetroots, red bell peppers, red cabbage, carrots, and pumpkins. Includes food that contain vegetables (e.g., baby food). |
| Cereals | White and brown rice, quinoa, corn, rye, oats, flour. Includes foods that contain whole and/or refined cereals (e.g., bread, crackers, pasta, cookies, breakfast cereals, baby cereals, flavoured powders, commercial soups, stock powders, dressings and sauces). |
| Whole cereals ratio | Brown rice, quinoa, corn, rye, oats, wholemeal flour. Includes foods that contain whole grains (e.g., wholemeal bread, wholemeal crackers, wholemeal pasta, wholemeal cookies, oatmeal cookies, wholemeal breakfast cereals, and wholemeal baby cereals). |
| Tubers and potatoes | All types of potatoes. Includes foods that contain potatoes (e.g., baby food, French fries, potato chips) |
| Dairy products | Milk, milk drinks, yoghurt, and cheese. Includes products that contain milk (e.g., dairy-based ice creams and desserts, bakery products, caramel, nutritional supplements) |
| Eggs and white meats | Eggs, chicken, poultry, fish, and seafood. Includes processed white meats (e.g., pate, sausage, ham, nuggets, burgers, crab sticks), canned fish and seafood, and foods that contain white meats (e.g., baby food). |
| Vegetable oils | Vegetable oils (excluding palm oil) used in culinary preparations and manufactured foods (e.g., breakfast cereals, bakery products, baby food, infant formulas, deep-fried foods, industrialized soups, etc.). Avocados and olives were also included in this component. |
| Palm oil | Palm oil used in manufactured foods (e.g., chocolate bars, cereal bars, breakfast cereals, sweet bakery products, cookies). |
| Red meats | Beef, lamb, and pork, including processed red meats (e.g., sausage, ham, salami, burgers). |
| Animal fats | Lard, butter, and other dairy fats (e.g., cream). Includes foods that contain animal fat (e.g., sweet and savory bakery products, bread, milk-based desserts, cookies) |
| Added sugars | All sweeteners, including white or brown sugar, honey, syrups and other sugars used in culinary preparations and manufactured foods and beverages (e.g., milk, milk drinks, yoghurts, infant formulas, breakfast cereals, candy bars, cereal bars, chocolate bars, sweet bakery products, cookies, etc.). |
|  | |

# Supplemental table 7. Food disaggregation methodology for allocating calories from composite foods into multiple components of the Planetary Health Diet Index for children and adolescents (PHDI-C).

| **Group of food items** | **Main calorie sources ^a^** | **Allocation of calories to each index component ^b^** | **Rationale and assumptions** |
| --- | --- | --- | --- |
| Breakfast cereals, baby cereals, cereal bars, sweet biscuits, sweet snacks | Added sugars | - Added sugars component = 100% of calories from declared sugars if the food item included added sugars in the ingredients list. | - In line with PAHO’s criteria for estimating free sugars,^d^ we assumed all calories from declared sugars came from free sugars added as sweeteners. |
|  | Added fats | - Palm oil component = 100% of calories from declared fat if the food item’s main source of fat was palm oil.   OR   - Vegetable oils component = 100% of calories from declared fat if the food item’s main source of fat was vegetable oils.   OR   - Animal fats component = 100% of calories from declared fat if the food item’s main source of fat was animal fats. | - Cereals provide minimal amounts of fat. Therefore, we assumed all calories from declared fat came from the main source of fat in the ingredients list (i.e., palm oil, or vegetable oils, or animal fats). - If an ingredients list was not available, we assumed all calories from declared fats came from palm oil because this was the most common source of fat in these food items (70% contained palm oil). |
|  | Cereals | - Cereals component = remaining calories, after discounting calories from added sugars and added fats if the food item’s main ingredient ^c^ was cereals.   - Whole cereals ratio component = remaining calories after discounting calories from added sugars and added fats if the food item’s main ingredient ^c^ was whole cereals. | - We assumed all remaining calories after discounting calories from added sugars and added fats came from cereals if the food item’s main ingredient ^c^ was cereals.   - If the food item’s main ingredient ^c^ was whole cereals or a combination of whole and refined cereals and provided more than 2.5g of fibre per serving.^e^, we assumed all remaining calories after discounting calories from added sugars and added fats came from whole cereals.   - If the food item’s main ingredient ^c^ was refined cereals or a combination of whole and refined cereals but the food item provided less than 2.5g of fibre per serving, no calories were allocated to the whole cereals ratio component. |
| Candy bars, sweet snacks, nougat, chocolates, and confectionery | Added sugars | - Added sugars component = 100% of calories from declared sugars if the food item included added sugars in the ingredients list. | - In line with PAHO’s criteria for estimating free sugars,^d^ we assumed all calories from declared sugars came from free sugars added as sweeteners. - Some food items in this group included fruit or milk in the first five ingredients, however, their caloric contribution represented less than 10% of calories from declared sugars. Therefore, we assumed all calories from declared sugars came from free sugars added as sweeteners. |
|  | Nuts | - Nuts and peanuts component = 18% of total calories if the food item included nuts in the first five ingredients. | - Some food items included 13-26% of nuts in the ingredients list, which on average accounted for 18% of total calories. |
|  | Added fats | - Palm oil component = 100% of calories from declared fat if the food item’s main source of fat was palm oil and did not include nuts in the first five ingredients OR 75% of calories from declared fat if the food item’s main source of fat was palm oil and nuts were included in the first five ingredients.   OR   - Vegetable oils component = 100% of calories from declared fat if the food item’s main source of fat was vegetable oils and the item did not include nuts in the first five ingredients OR 75% of calories from declared fat if the food item’s main source of fat was vegetable oils and the item included nuts in the first five ingredients. | - If the food item did not contain nuts, we assumed all calories from declared fat came from the main source of fat in the ingredients list (i.e., palm oil or vegetable oils). - If the food item contained nuts, we assumed 75% of calories from declared fat came from the main source of fat in the ingredients list (i.e., palm oil or vegetable oils). This decision was based on the food items’ content of nuts (13-26%), which on average accounted for 25% of calories from declared fats. - If an ingredients list was not available, we assumed all calories from declared fat came from palm oil because this was the most common source of fat among these food items (68% contained palm oil). |
|  | Cereals | - Cereals component = remaining calories, after discounting calories from added sugars, added fats, nuts if the food item included cereals in the ingredients list | - We assumed all remaining calories after discounting calories from added sugars and added fats came from cereals if the food item included cereals in the ingredients list. - All food items in this group contained refined cereals instead of whole cereals. - We did not account for the caloric contribution of fruit or milk in this group of food items because they represented less than 10% of total calories. - We did not account for the caloric contribution of cocoa because it was not included in the index. |
| Sweet baked products (e.g., cakes, cupcakes, muffins, donuts, tarts, lemon pie) | Added sugars | - Added sugars component = 100% of calories from declared sugars if the food item included added sugars in the ingredients list. | - In line with PAHO’s criteria for estimating free sugars,^d^ we assumed all calories from declared sugars came from free sugars added as sweeteners. - Some food items in this group included fruit or milk in the first five ingredients, however, their caloric contribution represented less than 10% of calories from declared sugars. Therefore, we assumed all calories from declared sugars came from free sugars added as sweeteners. |
|  | Added fats | - Palm oil component = 100% of calories from declared fat if the food item’s main source of fat was palm oil.   OR   - Vegetable oils component = 100% of calories from declared fat if the food item’s main source of fat was vegetable oils.   OR   - Animal fats component = 100% of calories from declared fat if the food item’s main source of fat was animal fats. | - Most food items in this group included secondary sources of fat such as eggs and milk in the first five ingredients. The caloric contribution of these sources represented less than 10% of calories from declared fat. Therefore, we assumed all calories from declared fat came from the main source of fat in the ingredients list (i.e., palm oil, or vegetable oils, or animal fats). - If an ingredients list was not available, we assumed all calories from declared fat came from palm oil because this was the most common source of fat among these food items. |
|  | Cereals | - Cereals component = remaining calories, after discounting calories from added sugars and added fats if the food item’s main ingredient ^c^ was cereals. | - We assumed all remaining calories after discounting calories from added sugars and added fats came from cereals if the food item’s main ingredient ^c^ was cereals. - All food items in this group contained refined cereals instead of whole cereals. - We did not account for the caloric contribution of fruit, milk, and eggs in this group of food items because they represented less than 10% of total calories. |
| Bread, pasta, pizza dough, tortillas, savoury crackers, savoury snacks | Added fats | - Palm oil component = 100% of calories from declared fat if the food item’s main source of fat was palm oil.   OR   - Vegetable oils component = 100% of calories from declared fat if the food item’s main source of fat was vegetable oils.   OR   - Animal fats component = 100% of calories from declared fat if the food item’s main source of fat was animal fats. | - Cereals provide minimal amounts of fat. Therefore, we assumed all calories from declared fat came from the main source of fat in the ingredients list (i.e., palm oil, or vegetable oils, or animal fats). - If an ingredients list was not available, we used standard recipes to determine the most common source of fat among these food items. |
|  | Cereals | - Cereals component = remaining calories, after discounting calories from added fats if the food item’s main ingredient ^c^ was cereals.   - Whole cereals ratio component = remaining calories after discounting calories from added fats if the food item’s main ingredient ^c^ was whole cereals. | - We assumed all remaining calories after discounting calories from added fats came from cereals if the food item’s main ingredient ^c^ was cereals.   - If the food item’s main ingredient ^c^ was whole cereals or a combination of whole and refined cereals and provided more than 2.5g of fibre per serving.^e^, we assumed all remaining calories after discounting calories from added fats came from whole cereals.   - If the food item’s main ingredient ^c^ was refined cereals or a combination of whole and refined cereals but the food item provided less than 2.5g of fibre per serving, no calories were allocated to the whole cereals ratio component. |
| Pizzas | Cheese | - Dairy products component = 32% of total calories if the food item included cheese in the first five ingredients. | - The standard recipe for napolitana pizza, the most common food item in this group, contains 18% cheese, which accounts for 32% of total calories. |
|  | Red meat | - Red meats component = 24% of total calories if the food item included red meat in the first five ingredients. | - The standard recipe for napolitana pizza, the most common food item in this group, contains 20% pork ham, which accounts for 24% of total calories. |
|  | Cereals | - Cereals component = remaining calories, after discounting calories from cheese and red meat if the food item’s main ingredient ^c^ was cereals. | - We assumed all remaining calories after discounting calories from dairy products and red meats came from cereals if the food item’s main ingredient ^c^ was cereals. - All food items in this group contained refined cereals instead of whole cereals. - No calories were allocated for other ingredients such as vegetables because they represented less than 10% of total calories in these food items. |
| Empanadas | Red meat | - Red meats component = 13% of total calories if the food item included red meat in the first five ingredients. | - The standard recipe for meat empanadas contains 16% red meat/pork, which accounts for 13% of total calories. |
|  | Cheese | - Dairy products component = 27% of total calories if the food item included cheese in the first five ingredients. | - The standard recipe for cheese empanadas contains 30% cheese, which accounts for 27% of total calories. |
|  | Added fats | - Vegetable oils component = 18% of total calories if the food item included vegetable oils in the ingredients list.   AND   - Animal fats component = 18% of total calories if the food item included animal fats in the ingredients list. | - The standard recipe for empanadas contains 6% vegetable oils, which accounts for 18% of total calories, and 6% animal fats, which accounts for 18% of total calories. |
|  | Cereals | - Cereals component = remaining calories, after discounting calories from red meat, cheese and added fats if the food item’s main ingredient ^c^ was cereals. | - We assumed all remaining calories after discounting calories from dairy products, red meats, and added fats came from cereals if the food item’s main ingredient ^c^ was cereals. - All food items in this group contained refined cereals instead of whole cereals. - No calories were allocated for other ingredients such as vegetables and eggs that may be included in these food items because they represented less than 10% of total calories. |
| Hamburgers, hot dogs, and other sandwiches | Red meat | - Red meats component = 30% of total calories if the food item included beef or pork in the first five ingredients.   OR   - Eggs and white meats component = 30% of total calories if the food item included chicken or other poultry in the first five ingredients. | - The standard recipe for burgers, the most common food item in this group, contains 30% meat (beef, pork, or chicken), which accounts for 30% of total calories. |
|  | Cheese | - Dairy products component = 18% of total calories if the food item included cheese in the first five ingredients. | - The standard recipe for burgers, the most common food item in this group, contains 11% cheese, which accounts for 18% of total calories. |
|  | Cereals | - Cereals component = remaining calories, after discounting calories from red meat and cheese if the food item’s main ingredient ^c^ was cereals. | - We assumed all remaining calories after discounting calories from dairy products and red meats came from cereals if the food item’s main ingredient ^c^ was cereals. - All food items in this group contained refined cereals instead of whole cereals. - No calories were allocated for other ingredients such as added fats and vegetables that may be included in these food items because they represented less than 10% of total calories. |
| Meat substitutes (e.g., soy burgers) | Added fats | - Vegetable oils component = 100% of calories from declared fat if the food item included vegetable oils in the ingredients list. | - Soy usually provides minimal amounts of fat. Therefore, we assumed all calories from declared fat were from the main source of fat included in the ingredients list (i.e., vegetable oils). |
|  | Legumes | - Legumes component = remaining calories after discounting calories from vegetable oils if the food item’s main ingredient ^c^ was soy. | - We assumed all remaining calories after discounting calories from vegetable oils came from legumes if the food item’s main ingredient ^c^ was soy. - There were no plant-based meat substitutes other than soy-based meat substitutes in this database. |
| Potato chips, French fries | Added fats | - Vegetable oils component = 100% of calories from declared fat if the food item included vegetable oils in the ingredients list. | - Potatoes usually provide minimal amounts of fats. Therefore, we assumed 100% of declared fat came from the main source of fat in the ingredients list (i.e., vegetable oils). |
|  | Potatoes | - Tubers and potatoes component = remaining calories after discounting calories from vegetable oils if the food item’s main ingredient ^c^ was potatoes. | - We assumed all remaining calories after discounting calories from vegetable oils came from potatoes because they were the main ingredient ^c^ of these food items. |
| Baby food | Meat | - Red meats component = 22% of total calories if the food item included red meat in the 5 first five ingredients.   OR   - Eggs and white meats component = 22% of total calories if the food item included chicken in the first five ingredients. | - The standard recipe for baby food contains 15% meat (i.e., beef or chicken), which accounts for 22% of total calories. |
|  | Potatoes | - Tubers and potatoes component = 28% of total calories if the food item included potatoes in the first five ingredients. | - The standard recipe for baby food contains 25% potatoes, which accounts for 28% of total calories. |
|  | Vegetables | - Vegetables component = 14% of total calories if the food item included vegetables in the first five ingredients.   - DGVratio component = 3% of total calories if the food item included dark green vegetables in the first five ingredients   - ReV ratio component = 11% of total calories if the food item included red vegetables in the first five ingredients | - The standard recipe for baby food contains 30% red vegetables and 7.5% dark green vegetables, which accounts for 11% and 3% of total calories, respectively. |
|  | Added fats | - Vegetable oils component = 29% of total calories if the food item included vegetable oils in the ingredients list. | - The standard recipe for baby food contains 2.5% vegetable oils, which accounts for 29% of total calories. |
|  | Cereals | - Cereals component = remaining calories, after discounting calories from meat, potatoes, vegetables, and added fats if the food item included cereals in the ingredients list | - We assumed all remaining calories, after discounting calories from meat, potatoes, vegetables, and added fats came from cereals because the standard recipe for baby food contains refined cereals (i.e., rice or pasta). |
| Infant formulas | Added sugars | - Added sugars component = 50% of calories from declared sugars if the food item included added sugars in the ingredients list and was made out of milk. | - In line with PAHO’s criteria for estimating free sugars,^d^ we assumed 50% of calories from declared sugars came from free sugars added as sweeteners if the food item was made out of milk. |
|  | Added fats | - Vegetable oils component = 100% of calories from declared fat if the food item included vegetable oils in the ingredients list. | - Most infant formulas were made out of low-fat milk and contained vegetable oils. Therefore, we assumed all calories from declared fat came from vegetable oils. |
|  | Milk | - Dairy products component = remaining calories after discounting calories from added sugars and added fats if the food item’s main ingredient ^c^ was milk. | - We assumed all remaining calories after discounting calories from added sugars and added fats came from dairy because milk was the main ingredient ^c^ of these food items. - No calories were allocated for refined cereals used as thickeners in these food items because they are not included in the index. |
| Milk, milk drinks, and yoghurts without cereals | Added sugars | - Added sugars component = 50% of calories from declared sugars if the food item included added sugars in the ingredients list and was made out of milk. | - In line with PAHO’s criteria for estimating free sugars,^d^ we assumed 50% of calories from declared sugars came from free sugars added as sweeteners. |
|  | Milk | - Dairy products = remaining calories after discounting calories from added sugars if the food item’s main ingredient ^c^ was milk. | - We assumed all remaining calories after discounting calories from added sugars came from dairy because milk was the main ingredient ^c^ of these food items. |
| Yoghurts with cereals | Added sugars | - Added sugars component = 60% of calories from declared sugars if the food item included added sugars in the ingredients list, as well as sugary cereals and was made out of milk. | - The ingredients list of food items in this group included 93% sugar-sweetened yoghurt + 7% sugary cereals. - In line with PAHO’s criteria for estimating free sugars,^d^ we assumed that:   - 50% of declared sugars in yoghurt came from free sugars added as sweeteners. This accounted for 40% of calories from declared sugars.   - 100% of sugars in cereals came from free sugars added as sweeteners. This accounted for 20% of calories from declared sugars. - Therefore, we assumed 60% of calories from declared sugars were from free sugars added as sweeteners. |
|  | Milk | - Dairy products component = 75% of the remaining calories after discounting calories from added sugars if the food item’s main ingredient ^c^ was milk and included sugary cereals in the ingredients list. | - The ingredient list of food items in this group included 93% sugar-sweetened yoghurt, which accounted for 75% of the remaining calories. Therefore, we assumed milk accounted for 75% of remaining calories after discounting calories from added sugars. |
|  | Cereals | - Cereals component = remaining calories, after discounting calories from added sugars and milk if the food item included cereals in the ingredients list. | - We assumed all remaining calories, after discounting calories from added sugars and milk came from cereals if the food item included cereals in the ingredients list. - All food items in this group contained refined cereals instead of whole cereals. |
| Milk-based desserts without cereals (e.g., flan, mousse) | Added sugars | - Added sugars component = 75% of calories from declared sugars if the food item included added sugars in the ingredients list and milk in the first five ingredients OR 100% of calories from declared sugars if the food item included added sugars in the ingredients list and did not include milk in the first five ingredients. | - In line with PAHO’s criteria for estimating free sugars,^d^ we assumed 75% of calories from declared sugars came from free sugars added as sweeteners if the food item contained milk. - There were some food items that did not contain milk, and therefore, we assumed 100% of calories from declared sugars came from free sugars added as sweeteners. |
|  | Added fats | - Palm oil component = 75% of calories from declared fat if the food item’s main source of fat was palm oil and included milk in the first five ingredients OR 100% of calories from declared fat if the food item’s main source of fat was palm oil and did not include milk in the first five ingredients.   OR   - Animal fats component = 75% of calories from declared fat if the food item’s main source of fat was animal fats and included milk in the first five ingredients. | - Milk-based desserts without added fats had 75% less fat than milk-based desserts with added fats. Therefore, we assumed 75% of declared fat came from the main source of fat in the ingredients list (i.e., palm oil or animal fats). - There were some food items that did not contain milk, and therefore, we assumed 100% of calories from declared fat came from palm oil, which was the only source of fat included in the ingredients list. - If the ingredients list was not available, we assumed all calories from declared fat came from milk, because this was the food item’s main ingredient ^c^. |
|  | Milk | - Dairy products component = remaining calories after discounting calories from added sugars and added fats if the food item included milk but not cereals in the first five ingredients.   OR   - Remaining calories, after discounting calories from added sugars and added fats, were not allocated into an index component. | - We assumed all remaining calories after discounting calories from added sugars and added fats came from dairy products if milk was the main ingredient ^c^ of these food items. - If milk was not included in the food item’s first five ingredients, no calories were allocated to an index component. - No calories were allocated for refined cereals used as thickeners in these food items because they are not included in the index. |
| Milk-based desserts with cereals (e.g., rice pudding, semolina pudding) | Added sugars | - Added sugars component = 75% of calories from declared sugars if the food item included added sugars in the ingredients list and included milk in the first five ingredients. | - In line with PAHO’s criteria for estimating free sugars,^d^ we assumed 75% of calories from declared sugars came from free sugars added as sweeteners if the food item contained milk. |
|  | Milk | - Dairy products component = 20% of total calories if the food item contained milk and cereals in the first five ingredients. | - The standard recipe for semolina pudding, the most common food item in this group, contains 60% milk, which accounts for 20% of total calories. |
|  | Cereals | - Cereals component = remaining calories, after discounting calories from added sugars and milk if the food item included cereals in the ingredients list. | - We assumed all remaining calories, after discounting calories from added sugars and milk came from cereals because the standard recipe for semolina pudding, the most common food item in this group, contains 30% refined cereals |
| Milk-based ice creams, Coffee-based drinks | Added sugars | - Added sugars component = 75% of calories from declared sugars if the food item included added sugars in the ingredients list and contained milk in the first five ingredients. | - In line with PAHO’s criteria for estimating free sugars,^d^ we assumed 75% of calories from declared sugars came from free sugars added as sweeteners if the food item contained milk. |
|  | Added fats | - Palm oil component = 100% of calories from declared fat if the food item’s main source of fat was palm oil.   OR   - Vegetable oils component = 100% of calories from declared fat if the food item’s main source of fat was vegetable oils.   OR   - Animal fats component = 100% of calories from declared fat if the food item’s main source of fat was animal fats. | - Most milk-based ice creams and coffee-based drinks contained low-fat milk. Therefore, we assumed all calories from declared fat came from the main source of fat in the ingredients list (i.e., palm oil, or vegetable oils, or animal fats). - If the ingredients list was not available, we assumed all calories from declared fat came from palm oil because this was the most common source of fat among these food items (93% of products contained palm oil). |
|  | Milk | - Dairy products component = remaining calories after discounting calories from added sugars and added fats if the food item contained milk in the first five ingredients. | - We assumed all remaining calories after discounting calories from added sugars and added fats came from dairy because milk was the main ingredient ^c^ of these food items. |
| Milk substitutes (i.e., soy milk, rice milk) | Added sugars | - Added sugars component = 100% of calories from declared sugars if the food item included added sugars in the ingredients list. | - In line with PAHO’s criteria for estimating free sugars,^d^ we assumed all calories from declared sugars came from free sugars added as sweeteners. |
|  | Added fats | - Vegetable oils component = 100% of calories from declared fat if the food item included vegetable oils in the ingredients list. | - Cereals and legumes usually provide minimal amounts of fats. Therefore, we assumed 100% of declared fat came from the vegetable oils included in the ingredients list. |
|  | Cereals or legumes | - Cereals component = remaining calories after discounting calories from added sugars and added fats if the food item’s main ingredient ^c^ was cereals.   - Whole cereals ratio component = remaining calories after discounting calories from added sugars and added fats if the food item’s main ingredient ^c^ was whole cereals.   OR   - Legumes component = remaining calories after discounting calories from added sugars and added fats if the food item’s main ingredient ^c^ was legumes. | - If the food item’s main ingredient ^c^ was cereals, we assumed all remaining calories after discounting calories from added sugars and added fats came from cereals.   - If the food item’s main ingredient ^c^ was whole cereals, we assumed all remaining calories after discounting calories from added sugars and added fats came from whole cereals.   - If the food item’s main ingredient ^c^ was refined cereals, no calories were allocated to the whole cereals ratio component. - If the food item’s main ingredient ^c^ was legumes (i.e., soy), we assumed all remaining calories after discounting calories from added sugars and added fats came from legumes. - There were no nut-based milk substitutes in the database. |
| Natural fruit juice, fruit compotes, fruits in syrup | Added sugars | - Added sugars component = 50% of calories from declared sugars if the food item included added sugars in the ingredients list and was made out of fruit. | - In line with PAHO’s criteria for estimating free sugars,^d^ we assumed 50% of calories from declared sugars came from free sugars added as sweeteners. |
|  | Fruit | - Fruits component = remaining calories after discounting calories from added sugars if the food item’s main ingredient ^c^ was fruit. | - We assumed all remaining calories after discounting calories from added sugars were from fruit because this was the main ingredient ^c^ of these food items. |
| Nectars, jams, fruit-based water ice creams, fruit sorbets | Added sugars | - Added sugars component = 75% of calories from declared sugars if the food item included added sugars in the ingredients list and contained fruit in the first five ingredients. | - In line with PAHO’s criteria for estimating free sugars,^d^ we assumed 75% of calories from declared sugars came from free sugars added as sweeteners. This is because nectars contain less than 50% of fruit. |
|  | Fruit | - Fruits component = remaining calories after discounting calories from added sugars if the food item included fruit in the first five ingredients. | - We assumed all remaining calories after discounting calories from added sugars came from fruit because this was the main ingredient ^c^ of these food items. |
| Jellies, syrups, flavoured powders (e.g., Milo, Nesquik), confetti | Added sugars | - Added sugars component = 100% of calories from declared sugars if the food item included added sugars in the ingredients list. | - In line with PAHO’s criteria for estimating free sugars,^d^ we assumed all calories from declared sugars came from free sugars added as sweeteners. - Some of these food items contained fruit or milk in the first five ingredients, however, their caloric contribution represented less than 10% of calories from declared sugars. Therefore, we assumed all calories from declared sugars came from free sugars added as sweeteners. |
|  | Cereals | - Cereals component = remaining calories, after discounting calories from added sugars if the food item included cereals in the ingredients list | - We assumed all remaining calories, after discounting calories from added sugars came from cereals if the food item included cereals in the ingredients list. - No calories were allocated for cocoa powder because it is not included in the index. |
| Sweet spreads (i.e., caramel) | Added sugars | - Added sugars component = 75% of calories from declared sugars if the food item included added sugars in the ingredients list and milk in the first five ingredients. | - In line with PAHO’s nutrient profile criteria for estimating free sugars,^d^ we assumed 75% of calories from declared sugars came from free sugars if the food item contained milk. |
|  | Milk | - Dairy products component = remaining calories after discounting calories from added sugars if the food item contained milk in the first five ingredients. | - We assumed all remaining calories after discounting calories from declared added sugars came from dairy because milk was the main ingredient ^c^ of these food items. |
| Nutella | Added sugars | - Added sugars component = 100% of calories from declared sugars if the food item included added sugars in the ingredients list. | - In line with PAHO’s criteria for estimating free sugars,^d^ we assumed all calories from declared sugars came from free sugars added as sweeteners. |
|  | Added fats | - Palm oil component = 75% of calories from declared fat if the food item contained palm oil & nuts in the first five ingredients. | - We assumed 75% of declared fat came from palm oil and that the rest came from nuts. This decision was based on the food items’ content of nuts (13%), which approximately accounted for 25% of declared fat. |
|  | Nuts | - Nuts and peanuts component = 14% of total calories if the food item contained nuts in the first five ingredients. | - According to the ingredients list, these food items contained 13% of nuts, which accounts for 14% of total calories. |
|  | Cocoa powder | - Remaining calories, after discounting calories from added sugars, added fats, and nuts, were not allocated into an index component. | - We did not account for the caloric contribution of cocoa powder because it is not included in the index. |
| Peanut butter, caramelized peanuts and nuts | Added sugars | - Added sugars component = 100% of calories from declared sugars if the food item included added sugars in the ingredients list. | - In line with PAHO’s nutrient profile criteria for estimating free sugars,^d^ we assumed all calories from declared sugars came from free sugars added as sweeteners. |
|  | Nuts | - Nuts and peanuts component = remaining calories after discounting calories from added sugars if the food item’s main ingredient ^c^ was nuts. | - We did not account for the caloric contribution of vegetable oils in nut-based products such as peanut butter, salted nuts or caramelized nuts because the main source of fat in these food items was nuts. Therefore, we assumed all remaining calories after discounting calories from added sugars came from nuts. |
| Commercial soups, stock powders, tomato sauce, Italian sauce, ketchup, mayonnaise, mustard, and other dressings | Added sugars | - Added sugars component = 100% of calories from declared sugars if the food item included added sugars in the ingredients list. | - In line with PAHO’s criteria for estimating free sugars,^d^ we assumed all calories from declared sugars came from free sugars added as sweeteners. |
|  | Added fats | - Palm oil component = 100% of calories from declared fat if the food item’s main source of fat was palm oil.   OR   - Vegetable oils component = 100% of calories from declared fat if the food item’s main source of fat was vegetable oils. | - We assumed all calories from declared fat came from the main source of fat in the ingredients list (i.e., palm oil or vegetable oils). - If the ingredients list was not available, we assumed all calories from declared fat came from the most common source of fat in the ingredients list of similar products (i.e., palm oil among commercial soups, vegetable oils among mayonnaise). - We did not account for the content of fat provided by eggs in the case of mayonnaise, and low-fat milk in the case of commercial soups because they represented less than 10% of calories from declared fat. |
|  | Cereals | - Cereals component = remaining calories, after discounting calories from added sugars and added fats if the food item included cereals in the ingredients list. | - We assumed all remaining calories, after discounting calories from added sugars and added fats came from cereals if the food item included cereals in the ingredients list (e.g., corn starch). - No calories were allocated for other ingredients such as vegetables, meat, eggs, or milk that may be included in these food items because their content represented less than 10% of total calories. |
| Nutritional supplements (e.g., Pediasure) | Added sugars | - Added sugars component = 100% of calories from declared sugars if the food item included added sugars in the ingredients list. | - Nutritional supplements only contain milk protein, not whole milk. Therefore, in line with PAHO’s criteria for estimating free sugars,^d^ we assumed all calories from declared sugars came from free sugars added as sweeteners. |
|  | Added fats | - Vegetable oils component = 100% of calories from declared fat if the food item included vegetable oils in the ingredients list. | - We assumed all calories from declared fat came from the main source of fat in the ingredients list (i.e., vegetable oils). |
|  | Milk | - Dairy products component = 100% of calories from declared protein if the food item contained milk in the first five ingredients. | - We assumed all calories from declared protein came from dairy because this was the only source of protein declared in the ingredients list. |
|  | Cereals | - Cereals component = remaining calories, after discounting calories from added sugars, added fats, and milk if the food item included cereals in the ingredients list. | - We assumed all remaining calories, after discounting calories from added sugars, added fats, and milk came from cereals if the food item included cereals in the ingredients list (e.g., maltodextrin). |
| Abbreviations: PAHO, Pan American Health Organization; ReV ratio, red and orange vegetables ratio; DGV ratio, dark green vegetables ratio.  ^a^ We used the list of ingredients, and in some cases, standard recipes previously developed by dietitians at the Institute of Nutrition and Food Technology, to determine which were the main sources of calories in each food item. Standard recipes were only used for processed foods when the ingredients list was not available. In the case of ultra-processed foods, we used ingredients list from similar products.  ^b^ Nutritional information panels were used to allocate calories from each calorie source into the corresponding index components. We did not account for the caloric contribution of ingredients that provided less than 10% of total calories.  ^c^ A food item’s main ingredient is the one that is essential for its characterization. For example, refined cereals are the main ingredient of breakfast cereals; milk is the main ingredient of dairy products.  ^d^ According to PAHO (6), free sugars are defined as monosaccharides and disaccharides added to foods and beverages by the manufacturer, cook, and/or consumer plus sugars that are naturally present in honey, syrups and juices.  ^e^ According to the Chile’s Food Sanitary Code (7), a food item is a good source of fibre when it provides more than 2.5g of fibre per serving. We used this criterion to assess whether food items contained a significant amount of whole cereals when they contained a mix of whole and refined cereals. | | | |

# References

1. United States Department of Agriculture (USDA), Agricultural Research Service. FoodData Central 2019 [Available from: <https://ndb.nal.usda.gov/ndb/>.

2. FAO, WHO, UNU. Human energy requirements: Report of a Joint FAO/WHO/UNU Expert Consultation. Rome, 17-24 October 2001. Rome: FAO; 2004. Report No.: 92-5-105212-3ISSN 1813-3932.

3. Institute of Medicine. Dietary Reference Intakes: The Essential Guide to Nutrient Requirements. Washington, DC: IOM; 2006 2006-08-29.

4. Ross A, Manson J, Abrams S, Aloia J, Brannon P, Clinton S, et al. The 2011 report on dietary reference intakes for calcium and vitamin D from the Institute of Medicine: what clinicians need to know. J Clin Endocrinol Metab. 2011;96(1):53-8.

5. Cacau LT, De Carli E, de Carvalho AM, Lotufo PA, Moreno LA, Bensenor IM, et al. Development and Validation of an Index Based on EAT-Lancet Recommendations: The Planetary Health Diet Index. Nutrients. 2021;13(5).

6. Pan American Health Organization. Pan American Health Organization Nutrient Profile Model. Washington, DC.: PAHO; 2016. Report No.: ISBN 978-92-75-31873-7.

7. Reglamento Sanitario de los Alimentos - DTO. N° 977/96 (Diario oficial 13.05.97), (2019).
